# Osteology of Dwarfgobies *Eviota* and *Sueviota* (Gobiidae: Gobiomorpharia), With Phylogenetic Inferences Within Coral Gobies

**DOI:** 10.1002/jmor.70039

**Published:** 2025-03-26

**Authors:** Diego F. B. Vaz, Christopher H. R. Goatley, Luke Tornabene

**Affiliations:** ^1^ Natural History Museum London UK; ^2^ School of Ocean and Earth Science University of Southampton Southampton UK; ^3^ Australian Museum Research Institute Australian Museum Sydney New South Wales Australia; ^4^ School of Aquatic and Fishery Sciences and Burke Museum of Natural History and Culture University of Washington Seattle Washington USA

**Keywords:** comparative morphology, skeleton, systematics

## Abstract

*Eviota* and *Sueviota* are two genera of cryptobenthic fishes of the family Gobiidae commonly known as dwarfgobies, that collectively contain 142 species. Despite thorough descriptions of the variation of their external morphology, little is known about variations on their skeleton. Combining traditional clearing‐and‐staining technique with computed scanning microtomography, we examined five species of *Sueviota* and 40 species of *Eviota*, representing the two major monophyletic groups in the latter genus, the “branched clade” and “unbranched clade,” named for their pectoral ray morphology. The purpose of this study was to provide generalized descriptions for both genera and highlight potentially phylogenetically informative characters that will aid in future classification of this diverse assemblage of fishes. The posterior portion of the mesethmoid was found to be unossified in eight species of *Eviota* from the unbranched clade. Twenty‐five vertebrae (vs. 26 vertebrae) are present only in species of the unbranched clade of *Eviota,* and it is considered another potential synapomorphy for this clade. Direct contact between the retroarticular and the anterior edge of the interopercle without the retroarticular‐interopercle ligament occurs in all species of *Eviota* and *Sueviota*, being interpreted as a potential synapomorphy grouping these two genera. The posterior edge of the interopercle is notched in all species of *Eviota* and *Sueviota*, as well as in the closely related genera *Bryaninops*, *Pleurosicya*, and *Paragobiodon*. In species of *Sueviota* and the branched clade of *Eviota*, the notch is deep, and there is an additional posteroventral process, forming a wrench‐like posterior edge of the interopercle. This wrench‐shaped interopercle is a potential synapomorphy, grouping *Sueviota* with representatives of the branched clade of *Eviota*. Individual and ontogenetic variations are discussed, including an assessment of the characters previously proposed for characterizing the branched and unbranched clades of *Eviota*.

## Graphical abstract

Skeletal anatomy of dwarfgobies show variation in several taxonomic levels and includes unique of arrangement of the interopercle and how it articulates with the hyoid arch.

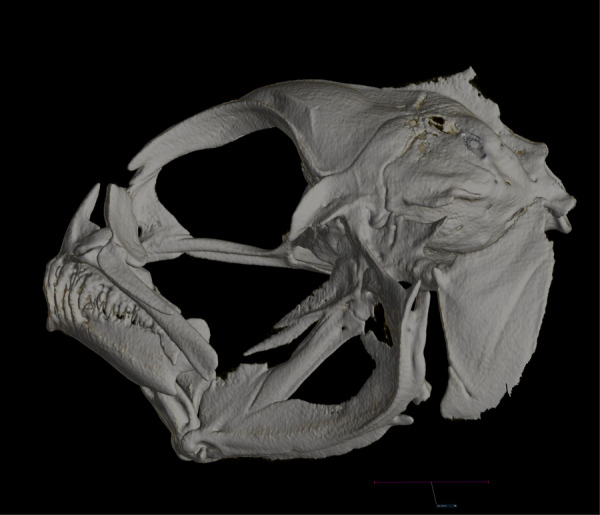

## 1 Introduction

**Figure 1:**
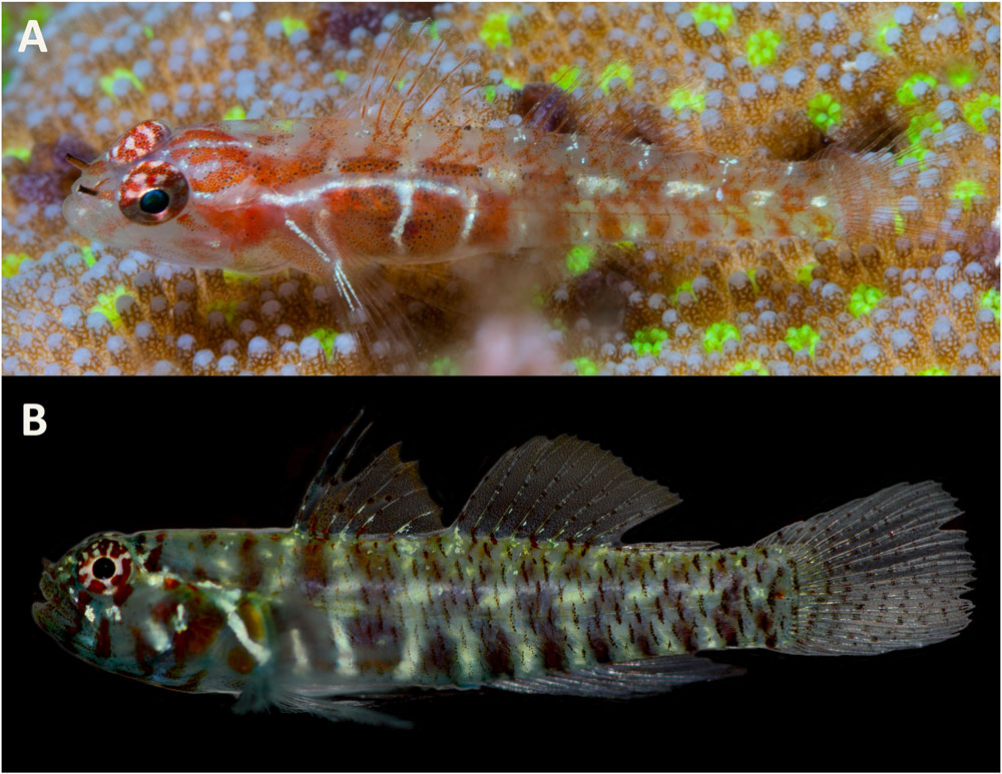
Live specimens of *Sueviota lachneri* (UWFC uncatalogued SL6, 11 mm SL Cendrawish Bay, Indonesia) and *Eviota* cf. *afelei* (MCZ 175058, 16.8 mm SL, Apra Harbor, Guam).

The genera *Eviota* Jenkins 1903 and *Sueviota* Winterbottom and Hoese 1988, commonly known as dwarfgobies (Figure 1), are a diverse group of cryptobenthic fishes in the family Gobiidae distributed across Indo‐Pacific coral reefs. *Eviota* is a speciose genus currently with 133 species (Greenfield et al. 2024) and *Sueviota* with 9 species (Nunes Peinemann et al. 2024). *Eviota* and *Sueviota* are smaller than 30 mm and have a short life span of less than 100 days (Depczynski and Bellwood 2005, 2006; Greenfield 2017), making them some of the smallest and shortest‐lived vertebrates on Earth. The genus *Eviota* has been classified in the “Gobiodon‐lineage” (*sensu* Agorreta et al. 2013; see also Tornabene et al. 2018), along with two other Indo‐Pacific reef‐associated genera *Gobiodon* and *Bryaninops*. Thacker and Roje (2011) provisionally grouped them in an expansive group of genera called the “Coral gobies” based on phylogenetic data for a few genera, or for most other genera, based on ecological similarity or morphological studies by past authors. Collectively, the “Coral gobies” group includes the genera *Bryaninops*, *Gobiodon*, *Kelloggella*, *Larsonella*, *Lobulogobius*, *Lubricogobius*, *Luposicya*, *Minisicya*, *Paragobiodon*, *Phyllogobius*, and *Pleurosicya*. However, the relationships between dwarfgobies and most of these genera have not been empirically tested. Within *Eviota*, two well‐supported monophyletic groups have been recognized based on sequence data from mitochondrial and nuclear genes – one group having completely unbranched pectoral‐fin rays, and a second group that possesses some branched pectoral‐fin rays (Tornabene, Ahmadia, et al. 2013; Tornabene, Chen, and Pezold 2013; Tornabene et al. 2015, 2016).

The external morphology of dwarfgobies has been relatively well studied, particularly regarding meristics and the arrangement of the sensory cephalic pore system, both of which have been relied upon for taxonomic and systematic inferences (e.g., Lachner and Karnella 1978, 1980; Jewett and Lachner 1983; Tornabene, Ahmadia, et al. 2013; Tornabene, Chen, and Pezold 2013; Tornabene et al. 2016; Greenfield and Jewett 2014a, 2014b; Allen and Erdmann 2017; Fujiwara et al. 2020; Winterbottom and Greenfield 2021). By contrast, studies of internal morphology are limited, and are restricted to vertebral counts and tooth morphology (Lachner and Karnella 1978, 1980; Jewett and Lachner 1983; Greenfield et al. 2018). The most comprehensive skeletal assessment of dwarfgobies *Eviota* and *Sueviota* is provided by Gosline (1955) and Winterbottom and Hoese (1988), who provided brief descriptions of their skeleton, respectively. However, complete descriptions of the skeleton of these genera of dwarfgobies are still lacking, as are assessments of ontogenetic and taxonomic variation across their diversity.

The goal of this manuscript is to provide complete and generalized skeletal descriptions for *Eviota* and *Sueviota*, including data from 40 species of *Eviota* and five species of *Sueviota*. Variation within *Eviota* and *Sueviota* is discussed, with particular emphasis on variation across the *Eviota* clades with branched versus unbranched pectoral fins. Variations in the interopercle and pelvic fins are compared in more detail with four other genera that, based on previous studies (Thacker and Roje 2011; Agorreta et al. 2013; Tornabene, Ahmadia, et al. 2013), are closely related to dwarfgobies (*Gobiodon*, *Paragobiodon*, *Pleurosicya*, and *Bryaninops)* and their potential evolutionary implications are discussed.

## 2 Material and Methods

The terminology for skeletal elements follows Hilton (2011), Vaz et al. (2022), and, for the caudal skeleton, Arratia (2008), Schultze and Arratia (2013), Birdsong (1975), Yun et al. (2020), and Reichenbacher et al. (2020). Meristic data follow Fink and Weitzman (1974). The definition of caudal vertebra according to recent skeletal reviews considers only the presence of haemal arches (Hilton 2011; Vaz et al. 2022), therefore, differing from Birdsong (1975) and Winterbottom and Hoese (1988), which consider both the presence of complete haemal arches and the absence of pleural ribs for defining caudal vertebra. *Eviota* and *Sueviota*, as well other coral gobies, have a complete haemal arch on vertebrae 8–10 that surrounds blood vessels, even though these vertebrae are located within the abdominal cavity and have parapophyses that bear ribs. To allow direct comparisons with previous studies and to assign caudal vertebrae only to those posterior to the abdominal cavity, we define caudal vertebra by having a haemal arch ventrally fused to a haemal spine, including the urostyle. The term abdominal vertebra is equivalent to precaudal vertebra of Birdsong (1975). The notational formula for the arrangement of the pterygiophores of the spinous dorsal fin with the underlying vertebrae follows Birdsong (1975). A similar notational method was implemented to both soft dorsal fin and anal fin. All body lengths reported are the standard length (SL).

Preserved specimens were cleared and double stained, adapting the protocols from Dingerkus and Uhler (1977) and Taylor and Van Dyke (1985). Similar to Vaz and Hilton (2023), we modified the time of immersion in the Alcian Blue 8GX solution because of the small size of dwarfgobies. Specimens were examined under a stereomicroscope each hour after immersion in Alcian Blue to ensure the acidic solution did not damage the skeletons. At first sight of staining of pectoral radials or distal caudal radials, specimens were moved to the next step in the protocol. Most specimens had fully stained cartilage after 2‐3 h. Additional skeletal data were obtained by scanning preserved specimens with a Bruker Skyscan 1173 and 1273 computed tomography (micro‐CT) scanners, with the following settings: voltage 25–30 kV; current: 114–150 µA; isotropic voxel resolution: 5–9.5 µm.

Cleared and stained specimens were examined and dissected with binocular dissecting microscopes. Images were captured with a Keyence VHX‐970FN with a motorized stand on the *Z*‐axis to increase the depth of field. Micro‐CT scans were reconstructed using NRecon Reconstruction. Final images were visualized using Amira v. 5.3. Images had their background removed and contrast adjusted using Adobe® Photoshop® 2023 v.24.

Individual skeletal elements were visualized, identified, and compared across species of *Eviota* and *Sueviota*, as well as with representatives of genera *Gobiodon*, *Paragobiodon*, *Bryaninops*, and *Pleurosicya*. A generalized description of the individual bones and cartilages of the skeleton of *Eviota* and *Sueviota* is presented. Structures that presented variation, especially those potentially phylogenetically informative, are described in more detail.

### 2.1 Material Examined

Abbreviations for natural history collections that provided specimens for examination follow Sabaj (2020). The examined material includes representatives of *Eviota*, *Sueviota*, *Gobiodon*, *Paragobiodon*, *Bryaninops*, *Pleurosicya* (Table 1) and other representatives of the family Gobiidae (Appendix 1).

**Table 1:** List of examined materials of *Eviota*, *Sueviota, Gobiodon, Paragobiodon, Bryaninops*, and *Pleurosicya*y.

Species	Specimen number
*Eviota* “branched clade”	
*E. afelei*	UWFC uncatalogued 20, 10.8 mm SL (CT); MCZ 13182, 12.8 mm SL (CT); MCZ 159181, 11.7 mm SL (CT).
*E. albolineata*	MCZ 165595, 16.5 mm SL (CT).
*E. atauroensis*	CAS 248643 (EQ. 2), 16.2 mm SL (CT).
*E. distigma*	CAS 248648 (DIS1), 13.2 mm SL (CT).
*E*. cf. *distigma*	MCZ 13184, 15.8 mm SL (CT).
*E. erdmani*	CAS 248564 (ERD1), 12.6 mm SL (CT).
*E. epiphanes*	MCZ 62357, 16.2 mm SL (CT).
*E. fallax*	CAS 248640 (EFA6), 14.9 mm SL (CT).
*E*. cf. *fasciola*	BMNH 2024.12.13.81, 11 mm SL (CT).
*E*. cf. *hinanoe*	MCZ149605, 11.7 mm SL (CT).
*E. jewettae*	ANSP 150918, 2 specimens, 11.5 mm SL, 10.5 mm SL (CT); CAS 248543 (DS1), 10.3 mm SL (CT); UWFC uncatalogued DS2, 9.2 mm SL (CT).
*E. korechika*	CAS 248562 (EK2), 19.7 mm SL (CT).
*E. latifasciata*	MCZ 162969, 2 specimens, 14.6 mm SL, 14.8 mm SL (C&S).
*E. melasma*	CAS 248649 (EM6), 15.3 mm SL (CT)
*E. monostigma*	UWFC uncatalogued LIRS18‐B2‐107, 19.5 mm SL (CT).
*E. occasa*	UWFC uncatalogued LIRS 19‐B7‐2‐5, 10.6 mm SL (CT).
*E. cf. pinocchioi*	CAS 248642 (TS1), 11.7 mm SL (CT).
*E. prasina*	MCZ 63141, 10.7 mm SL (CT).
*E. pseudaprica*	CAS 248552 (SA3), 13.75 mm SL (CT).
*E*. cf. *punctulata*	ANSP 146494 (paratype), 2 specimens, 18.4 mm SL, 16.5 mm SL (CT).
*E. saipanensis*	MCZ 165036, 17.5 mm SL (CT); BMNH 2024.12.13.13, 15.4 mm SL (C&S).
*E. smaragdus*	MCZ 165021, 15.9 mm SL (CT); BMNH 2024.12.13.8, 20.5 mm SL (C&S).
*E. sparsa*	ANSP 151998 (paratype), 2 specimens, 17.1 mm SL, 13.3 mm SL (CT); ANSP 144484, 2 specimens, 12.74 mm SL, 10.48 mm SL (CT); ANSP 148481, 17.7 mm SL (C&S); ANSP 148485, 16.1 mm SL (C&S); AMI 22611‐058, 2 specimens, 12.7 mm SL, 12.3 mm SL (CT); AMI 17935‐001, 2 specimens, 14.3 mm SL, 13.8 mm SL (CT); AMI 22578‐073, 2 specimens, 14.8 mm SL, 14.7 mm SL (CT); CAS 248650 (PU9), 12.8 mm SL (CT); CAS 248651 (SPAR1), 15.5 mm SL (CT); CAS 248652 (SU4), 15.21 mm SL (CT).
*E. taeiae*	CAS 248041 (ET6), 13.1 mm SL (CT).
*E. teresae*	CAS 248641 (EG5), 15.9 mm SL (CT).
*E. zonura*	MCZ 13181, 11.8 mm SL (CT); MCZ 35973, 16.5 mm SL (CT).
*Eviota* sp.	CAS 248644 (CT), 2 specimens, 9.9 mm SL (SU2), 10.6 mm SL (SU3); CAS 248645 (OA1), 10.6 mm SL (CT).
*Eviota* “unbranched clade”	
*E. atriventris*	UWFC uncatalogued EA23, 14.7 mm SL (CT); UWFC uncatalogued LIRS 18B‐12‐2‐2, 15.3 mm SL (CT).
*E. bifasciata*	UWFC uncatalogued EB20, 14.5 mm SL (CT); UWFC uncatalogued EB23, 14 mm SL (CT).
*E. cometa*	MCZ 162964, 14.1 mm SL (CT); MCZ 162965, 13 mm SL (C&S).
*E. infulata*	CAS 248588 (INF2), 10.7 mm SL (CT).
*E. lachdeberei*	CAS 248577 (EL15), 12.2 mm SL (CT).
*E. cf nigrispina*	UWFC uncatalogued NI12, 10.9 mm SL (CT).
*E. nigriventris*	UWFC uncatalogued NV15, 12.4 mm SL.
*E. pellucida*	BMNH 2024.12.13.16, 17.6 mm SL (CT); BMNH 2024.12.13.18, 15.3 mm SL (C&S).
*E. prasites*	UWFC uncatalogued EPR3, 17.8 mm SL (CT).
*E. rubriceps*	UWFC uncatalogued RU2, 11.9 mm SL (CT).
*E. seebrei*	UWFC uncatalogued EN5, 12.7 mm SL (CT).
*E. shimadai*	CAS 248555 (ESH13), 9.7 mm SL (CT).
*E. sigillata*	CAS 248550 (SI6), 10.7 mm SL (CT).
*E. storthynx*	CAS 248593 (ES5), 13.3 mm SL (CT).
*E. zebrina*	ANSP 138917, 2 specimens, 14.9 mm SL, 14 mm SL (C&S); MCZ 46977, 14.2 mm SL (CT).
*Sueviota*	
*S, lachneri*	ROM 44143 (holotype), 13 mm SL (CT); ROM CS 730 (paratypes), 3 specimens, 12.1‐16.6 mm SL (C&S); ROM 70237, 2 specimens, 19.1 mm SL, 17.1 mm SL (CT); UWFC uncatalogued SL4, 12.05 mm SL (CT); UWFC uncatalogued SL5, 10.9 mm SL (CT).
*S. larsonae*	ROM 41409 (paratype), 2 specimens, 15.7 mm SL, 13.1 mm SL (CT).
*S. aprica*	ROM 43012 (paratype), 10.1 mm SL (CT); ROM 864CS, 13.4 mm SL (C&S); CAS 248538 (SA1), 11.6 mm SL (CT).
*S, atrinasa*	ROM 45185, 2 specimens, 19.5 mm SL, 19 mm SL (CT).
*S. tubicola*	UWFC uncatalogued WT1, 12 mm SL (CT); UWFC uncatalogued WT2, 15.6 mm SL (CT).
***Bryaninops***.	
*B. amplus*	MCZ 84525, 14.9 mm SL (C&S).
***Gobiodon***	
*G. quinquestrigatus*	MCZ 36717, 31.3 mm SL (CT)
*G. rivulatus*	MCZ 63137, 2 specimens, 22.5 mm SL (C&S), 22.3 mm SL (CT);
*Gobiodon sp*.	MCZ 175091, 22 mm SL (CT).
***Paragobiodon***	
*P. echinocephalus*	MCZ 13039, 19.8 mm SL (CT).
*P. modestus*	MCZ 15473, 19.5 mm SL (CT).
***Pleurosicya***	
*P. micheli*	MCZ 162627, 14.9 mm SL (C&S).

Abbreviations: C&S, cleared‐and‐stained specimen; CT, specimen scanned with microcomputed tomography.

## 3 Results

### 3.1 Neurocranium

**Figure 2:**
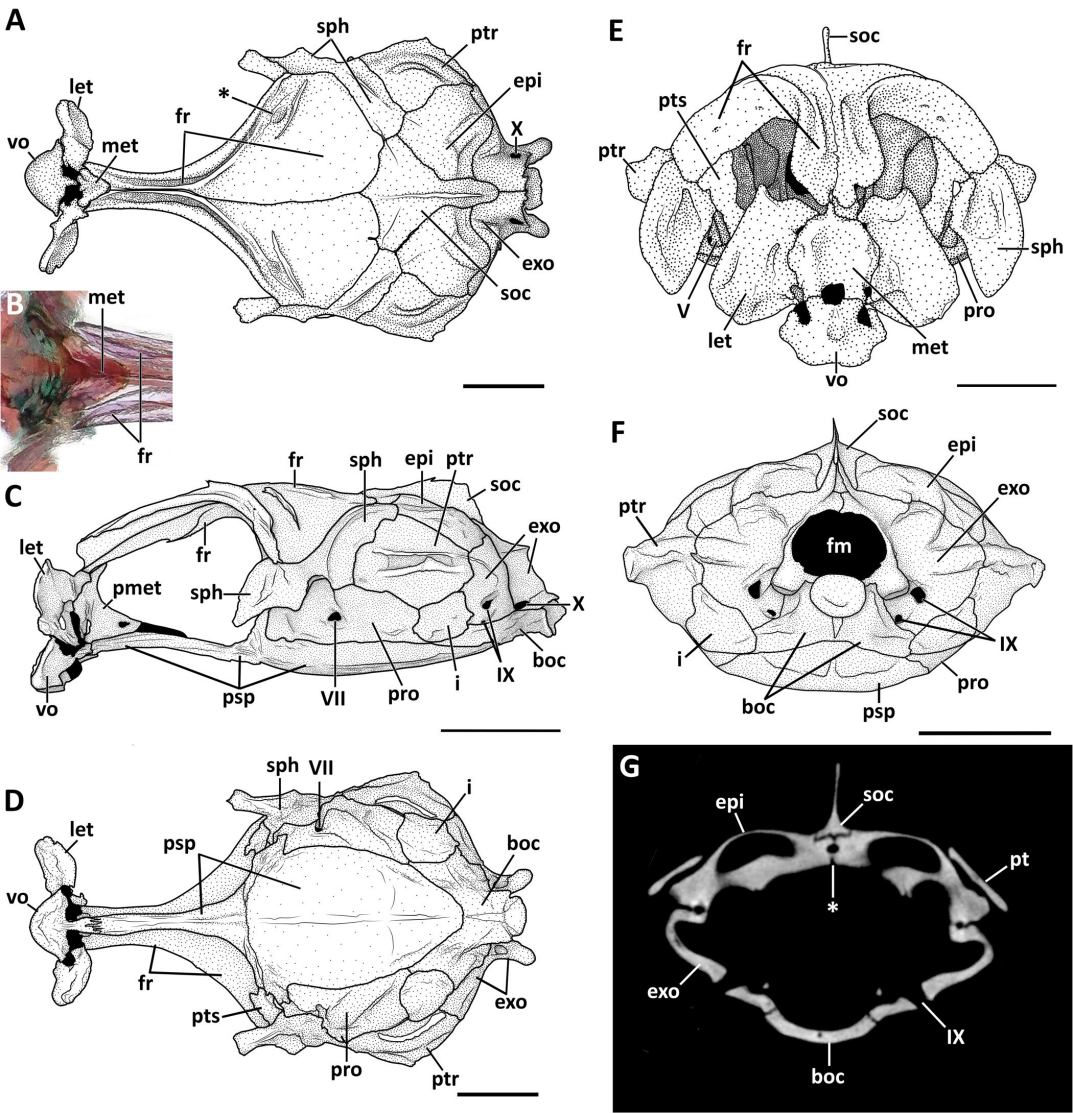
Neurocranium of *Eviota* and *Sueviota*, represented by *Eviota sparsa* (A–E) and *Eviota zonura* (F). (A) Illustration of the dorsal view of the neurocranium of *Eviota sparsa* (ANSP 148485, 16.1 mm SL) after cleared‐and‐stained preparation. Asterisk represents the groove on the neurocranium which supports the cephalic pores of the lateralis sensorial system. (B) Close‐up image of *Eviota sparsa* (ANSP 148485, 16.1 mm SL) demonstrating the arrangement between the dorsal margin of the mesethmoid and the anterior margins of the frontals. (C) Neurocranium of *Eviota sparsa* (ANSP 148485, 16.1 mm SL) in lateral view after clearing‐and‐staining preparation. (D) Illustration of the ventral view of the neurocranium of *Eviota sparsa* (ANSP 148485, 16.1 mm SL) after clearing‐and‐staining preparation. (E) Illustration of the neurocranium of *Eviota sparsa* (ANSP 151998, paratype, 17 mm SL) in anterior view after CT‐scanning. (F) Occipital view of the neurocranium of *Eviota sparsa* (ANSP 148485, 16.1 mm SL) after clearing and staining. (G) Cross‐section of the neurocranium of *Eviota zonura* (MCZ 35973, 16.5 mm SL) at the level of the openings of the glossopharyngeal foramina. Asterisk indicates the medial margins of the epioccipital bones contacting their antimeres below the supraoccipital. Scale bar, 500 µm. boc, basioccipital; epi, epioccipital; exo, exoccipital; fr, frontal; fm, foramen magnum; i, intercalar; let, lateral ethmoid; met, mesethmoid; pmet, posterior portion of the mesethmoid; pro, prootic; psp, parasphenoid; ptr, pterotic; pts, pterosphenoid; soc, supraoccipital; sph, sphenotic; vo, vomer; V, foramen for the trigeminal nerve; VII, foramen for the hyomandibular branch of the *fascialis* nerve; IX, foramen for the glossopharyngeal nerve; X, foramen for the *vagus* nerve.

The neurocranium of *Eviota* and *Sueviota* is well‐ossified and elongate; its length is twice that of its greatest width, and also slightly flattened, with its greatest width twice that of its depth (Figure 2).

#### 3.1.1 Ethmoidal Region

The ethmoidal region of the neurocranium comprises a singular vomer, a pair of lateral ethmoids, and the mesethmoid.

**Figure 3:**
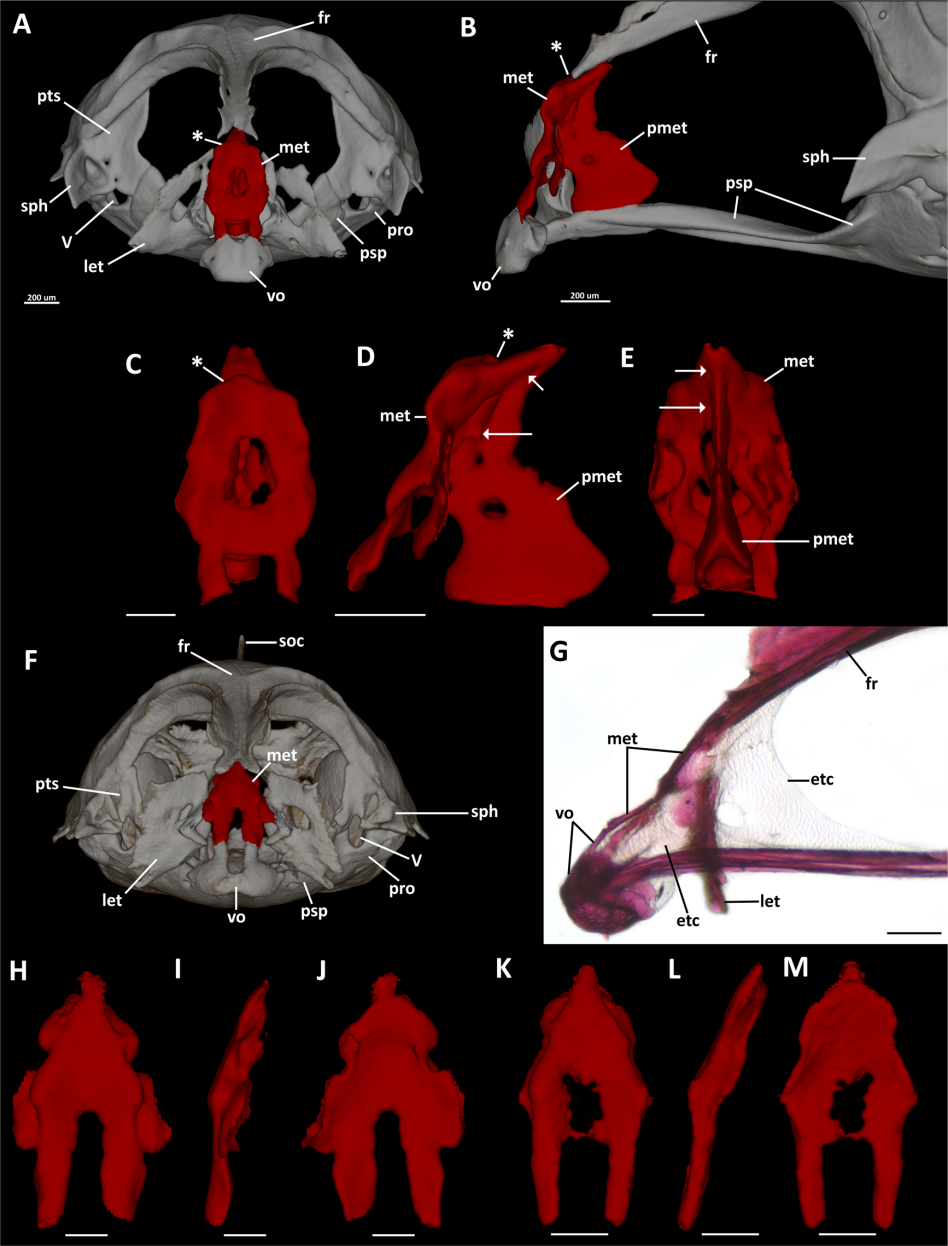
Comparative morphology of the mesethmoid in *Eviota* and *Sueviota*. Figures (A–E) represent the condition observed in *Sueviota* and most examined species of *Eviota*. Figures (F–M) illustrate the condition observed in *Eviota prasites*, *E. bifasciata*, *E. atriventris*, *E. lachdeberei*, *E. nigriventris*, *E. infulata*, and *E. seebrei*. The mesethmoid bone is illustrated in red in all images. (A) Anterior view of the neurocranium of *E. atauroensis* (CAS 248643). (B) Lateral view of the ethmoidal region of the neurocranium of *E. atauroensis* (CAS 248643), highlighting the shape and extent of the mesethmoid. Left antimere of lateral ethmoid removed. (C–E) Digitally dissected (i.e., segmented) mesethmoid of *E. atauroensis* (CAS 248643) in anterior (C), lateral (D), and occipital (E) views. Asterisk represents the anterodorsal ridge of the mesethmoid. White arrows indicate sutures between the posterior and the anterior portions of the mesethmoid. (F) Anterior view of the neurocranium of *Eviota prasites* (UWFC uncatalogued EPR3). (G) Lateral view of the ethmoidal region of the neurocranium of *E. pellucida* (BMNH 2024.12.13.18), highlighting the shape and extent of the mesethmoid. Left antimere of lateral ethmoid removed. (H–J) Digitally dissected mesethmoid of *E. prasites* (UWFC uncatalogued EPR3) in anterior (H), lateral (I), and occipital (J) views. (K–M) Digitally dissected mesethmoid of *E. lachdeberei* (CAS 248577) in anterior (K), lateral (L), and occipital (M) views. Scale bar, 200 µm. fr, frontal; let, lateral ethmoid; met, mesethmoid; pmet, posterior portion of the mesethmoid; pro, prootic; psp, parasphenoid; pts, pterosphenoid; sph, sphenotic; vo, vomer; V, foramen for the trigeminal nerve.

**Vomer**. The vomer forms a cap, encapsulating the remains of the ethmoidal plate (Figures 2A,C–E and 3A,B,F,G). The vomer is rounded anteriorly, with two posterior acute edges. The posterior margin of the vomer is convex laterally and has a rectangular projection that extends posteriorly, with longitudinally‐oriented indentations that form a suture with the parasphenoid (Figure 2D).

**Lateral ethmoid**. In all species, the pair of lateral ethmoid bones are laminar, trapezoidal, vertically oriented, and slightly curved posteriorly (Figures 2C–E; 3A,F; and 4). The ventrolateral edge of the lateral ethmoid is expanded and elliptical, forming a condyle to articulate with the infraorbital one bone (Figure 2C).

**Figure 4:**
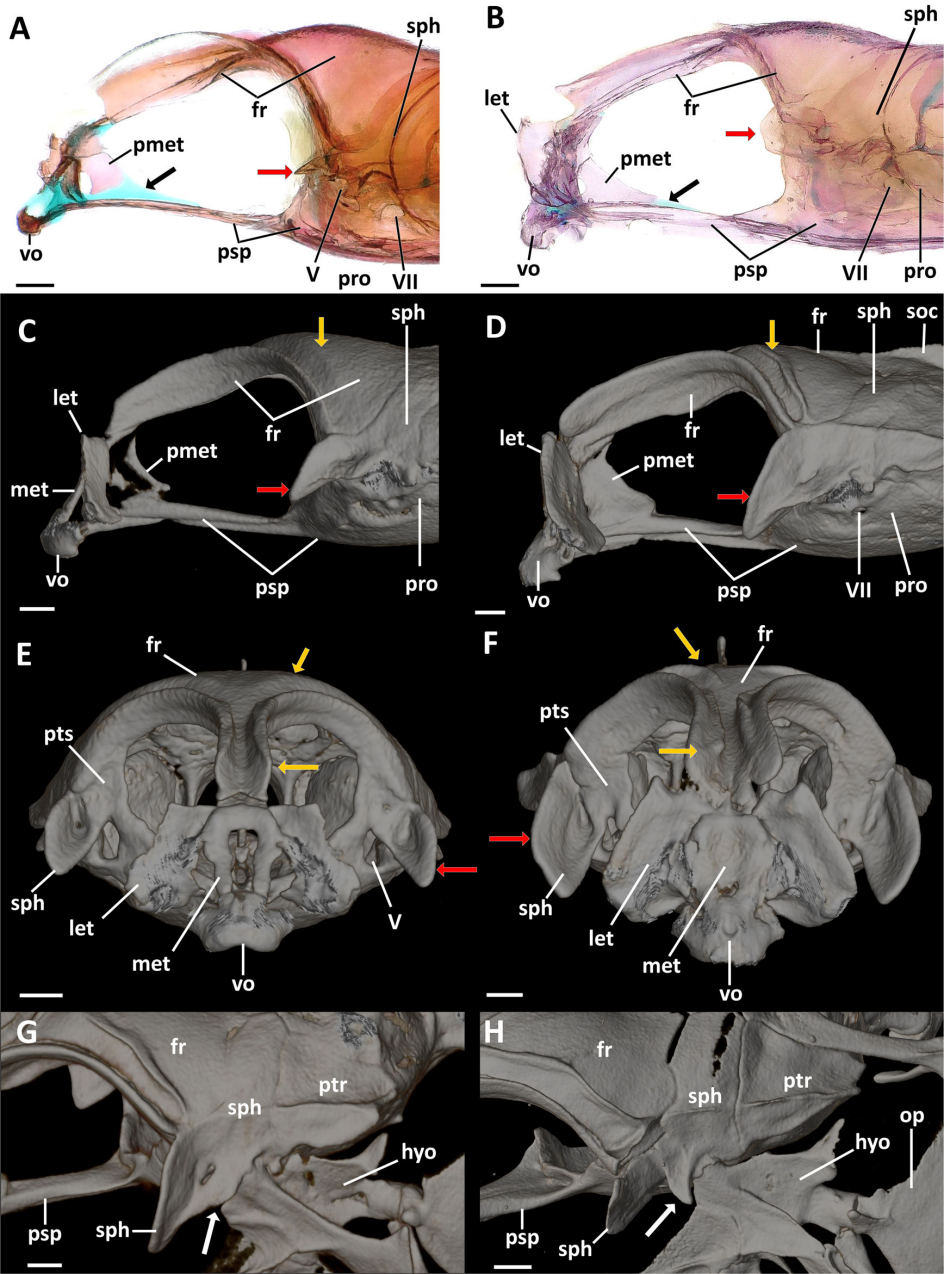
Neurocranial variations across diversity of dwarfgobies. (A) Lateral view of the anterior region of the neurocranium of *Eviota zebrina* (ANSP 138917, 14.9 mm SL). (B) Lateral view of the anterior region of the neurocranium of *Eviota cometa* (MCZ 162965, 13 mm SL). (C–F) Ontogenetic variation in the neurocranium of *Eviota sparsa* in lateral (C, D) and anterior (E, F) views. (C, E) Paratype of *Eviota sparsa* (ANSP 151998 13.3 mm SL). (D, F, G) Paratype of *Eviota sparsa* (ANSP 151998, 17.1 mm SL). (G) Dorsolateral view of the sphenotic articulation with the hyomandibula. White arrow indicates the base of the sphenotic process without a posterior process. (H) Dorsolateral view of the sphenotic articulation with the hyomandibula of *Eviota saipanensis* (MCZ 165036, 17.5 mm SL). White arrow indicates the base of the sphenotic process with a distinct posterior process. Red arrows indicate variation in shape and relative size of the anterior process of the sphenotic. Yellow arrows indicate variation of the frontals. Black arrows indicate variation of the extent of the ossification of the posterior portion of the mesethmoid. Scale bar, 200 µm. boc, fr, frontal; hyo, hyomandibula; let, lateral ethmoid; met, mesethmoid; op, opercle; pmet, posterior portion of the mesethmoid; pro, prootic; psp, parasphenoid; ptr, pterotic; pts, pterosphenoid; soc, supraoccipital; sph, sphenotic; vo, vomer; V, foramen for the trigeminal nerve; VII, foramen for the hyomandibular branch of the *fascialis* nerve; IX, foramen for the glossopharyngeal nerve; X, foramen for the *vagus* nerve.

**Mesethmoid**. The mesethmoid is a complex shaped bone that ossifies around the median region of the ethmoid cartilage. The mesethmoid of most examined species of *Eviota* and *Sueviota* has two portions (Figure 3). The anterior portion of the mesethmoid is laminar, wide, and drop‐shaped, positioned between the lateral ethmoids (i.e., anterior to the ethmoid cartilage), with an acute dorsal edge that extends posterodorsally between the anterior edges of the frontal (which is the opposite of that described for *Trimma*, where mesethmoid extensions are lateral to the anterior edge of frontals; see Winterbottom 2003). The dorsal region of the mesethmoid has a transversal ridge (Figure 3A,C,D). Ventrally, the anterior portion of the mesethmoid has two rectangular projections that contact the dorsal margin of the vomer. The posterior portion of the mesethmoid is laminar, with convex anterior margin, concave posterior margin, and a rounded fenestra in its middle (Figure 4B,D). In most examined specimens (e.g., *Eviota erdmani*, CAS 248564 [ERD1]; *Eviota sparsa*, paratype ANSP 1519998), the anterior and posterior portions of the mesethmoid are continuous, but in *Sueviota atrinasa* (ROM 45185), *Eviota atauroensis* (CAS 248643 [EQ. 2]; Figure 3B,D), *E. storthynx* (CAS 248593 [ES5]), and *E*. cf. *sparsa* (CAS 248644 [SU2, SU3]; CAS 248652 [SU4], ANSP144484) sutures between these portions are visible (this separation is also visible in the CT‐stacks as well).

The mesethmoid of *Eviota prasites* (UWFC uncat EPR3), *E. pellucida* (BMNH 2024.12.13.16, BMNH 2024.12.13.18), *E. bifasciata* (UWFC uncat EB20, EB23), *E. atriventris* (UWFC uncat LIRS18B12‐2‐2), *E. lachdeberei* (CAS 248577 [EL15]), *E. nigriventris* (UWFC uncat NV15), *E. infulata* (CAS 248588 [INF2]), and *E. seebrei* (UWFC uncat EN2), conversely, completely lack an ossified posterior portion (Figure 3F–M). This region remains cartilaginous, with a robust, triangular ethmoid cartilage that extends from the posterior surface of the mesethmoid bone and the anterior tip of the frontals to the middle of the parasphenoid (Figure 3G). An oval fenestra is present in the middle of the ethmoid cartilage. The mesethmoid consists only of the laminar bone, transversely oriented between the lateral ethmoids. The dorsal region of the mesethmoid of these eight species lacks the dorsal ridge and has a relatively more acute dorsal edge than the other species of *Eviota* and *Sueviota*.

*E. prasites*, *E. infulata*, and *E. seebrei* have a pair of posterolateral rectangular projections on the middle of the posterolateral margins of the mesethmoid (Figure 3H,J), whereas in *E. pellucida*, *E. bifasciata*, *E. atriventris*, *E. lachdeberei*, and *E. nigriventris* this region has only a short triangular expansion. Additionally, in *E. pellucida*, the middle of the anterolateral margins of the mesethmoid have prominent triangular projections. In the other species, this region has a shallow semicircular bulge Figure 3H–M).

#### 3.1.2 Orbital Region

The orbital region of the neurocranium contains a pair of frontals, pterosphenoid and parasphenoid. The orbitosphenoid and basisphenoid are absent in dwarfgobies.

**Frontal.** Frontals are long, extending through more than two‐thirds of the dorsal surface of the neurocranium (Figure 2A). The anterior margins of the frontals are slightly convex and anterolaterally oriented, extending over the lateral margins of the mesethmoid (Figure 2B). The anterior region of each frontal is narrow and elongated, expanding laterally by the posterior region of the orbital region, reaching the base of the anterior process of the sphenotic (Figure 2A,C). The posterior margins of the frontals are convex, with the medial portion sitting atop of the anterior margin of the supraoccipital (Figure 2A,C). Medial margins of frontals contact each other sagitally, but there is no fusion between the antimeres of frontals. The groove for the passage of supraorbital canals has conspicuous lateral walls throughout the anterolateral margins of frontals (Figures 2A,C; 3A,F; and 4A–D). Posteromedial walls, when present, are restricted to the posterior region of frontals (Figures 2A,C and 4D,F). Occurrence of medial walls of the groove of the supraorbital canal are often observed in larger specimens (see variation in *E. sparsa*; Figure 4C–F); however, in some small species (e.g., *Eviota cometa*), this feature was never observed.

**Parasphenoid.** The parasphenoid is the longest bone in the skull of *Eviota* and *Sueviota*, extending along almost the entire ventral surface of the neurocranium (Figure 2C,D). The anterior region is narrow, slender, forming an indented suture with the posterior region of the vomer. The dorsolateral margin of the anterior region of the parasphenoid is convex. In the posterior region of orbits, the parasphenoid expands dorsolaterally, forming the ascending process of the parasphenoid. The ascending process is broad ventrally and tapers dorsally, where it reaches the anterior surface of the prootic (Figure 2C). From the base of the parasphenoid's ascending process, the lateral margins of the parasphenoid are concave and extend posteromedially, forming a sagittal suture with the anteroventral surface of the basisphenoid (Figure 2D). A thin ridge is present centrally in the ventral surface of the parasphenoid (Figures 2D and 3B).

**Pterosphenoid.** The pterosphenoid is a small, laminar, rhomboidal bone, positioned on the posterodorsal region of the orbital region, ventral to the frontals, medial to the anterior process of the sphenotic, and dorsal to the parasphenoid (Figures 2E; 3A,F; and 4E,F). The ventral margin of the pterosphenoid is concave, delimiting dorsally the foramen for passage of the trigeminal nerve. The ventromedial edge of the pterosphenoid has a small, rectangular projection that contacts the ascending process of the parasphenoid. The ventrolateral region of the pterosphenoid can have small, circular foramina for passage of sensorial nerves from the trigeminal branch. The number of foramina varies from one to two, with such variation happening even individually (e.g., *E. atauroensis* CAS 248643 [EQ. 2], Figure 3A, with two foramina on the right pterosphenoid, but a single opening on the left side).

#### 3.1.3 Otic (Auditory) Region

The otic region of the neurocranium comprises three pairs of bones: a pair of sphenotics, a pair of prootics, and a pair of pterotics.

**Sphenotic.** The sphenotic is located in the anterodorsal region of the optic region (Figures 2, 3, 4). The sphenotic is angular; its dorsal region is trapezoidal, posterodorsally oriented, with the anterior margin covered by the posterior margin of the frontals, forming a sagittal suture (Figure 2C). The posterior margin of the sphenotic projects posteromedially, contacting the pterotic posteriorly, with its posteromedial edge contacting the anterior margins of both epioccipital and supraoccipital (Figure 2A,C). The ventral region is rectangular, with a trapezoidal process projecting anteriorly. The anterior edge of the anterior process of the sphenotic is angular, with the exception of *E. cometa*, which has a blunt anterior edge of the sphenotic process (Figure 4B). The length of the anterior process of the sphenotic varies from 0.2 (*Eviota melasma*) to 0.8 (*Eviota sparsa*) times the length of the ventral margin of the sphenotic. In *E. sparsa* (only species with an available growth series), the length of the anterior process of the sphenotic also varies ontogenetically, with the smaller specimens (AMI 22611‐058, 12.3 mm SL, 12.7 mm SL) having the shortest anterior process of the sphenotic (0.5 times the length of the ventral margin of the sphenotic), whereas the largest specimens (paratype ANSP 151998, 17 mm SL) bear the longest anterior processes (0.8 times the length of the ventral margin of the sphenotic; Figure 4E,F). The posterior margin of the anterior process of the sphenotic extends posteriorly to meet a shallow, rounded depression for articulation with the anterior articular head of the hyomandibula (Figure 4G). *Eviota afelei* (MCZ 13182, MCZ 159181, UWFC uncatalogued 20), *E. saipanensis* (MCZ 165036), *E. atauroensis* (CAS 248643 [EQ. 2]), *E. distigma* (CAS 248648 [DIS1], MCZ 13184), *E. melasma* (CAS 248649 [EM6]), *E. epiphanes* (MCZ 62357), *E. smaragdus* (MCZ 165024), *E. cf. fasciola* (BMNH 2024.12.13.81), *E*. cf. *hinanoe* (MCZ 149605), *E. punctulata* (ANSP 146494), *E. cometa* (MCZ 162964, MCZ 162965), and *E. zebrina* (ANSP 138917, MCZ 46977) have an additional triangular process present on the middle of the dorsolateral margin of the sphenotic, dorsal to the articular region for the hyomandibula (Figure 4H).

**Prootic.** The prootic is trapezoidal, extending through the anteroventral region of the lateral surface of the optic region. The anterior region of dorsal margin of prootic is straight and horizontally oriented. The posterior region of the dorsal margin of the prootic projects posteroventrally, meeting the intercalar on the posterior edge. The ventral margin of the prootic is slightly convex, horizontally oriented, and partially covered by the lateral margin of the parasphenoid. On the anterior region of the prootic, there is the foramen for passage for the branches of the trigeminal nerve (Figures 2E; 3A,F; and 4E). The foramen for the passage of the motor branch of the hyomandibular nerve is located close to the middle of the prootic (Figures 2C and 4A,D).

**Pterotic.** The pterotic is a large, trapezoidal bone that covers the posterodorsal region of the optic region (Figure 2A,C,D). The anterodorsal margin extends posteromedially, forming the suture with the sphenotic. The posterodorsal margin is concave and projects posterolaterally, forming the suture with the epioccipital (Figure 2A). The anteroventral margin of the pterotic is slightly concave and projects posteromedially. The posteroventral margin is straight and projected posterolaterally. The region where both antero‐ and posteroventral margins meet is covered by the intercalary (Figure 2C,D,F). In the middle of the pterotic, marking the transition between dorsal and lateral surfaces, there is a large ridge that crosses most of the pterotic, extending to the exoccipital (Figure 2F). Anteriorly, this ridge forms a groove with a distinct depression where the posterior articular head of the hyomandibular attaches (Figure 2C).

#### 3.1.4 Occipital Region

The occipital region of the neurocranium of *Eviota* and *Sueviota* comprises a singular supraoccipital, a pair of epioccipitals, and pair of exoccipitals, a pair of intercalars, and a singular basioccipital.

**Supraoccipital.** The supraoccipital is a median bone, rhomboidal shaped anteriorly, with an elongated posterior projection (Figure 2A). The anterior margin of the supraoccipital is convex, forming a wedge between the posterior margins of the frontals. The anterolateral edges of the supraoccipital are broad, forming a suture with the sphenotic. The posterior margins of the supraoccipital are straight, posteromedially oriented, forming sutures with the epioccipitals. The posterior edge of the supraoccipital is rounded and extends over the medial margins of the exoccipital (Figure 2A,F,G). Medially, a narrow, laterally compressed supraoccipital ridge is present. The shape of the supraoccipital ridge varies from triangular to trapezoidal, and its greatest depth is approximately one‐fifth of the depth of the neurocranium.

**Epioccipital.** The epioccipitals are paired, rhomboidal bones positioned posterolaterally from the supraoccipital (Figure 2A,C,F). The anteromedial margins of the epioccipitals are straight and contact the supraoccipital. The posteromedial margins of the epioccipitals contact medially, dorsally covered by the posterior projection of the supraoccipital (Figure 2F,G). The anterolateral margins of the epioccipitals are gently concave, forming a sagittal suture with the pterotic. The posterior margins are convex and form a suture with the dorsal margins of the exoccipital. The posteromedial surface of the epioccipital is flat, forming the site of attachment for the dorsal limb of the postemporal bone.

**Exoccipital.** Exoccipitals are paired, vertically oriented, and located on the posterior region of the neurocranium (Figure 2A,C,D). The posterior margins of exoccipitals are convex and delimit the dorsolateral walls of the foramen magnum (Figure 2F). The medial margins of exoccipitals are straight and contact each other, forming a straight suture. The anterior region of the medial margins is covered by the posterior projection of the supraoccipital. The anterodorsal margins of exoccipitals are convex, contacting the epioccipital. The anterolateral margins are concave and vertically oriented. The anteroventral edge of the exoccipital has a deep notch and is covered by the intercalary bone (Figure 2C; the shape of the margin seen as transparency in cleared‐and‐stained specimens). The ventral margins of exoccipitals are straight and extend posteromedially, forming a suture with the basioccipital (Figure 2C,F). The posteroventral edge of each exoccipital projects posteriorly, forming a condyle with a flattened surface that articulates with the corresponding parapophysis of the first vertebra (Figure 2A,C,F). Three foramina are observed in the exoccipital: a vagal foramen (X) is located close to the posterior margin, at the level of the articular projections. Anteriorly from the vagal foramen, a pair of glossopharyngeal (IX) foramina is present.

**Intercalar.** The intercalar is a dermal rhomboidal bone where sits the ventral limb of the postemporal. The intercalar is a thin lamina covering the sutures of the pterotic, prootic, exoccipital, and basioccipital (Figure 2C,D,F). Gosline (1955) described the intercalar ( = opisthotics in Gosline 1955) of *E. epiphanes* as a small, triangular bone. In all examined species of *Eviota* and *Sueviota* (including *Eviota epiphanes*, MCZ 62357), however, the intercalar is a large, laminar, trapezoidal bone (Figure 2). The shape described by Gosline (1955) matches the cartilaginous gap among the prootic, exoccipital, pterotic, and basioccipital that the intercalar covers (visible through transparency in cleared‐and‐stained specimens), but the extent of the intercalar extends over these other bones too (Figure 2C,D,F).

**Basioccipital.** The median basioccipital bone is located posteromedially on the ventral surface of the neurocranium (Figure 2C,D,F). A centrum‐like joint for articulating with the first vertebral centrum is located at the posterior edge of the basioccipital. The anterior margin of the basioccipital is convex, with a deep medial indentation, and extends laterally to meet the intercalar. The lateral region of the anterior margin of the basioccipital forms a suture with the posterior margin of the prootic. The lateral margins of the basioccipital are slightly concave, projecting posteromedially, forming a suture with the ventral margin of the exoccipitals.

### 3.2 Jaws, Hyosympletic Arch, and Infraorbital One

**Figure 5:**
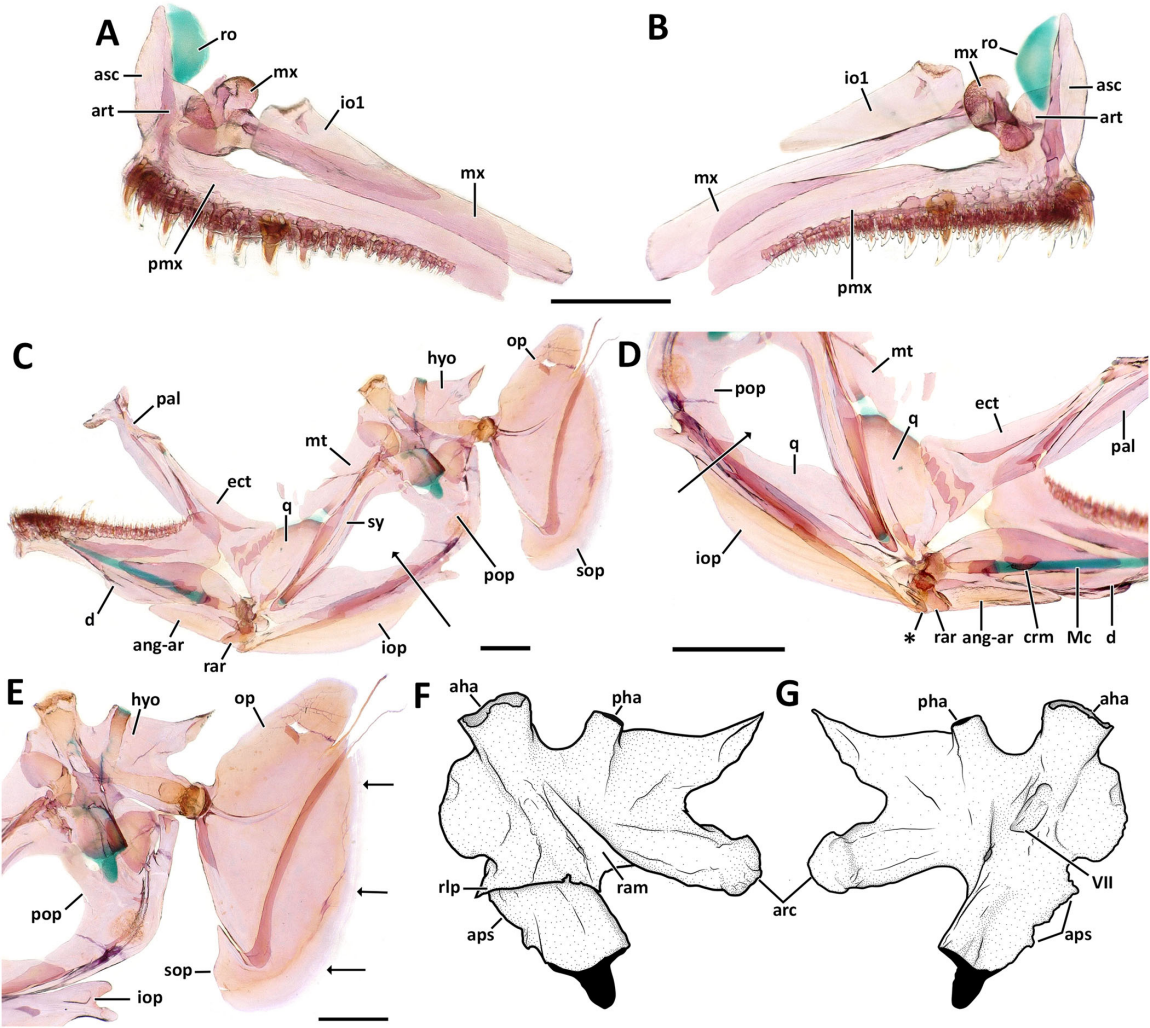
Oral jaws, suspensorium, opercular series, and infraorbital of *Eviota* and *Sueviota*, represented by *Eviota sparsa* (ANSP148485, 16.1 mm SL). (A, B) Upper oral jaws and infraorbital one bone in lateral (A) and medial (B) views. (C) Lower jaw, suspensorium, and opercular series in lateral view. Black arrow indicates the non‐osseous gap between the sympletic and the preopercle. (D) Medial view of the upper oral jaw articulating with the lower jaw. Asterisk indicates the anterior edge of the interopercle contacting the retroarticular. Black arrow indicates the non‐osseous gap between the sympletic and the preopercle. (E) Higher magnification of the hyomandibula, preopercle, opercle, subopercle, and posterior edge of the interopercle. Black arrows indicate the continuous and convex posterior margin of the subopercle. (F, G) Illustration of the isolated hyomandibula after clearing‐and‐staining in lateral (F) and medial (G) views. Cartilages are represented in black. Scale bar, 500 µm. aha, anterior head of articulation of the hyomandibula; ang‐ar, angulo‐articular; arc, condyle for articulation with the preopercle; aps, anteroventral process for articulation with the sympletic; art, articular process of the premaxilla; asc, ascending process of the premaxilla; crm, coronomeckelian; d, dentary; ect, ectopterygoid; hyo, hyomandibula; io1, infraorbital one; iop, interopercle; Mc, Meckel's cartilage; mt, metapterygoid; mx, maxilla; op, opercle; pal, palatine; pha, posterior head of articulation of the hyomandibula; pmx, premaxilla; pop, preopercle; q, quadrate; ram, ridge for attachment of muscular fibers of the *adductor mandibulae pars malaris*; rar, retro‐articular; rlp, ridge for attachment of the insertion of the *levator arcus palatine* muscle; ro, rostral cartilage; sop, subopercle; sy, sympletic.

**Figure 6:**
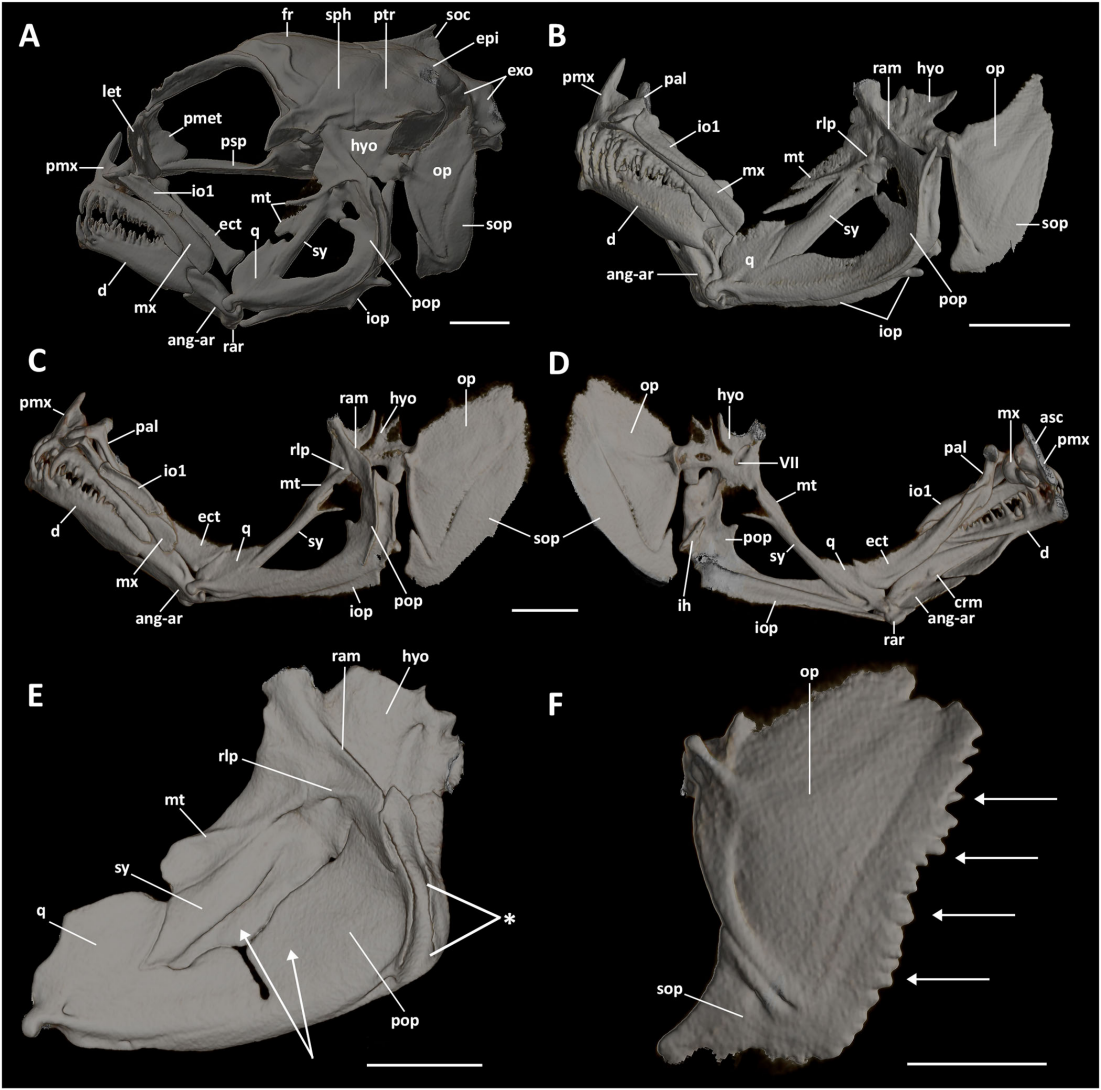
(A) Neurocranium, oral jaws, suspensorium, opercular series, and infraorbital of *E. atauroensis* (CAS 248643, 16.2 mm SL) in lateral view. (B) Lateral view of the oral jaws, suspensorium, opercular series, and infraorbital of *Eviota pseudaprica* (CAS 248552, 13.7 mm SL). (C, D) Oral jaws, suspensorium, opercular series, and infraorbital of *Eviota bifasciata* (UWFC uncatalogued EB 20, 14.5 mm SL) in lateral (C) and medial (D) views. (E) Lateral view of the quadrate, sympletic, hyomandibula, and preopercle of *Eviota* cf. *distigma* (MCZ 13184, 15.8 mm SL). White arrows indicate the expansion of the sympletic and the preopercle enclosing the corresponding non‐osseous gap observed in other species. The asterisk indicates the widened regions where the preopercular pores sit. (F) Details of the opercle and subopercle of *Sueviota tubicola*. White arrows indicate the serrated posterior margin of the subopercle (UWFC uncatalogued WT2, 12.04 mm SL). Scale bar, 500 µm. ang‐ar, angulo‐articular; asc, ascending process of the premaxilla; crm, coronomeckelian; d, dentary; ect, ectopterygoid; epi, epioccipital; exo, exoccipital; fr, frontal; hyo, hyomandibula; io1, infraorbital one; iop, interopercle; let, lateral ethmoid; Mc, Meckel's cartilage; mt, metapterygoid; mx, maxilla; pmet, posterior portion of the mesethmoid; pal, palatine; pmx, premaxilla; pop, preopercle; psp, parasphenoid; ptr, pterotic; op, opercle; q, quadrate; ram, ridge for attachment of muscular fibers of the *adductor mandibulae pars malaris*; rar, retro‐articular; rlp, ridge for attachment of the insertion of the *levator arcus palatine* muscle; sph, sphenotic; soc, supraoccipital; sop, subopercle; sy, sympletic.

**Premaxilla.** The premaxilla is a laterally compressed bone, with its anterior region curving anteriorly to meet with its antimere (Figures 5A,B and 6A–D). The posterior region of the premaxilla has an elongate, thin dorsal flange. The posterior tip of the premaxilla is mostly rounded and wide at the attachment of the supralabial ligament (Datovo and Vari 2013). The ascending process of the premaxilla is elongated; its length is approximately half of the length of the premaxilla. The articular process of the premaxilla is trapezoidal and approximately half the length of the ascending process of the premaxilla. The dorsal margin of the articular process originates laterally from the vertical middle of the lateral margin of the ascending process (Figure 5A,B). The ventral margin of the premaxilla bears two types of teeth: the more external lateral row has 4–8 large canine teeth, projecting ventrally and well‐spaced from each other, the anteriormost positioned ventral to the ascending process, and posteriormost positioned in the mid‐length of the premaxilla. Two to three inner medial tooth rows of small straight, canine teeth spread uniformly from the symphysis to the posterior region of the premaxilla (usually with more than 30 teeth). *Eviota maculosa*, *E. tigrina, E. punctulata*, and *E*. cf. *punctulata* (UW 157173 in Tornabene et al. 2018) have a similar arrangement of tooth rows in the premaxilla, but its teeth are spatulated with tricuspid tips (Greenfield et al. 2018, Figure 5; Tornabene et al. 2018, Figures 7, 8 and 8C). Similar to an observation by Greenfield et al. (2018), specimens of *Eviota* cf. *punctulata* (i.e., not from the type locality) examined in this study (ANSP 146494) were have only caniniform teeth.

**Maxilla**. The maxilla is an elongated bone, semi‐cylindrical anteriorly, but laterally compressed and twice as deep posteriorly than at the anterior region of the maxilla (Figures 5A,B and 6A–C). A complex cup‐shaped head forms the anterior tip of the maxilla (Figure 5B). The anterior surface of the head articulates with the posterior surface of articular process of the premaxilla and the rostral cartilage. The anteromedial edge of the maxillary head is rectangular and projects medially, serving as anchor point for the maxilla‐ethmoid ligament and the endomaxillar ligament.

**Dentary**. The dentary has a broadly triangular shape, being anteromedially curved, with its posterior region twice as deep as the anterior (Figures 5C and 6A,B,D). The dentary bears a distinct, dorsally rounded coronoid process on its posterodorsal edge. The posterior margin of the dentary has a semi‐circular concavity ventrally, at the level of where it contacts with the anguloarticular bone. The medial margin of dentary has a groove ventro‐medially where sits the anterior region of the remnants of the Meckel's cartilage. Dentition of the dentary is similar to that described for the premaxilla: an outer lateral row with three to four large, dorsally directed canine teeth, positioned along the anterior third of the dentary (Figures 5, 6 and 6A–C). Internal medial teeth comprised by one to two rows of straight, canine teeth, approximately one‐half the length of the outer teeth. *Eviota maculosa*, *E. tigrina, E. punctulata*, and *E*. cf. *punctulata* (UW 157173 in Tornabene et al. 2018; see also Greenfield et al. 2018, Figure 5) have a similar arrangement of tooth rows on the dentary, but its teeth are tricuspid‐shaped.

**Anguloarticular**. The anguloarticular bone has a trapezoidal shape with a deep indentation in its antero‐ventral edge (Figures 5C and 6A–D). The dorsal margin of the anguloarticular is convex, the ventral margin is straight (Figure 5D). The depth of anguloarticular is half the depth of the posterior region of the dentary. The posterodorsal edge of the anguloarticular is robust and cup‐shaped, forming the site of articulation with the quadrate. On the central region of posteromedial surface, a shallow ossified strut is continuous with the posterior edge of the Meckel's cartilage.

**Coronomeckelian**. The coronomeckelian bone is a small, comma‐shaped bone attached to the posterodorsal region of medial surface of the Meckel's cartilage (Figures 5D and 6D).

**Retroarticular**. The retroarticular is a pyramidal‐shaped bone positioned on the posteroventral edge of the anguloarticular (Figure 5C,D). In all examined *Eviota*, the posteroventral edge of retroarticular has a small projection that directly contacts the anterior tip of the interopercle, lacking the retroarticular‐interopercle ligament usually observed in other Gobiidae (and other percomorphs).

**Palatine**. The palatine is a single bone without a clear distinction between dermal and autopalatine. The palatine is mostly a laminar bone, except for the anterior region, which is more robust (Figures 5C and 6B–D). The anterior tip of the palatine forms an acute process, the maxillary process, that projects anterolaterally over the anterolateral process of the maxilla. The anterodorsal edge of the palatine has a triangular process, the ethmoid process, that articulates with the ventral surface of the lateral ethmoid. The central region of the dorsal margin of the palatine projects slightly posterodorsally, where it is continuous with the remnants of the embryonic palatoquadrate cartilage. From the insertion with the palatoquadrate cartilage, the dorsal margin projects posteroventrally forming a long acute posterior edge that reaches the coronoid process of the dentary (Figure 5C,D).

**Ectopterygoid**. The ectopterygoid is a trapezoidal, laterally compressed, laminar bone; its anterior edge is angular, projecting anterodorsally adjacent to the posterior edge of the palatine, reaching the central projection in the dorsal margin of the palatine (Figure 5C,D). The posterior margin of the ectopterygoid is straight and contacts the quadrate by juxtaposition (e.g., *Eviota sparsa*) or frontally (e.g., *Sueviota lachneri; E*. *cometa*; *E. atauroensis*, Figure 6A).

**Quadrate**. The quadrate has a convex articular head ventrally for contact with the anguloarticular. The main body of the quadrate is robust, its depth extending dorsal to the posterodorsal edge of the ectopterygoid, with a cartilaginous site that articulates with the metapterygoid (Figures 5C,D; and 6A–E). The anterior part of the quadrate is variable across species: in *Eviota sparsa* and *E. atauroensis*, the anterior margin is entirely convex, whereas in *E. cometa* and *E. bifasciata* the anterior margin has a shallow concavity where articulates with the ectopterygoid. In one specimen of *E. sparsa* (ANSP 148485), the anterior margin is angular, forming a triangular outline that articulates with the ectopterygoid. The dorsal surface of the quadrate has a deep notch dorsally for insertion of the sympletic that extends to the ventral third of the quadrate (Figure 5D). The posteroventral process of the quadrate (*sensu* Arratia and Schultze 1991) is robust, longer in length than the main body of the quadrate. The posterior edge of the posteroventral process of the quadrate is attached to the medial surface of the anterior edge of the preopercle (Figure 5D).

**Metapterygoid**. The metapterygoid is a relatively deep bone, its depth being slightly longer than the depth of the quadrate (Figures 5C and 6A–E). The width of the metapterygoid varies across ontogeny and diversity, ranging from one‐third (e.g., *E. zebrina, E. sparsa*; Figure 5D) to one‐half of its depth (e.g., *E. cometa*). The dorsal margin of the metapterygoid is convex and contacts the medial surface of the anterior edge of the hyomandibula (Figure 5C). The anterior margin of the metapterygoid is irregular and varies highly both intra‐ and interspecifically (Figure 6). The ventral margin has a cartilaginous site that contacts the quadrate.

**Hyomandibula**. The hyomandibula is a broad and robust bone with a complex shape (Figures 5C,E–G and 6A–E). The dorsal margin has two heads with cartilage for articulation with the neurocranium: the anterior one positioned at the anterodorsal edge of the hyomandibula articulates with the sphenotic (Figure 5F,G); the posterior head (Figure 5F,G) is positioned in the middle of the dorsal margin and articulates with the pterotic (Figures 4G,H and 6A). The dorsal margin of the hyomandibula is deeply concave between the articular heads, but slightly concave posteriorly (Figure 5E–G). The posterodorsal edge of the hyomandibula is triangular and projects posteriorly to the same level of the condyle for articulation with the opercle, forming a deeply concave posterior margin. The articular condyle for the opercle is robust, positioned in the middle of the posterior margin of the hyomandibula (Figure 5C,E–G). Ventral to the articular condyle, the posterior margin of the hyomandibula projects anteroventrally, forming a cartilaginous head, which marks the ventral edge of the hyomandibula (Figure 5E,F). The anterior margin of the hyomandibula is broadly convex in its dorsal region, which contacts the dorsal region of the metapterygoid. From the contact with the metapterygoid, the anterior margin of the hyomandibula projects posteroventrally to a cartilaginous head, the anteroventral process of the hyomandibula that articulates with the sympletic (Figure 5C,E–G). The lateral surface of the hyomandibula has two ridges for muscular attachment. A longitudinal ridge is present in the middle of the lateral surface for attachment of the insertion of the *levator arcus palatine* muscle (Figures 5F and 6A–E). A second ridge extends posteroventrally from the level of the opening for the hyomandibular branch of the *fascialis* nerve (VII) to the base of the opercular condyle (Figures 5F and 6A–E), serving as attachment to the muscle fibers of the muscle *adductor mandibulae pars malaris* (Datovo and Vari 2013).

**Sympletic**. The sympletic is elongated and laterally compressed. The dorsal edge is 1.5–2 times wider than the ventral region of the sympletic and articulates with the anteroventral process of hyomandibula (Figures 5C and 6A–E). The anterior margin is straight, whereas the posterior margin is slightly concave dorsally. The ventral edge of the sympletic inserts in the notch of the quadrate. The only variation observed in the sympletic was observed in *Eviota* cf. *distigma* (MCZ 13184). This specimen possesses a sympletic that, anteriorly, has the same outline of the sympletic observed in other species, however, centrally, this bone has a groove from where a rectangular lamina expands posteriorly, closing the corresponding region of the anterior region of the non‐osseus area (Figure 6E) between the preopercle and the sympletic observed in other species of *Eviota* and *Sueviota* (and other gobies as well; see Gosline 1955; Tornabene et al. 2018). The other specimen of *Eviota distigma* (UW uncat DIS1) has a sympletic similar to those observed in other species and has a regular non‐osseous gap in the suspensorium. No other dwarfgoby (or gobiid specimen) examined in this study has an enclosed suspensorium gap. Based on the material examined, we tentatively interpret this variation in the shape of the sympletic in *Eviota* cf. *distigma* (MCZ 13184) as an anomaly.

**Infraorbital one**. Infraorbital one bone is trapezoidal, laterally compressed, with a shallow cup‐like dorsal edge that articulates with the lateral ethmoid (Figures 5A,B and 6A–C). No other bones from the infraorbital series are present in *Eviota* or *Sueviota*. In *Eviota saipanensis* and *E. smaragdus*, the posterior edge of the infraorbital one is rounded and the depth of the posterior edge is higher than the depth of the anterior edge, with a distinct groove for the passage of the infraorbital canal on its lateral surface.

### 3.3 Opercular Series

**Opercle**. The opercle is laminar, laterally compressed, and triangular‐shaped (Figures 5C,E and 6A–D,F). The cup for articulation with the hyomandibula sits in the middle of the anterior margin. Ventral to the articular cup, the anterior margin is slightly convex and projects ventrally to the attachment with the subopercle. The ventral edge of the opercle is broad, continuous with a shallow concave posterior margin, which is tightly attached to the dorsal margin of the subopercle. On the posterior margin of the opercle, close to the posterodorsal edge, two thin filaments project posteriorly (Figures 5E and 6). The dorsal margin of the opercle is slightly convex. The posterodorsal edge of the opercle is broad and usually rounded in most examined species (e.g., *Eviota sparsa*, *E. cometa*, *E. bifasciata)*, but *Eviota atauroensis* has a sharp and angular posterodorsal edge of the opercle.

**Figure 7:**
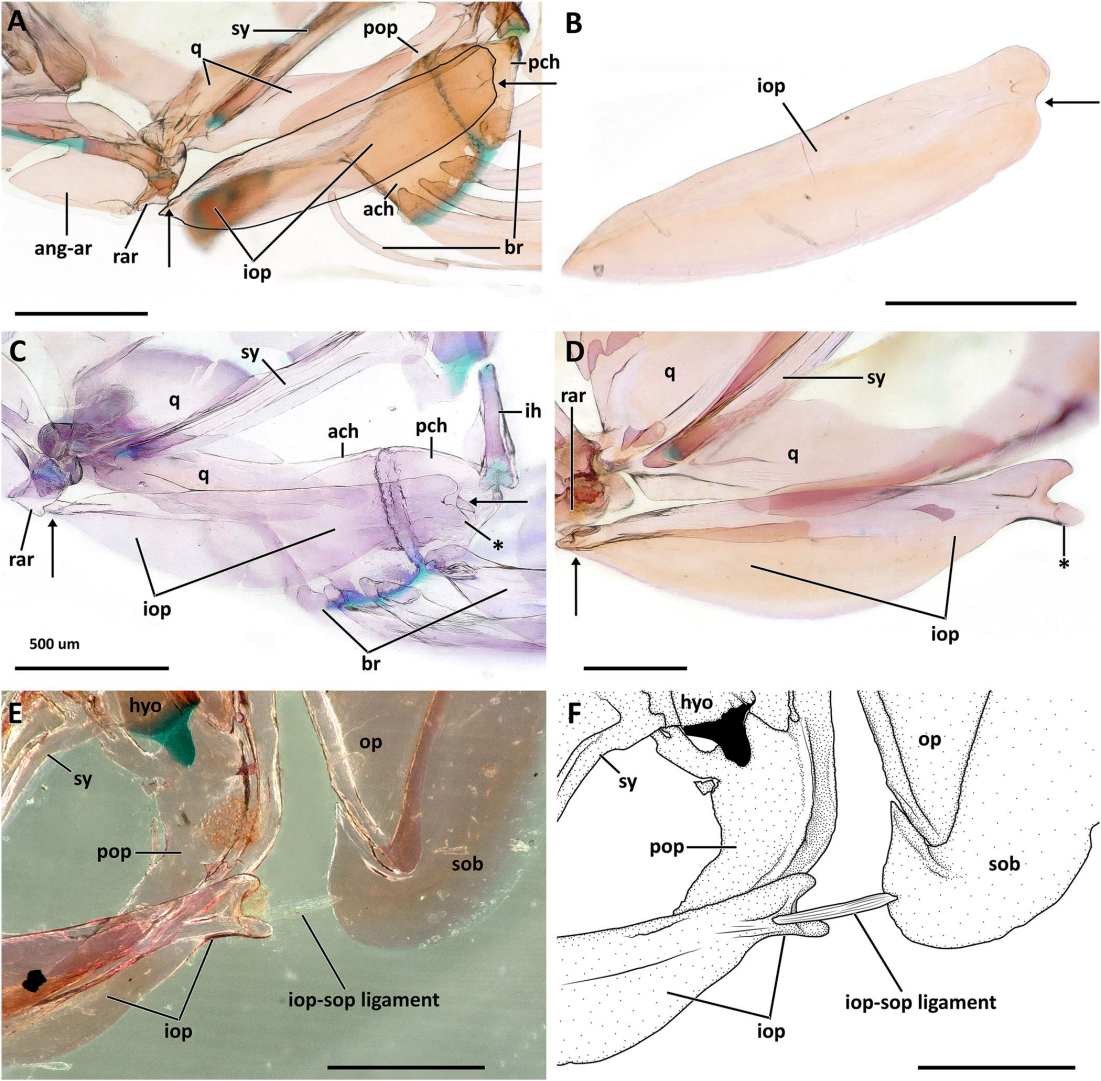
Morphological details and variation of the interopercle in *Eviota* and *Sueviota*. (A, B) Interopercular arrangement and shape in species of *Eviota* with unbranched pectoral fin rays (unbranched clade *sensu* Tornabene, Ahmadia, et al. 2013; Tornabene, Chen, and Pezold 2013), represented by *Eviota zebrina*. (A) Interaction of the interopercle with the lower jaws and the hyoid arch in ANSP 138917 (14 mm SL). The interopercle was delineated with a black line to highlight its shape. Black arrows indicate the direct contact between the interopercle and retroarticular (anterior) and ceratohyal (posterior). (B) Isolated interopercle of ANSP 138917 (14.9 mm SL). Black arrow indicates the concave posteroventral margin. (C, D) Interopercular arrangement and shape in species of *Sueviota* and *Eviota* with branched pectoral fin rays (branched clade *sensu* Tornabene, Ahmadia, et al. 2013; Tornabene, Chen, and Pezold 2013). Asterisks indicate the extended posteroventral projection forming the wrench (spanner)‐shaped posterior end of the interopercle. (C) Interaction of the interopercle with the lower jaws and the hyoid arch in ANSP 148481 (17.7 mm SL). Black arrows indicate the direct contact between the interopercle and retroarticular (anterior) and ceratohyal (posterior). (D) Details of the interopercle of (ANSP148485, 16.1 mm SL) in higher magnification. (E, F) Ethanol‐immersed image (E) and illustration (F) of the interopercular region in posteromedial view of ANSP 148485 (16.1 mm SL) highlighting the single remaining ligament (interopercle‐subopercle ligament) attached to the interopercle in species of *Eviota* and *Sueviota*. In all images, anterior to left. Scale bar, 500 µm. ach, anterior ceratohyal; ang‐ar, anguloarticular; br, branchiostegal rays; ih, interhyal; iop, interopercle; pch, posterior ceratohyal; pop, preopercle; q, quadrate; rar, retroarticular; sy, sympletic.

**Subopercle**. The subopercle is comma‐shaped, with its dorsal margin having a deep notch for contact with the V‐shaped ventral edge of the opercle (Figures 5C,E and 6A–D). The posterior margin of the subopercle is convex and continuous in all species of *Eviota* and *Sueviota* (Figures 5E and 6A–D). The only exception is *Sueviota tubicola* that has a serrated posterior margin of the subopercle (Figure 6F). The insertion of the interopercular‐subopercular ligament attaches to the anteromedial surface of the subopercle (Figure 7E,F).

**Preopercle**. The preopercle is a laminar dermal bone that bears a groove on its posterior surface for the preopercular canal for the lateralis system of canals (Figures 5C,E and 6A–E). The preopercle is laterally compressed and curved posterodorsally into an l‐shaped bone. The anterior edge of the preopercle is acute and forms a juxtaposing articulation with the lateral surface of posteroventral process of the quadrate. The posterodorsal edge of the preopercle is as wide as the hyomandibula. The dorsal margin has a deep indentation on its anterior half, forming a thin, rectangular anterodorsal edge (Figures 5C,E; 6A–D; and 7E,F). The only variation observed in the dorsal margin of the preopercle was observed in a single specimen of *Eviota* cf. *distigma* (MCZ13184; Figure 6E), in which the dorsal margin is straight and posterodorsally oriented. This arrangement expanded the anterior extent of the preopercle resulting in closure of the non‐osseus area observed in other species of *Eviota* and *Sueviota* (Figures 5C and 6A–D), as well as other gobies (Gosline 1955; Tornabene et al. 2018). The other specimen of *Eviota distigma* (CAS 248648) has the preopercle shaped similarly to the other examined specimens and has a regular non‐osseous gap in the suspensorium. No other dwarfgoby (or gobiid specimen) examined in this study has an enclosed suspensorium gap. Based on the material examined, we tentatively interpret this variation in the preopercle of specimen of *Eviota* cf *distigma* (MCZ 13184) as an anomaly.

The posterior margin of the preopercle has a groove for passage of the preoperculomandibular canal. Species that bear a pair of preopercular pores of the cephalic pore system have the groove with a pair of widened circular regions that surround the preopercular pores (Figures 5E and 6A,C,E). In *Eviota bifasciata*, the groove for the preoperculomandibular canal is relatively wider than observed in other species and has a pair of foramina inside it (Figure 6C), one for each preopercular pore. In species that lack preopercular pores, such as *Eviota sparsa* and *Sueviota aprica*, the groove is thin and continuous (Figures 5C and 7E,F).

**Interopercle**. The interopercle is a strut‐like bone with a thin ventral laminar flange. The anterior edge is angular and contacts the retroarticular directly (Figures 5C,D and 7A,C,D). The retroarticular‐interopercle ligament is absent. These characteristics occur in all examined species of *Eviota* and *Sueviota*. The posterior edge of the interopercle is present in two distinct conditions: in species of *Eviota* with unbranched pectoral‐fin rays (e.g., *E. cometa*; *E. zebrina*; *E. bifasciata*), the posterior edge is rounded and has a posteroventral concavity for articulating with the posterior ceratohyal and for attachment of the interopercular‐subopercular ligament (Figure 7A, B). In species of *Eviota* with branched pectoral‐fin rays and representatives of *Sueviota*, the posterior edge of the interopercle is conical and with a blunt edge. The ventral margin of the posterior edge of the interopercle has a deep posteroventral concavity and an additional posteroventral conical process, forming a wrench‐shaped edge that articulates with a distinct lateral process on the posterior edge of the ceratohyal (Figures 5D,E and 7C,D). The interopercular‐subopercular ligament attaches to the posteroventral process of the interopercle (Figure 7E,F). All examined species of *Eviota* and *Sueviota* lack the interopercular‐ceratohyal ligament.

### 3.4 Ventral Hyoid Arch

**Figure 8:**
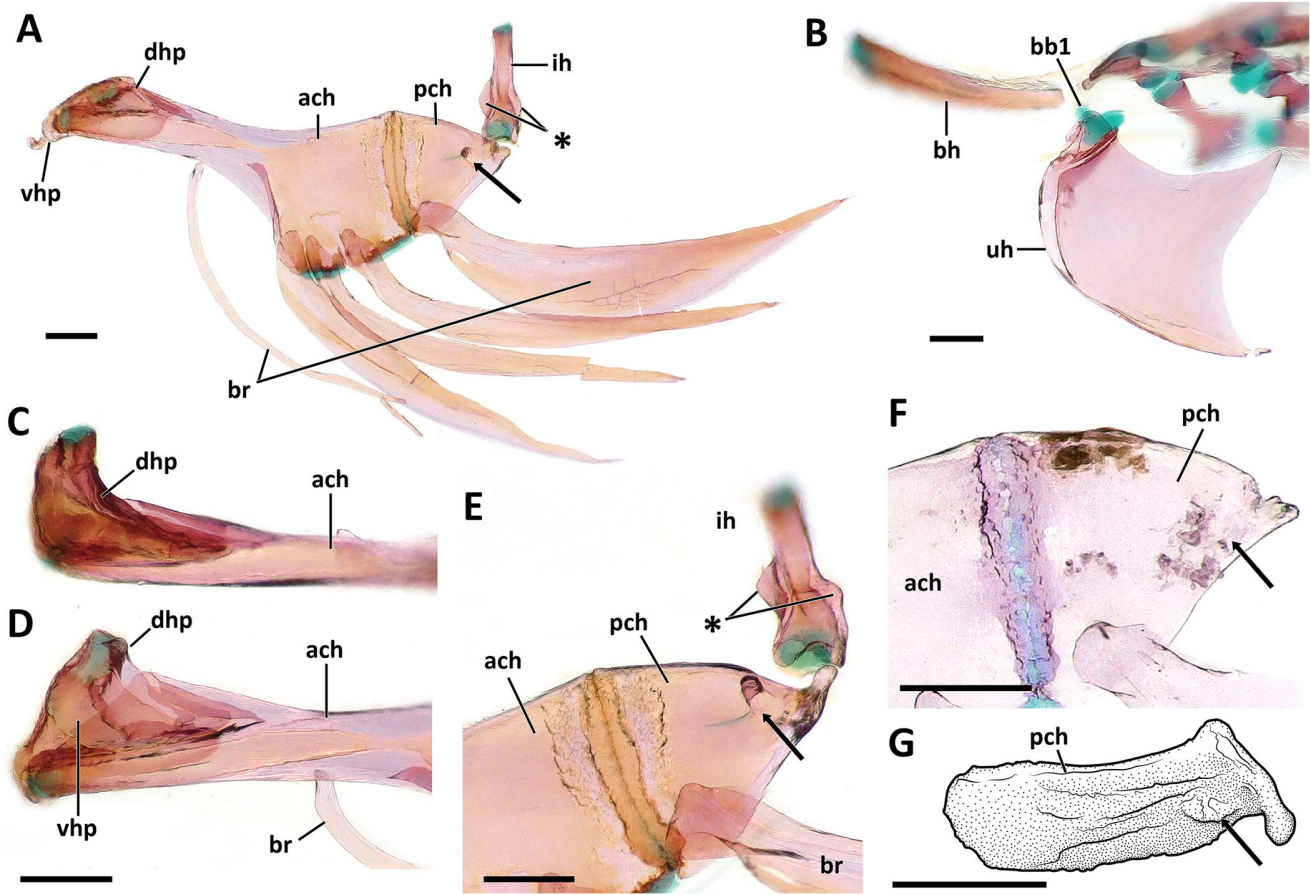
Morphology and variation of the ventral hyoid arch of *Eviota* and *Sueviota*, represented by *Eviota sparsa* (ANSP 148485, 16.1 mm SL; A–E) and *Eviota cometa* (MCZ 162965, 13 mm SL; F, G). (A) Dorsal hypohyal, ventral hypohyal, anterior ceratohyal, posterior ceratohyal, interhyal, and branchiostegal rays of *Eviota sparsa* (ANSP 148485, 16.1 mm SL) in lateral view. Black arrow indicates the anterior lateral process of the posterior ceratohyal for articulation with the interopercle. Asterisk indicates the triangular process of the interhyal for articulation with the preopercle. (B) Lateral view of the urohyal and basihyal in *Eviota sparsa* (ANSP 148485, 16.1 mm SL). (C, D) High magnification of hypohyals and anterior region of the anterior ceratohyal in dorsal (C) and medial (D) views. (E) Posterior ceratohyal and articulation with interhyal in ventro‐lateral view of species of *Sueviota* and *Eviota* with branched pectoral fin rays. Black arrow indicates the anterior lateral process of the posterior ceratohyal for articulation with the interopercle. Asterisk indicates the triangular process of the interhyal for articulation with the preopercle. (F G) Morphological details of the posterior ceratohyal of species of *Eviota* with unbranched pectoral fin rays, in lateral (F) and dorso‐lateral (G) views. Black arrow indicates the bulge for articulation with the interopercle. Scale bar, 200 µm. Anterior to left. ach, anterior ceratohyal; bh, basihyal; br, branchiostegal rays; dhp, dorsal hypohyal; ih, interhyal; pch, posterior ceratohyal; uh, urohyal; vhp, ventral hypohyal.

**Dorsal hypohyal**. The dorsal hypohyal is pyramidal, contacting the anterodorsal edge of the anterior ceratohyal, separated from the ventral hypohyal by a cap of cartilage (Figure 8A,C,D).

**Ventral hypohyal**. The ventral hypohyal is triangular, positioned ventral to the dorsal hypohyal, surrounding the medial surface of the anteroventral edge of the anterior ceratohyal (Figure 8A,C,D).

**Anterior ceratohyal**. The anterior ceratohyal is paddle‐shaped, with a thin, semi‐cylindrical anterior region, expanding towards the posterior half as a rectangular, laterally compressed region, which is approximately two times wider than the anterior region (Figure 8A). The anterior edge of the anterior ceratohyal has a cartilaginous head positioned ventrally that contacts with the ventral hypohyal (Figure 8D). The anterior margin projects posterodorsally to the dorsal cartilaginous head that articulates with the dorsal hypohyal (Figure 8D). The posterior margin of the anterior ceratohyal is straight and completely attaches to the anterior margin of the posterior ceratohyal without any sutures (Figure 8E).

**Posterior ceratohyal**. The posterior ceratohyal is triangular and laterally compressed (Figure 8A). The posterior edge has a small, vertically directed process for articulation with the interhyal. In the examined species of *Eviota* with branched pectoral‐fin rays, a distinct, narrow lateral process is positioned in the vertical middle of the posterior third of the posterior ceratohyal for articulation with the wrench‐shaped posterior edge of the interopercle (Figure 8E). Species with unbranched pectoral‐fin rays have, in the homologous location on the posterior ceratohyal, a slight bulge that contacts with the posteroventral concavity of the interopercle (Figure 8F,G).

**Interhyal**. The interhyal is a vertically elongated, rod‐like bone, having a depth slightly greater than the depth of the posterior ceratohyal (Figure 8A). The lateral margins have a triangular projection on each side of the interhyal for articulation with the preopercle (Figure 8E; see more details of this feature in Gill and Mooi 2012).

**Branchiostegal rays**. Five branchiostegal rays are present (Figure 8A). The anteriormost is the longest, approximately the length of anterior and posterior ceratohyal combined, and also the thinnest, a thread‐like bone positioned on the middle of the ventral margin of the anterior ceratohyal; the width of the first branchiostegal ray is less than one‐third of other branchiostegal rays. Second to fifth branchiostegal rays are scythe‐shaped, with the anterior edge having a concave surface for articulation with the ceratohyal. The fifth branchiostegal ray is the widest; its largest width is twice the width of the second branchiostegal ray. Branchiostegals two to four are positioned in the posterior region of the ventral margin of the anterior ceratohyal. Branchiostegal five is located in the anteroventral margin of the posterior ceratohyal.

**Figure 9:**
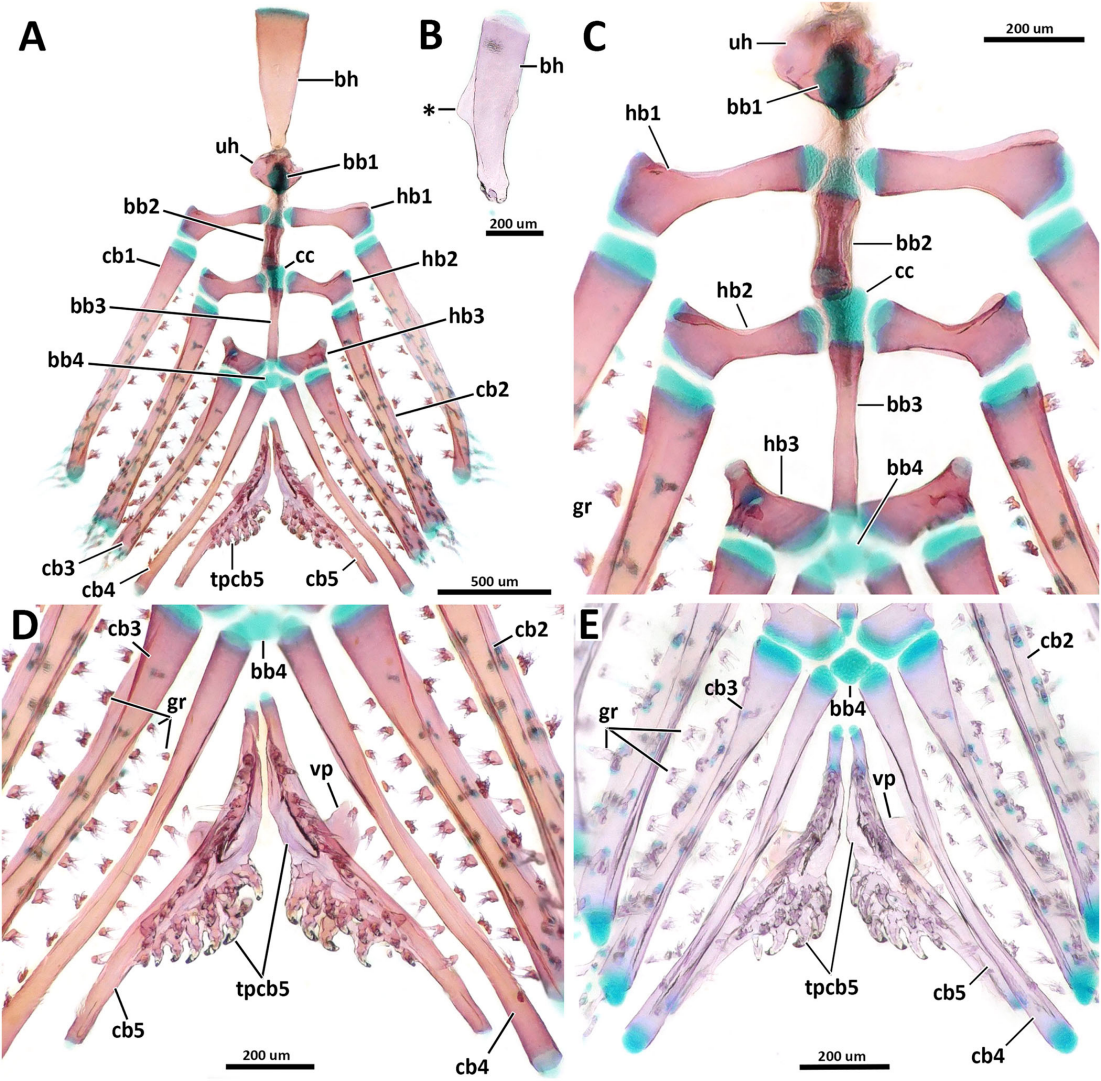
Ventral gill arches in dorsal view. (A, C, D) *Eviota sparsa* (ANSP 148485, 16.1 mm SL). (B, E) *Eviota cometa* (MCZ 162965, 13 mm SL). (A) Overview of ventral gill arches. (B) Dorsal view of the basihyal of *E. cometa*. Asterisk indicates the triangular lateral projections. (C) Higher magnification of basibranchials and hypobranchials. (D, E) Details of ceratobranchials and ventral tooth plates. Note the difference in shape of basibranchial four cartilage between *Eviota sparsa* (D) and *Eviota cometa* (E). Anterior to top. bb, basibranchial; bh, basihyal; cb, ceratobranchial; gr, gill rakers; hb, hypobranchial; tp, tooth plate; uh, urohyal; vp, ventral process of ceratobranchial five.

**Basihyal**. The basihyal is a triangular, dorsoventrally flattened bone, tapering posteriorly, the width of its anterior edge varies from two to four times the width of the posterior margin (Figures 8B and 9A). The lateral margins of the basihyal are straight in all examined specimens of *Eviota* and *Sueviota* (Figure 9A), except for *Eviota cometa*, which has a basihyal with a triangular process in the middle of each lateral margin (Figure 9B).

**Urohyal**. The urohyal is semi‐circular anteriorly, with a long conical posterior projection. (Figure 8B) The anterior margin is strongly convex, with concave dorso‐ and ventroposterior margins. Anterodorsally, a concave, cup‐shaped surface articulates with a cartilaginous basibranchial one (Figure 9C).

### 3.5 Branchial Arches

**Ceratobranchial**. Ceratobranchials one to four are rod‐shaped bones, similar in size, having a flat dorsal surface and posterior edges slightly curved dorsally for articulation with epibranchials (Figure 9A,D,E). Two longitudinal rows of gill rakers are present on the dorsal surface of ceratobranchials one to four. Rakers have a bulbous, rounded base, which bears one to three tooth‐like cusps (Figure 9C–E). Anteriomedially, ceratobranchials one to three articulate with hypobranchials one to three, respectively. The anteromedial edge of ceratobranchial four articulates with the posterolateral margins of basibranchial four cartilage. Ceratobranchial five is shorter than the other ceratobranchials, approximately three‐quarters of the length of ceratobranchial four. The ventral surface of ceratobranchial five has a trapezoidal ventromedial process projecting anteroventrally (Figure 9D,E). The medial surface of ceratobranchial five expands medially, forming the pharyngeal tooth patch of ceratobranchial five. The tooth patch and ceratobranchial five are firmly fused, not having distinct sutures among them for most parts (although the anterior edge of the tooth patch seems distinct from ceratobranchial five in *Eviota cometa*, it is not distinct posteriorly). Two groups of teeth are present on the tooth plate. A single longitudinal row with 10+ teeth on the anterodorsal margin of the tooth patch of ceratobranchial five; another cluster having two longitudinal rows with five teeth each positioned on the posteromedial expansion of the tooth patch (Figure 9D,E).

**Hypobranchial**. Three pairs of hypobranchials are present in *Eviota* and *Sueviota*. Hypobranchials are dorsoventrally flattened, transversely oriented, and medially contacting the basibranchial series (Figure 9A,B). Hypobranchials rectangular medially and expanded laterally, having a distinct anteriorly directed process on their anterolateral edge and a cartilaginous posterolateral edge that articulates with ceratobranchials one to three. The prominence of the anterolateral process of the hypobranchials increases posteriorly, with the process on hypobranchial three being as long as the length of that hypobranchial, capped by a head of cartilage.

**Basibranchial**. Basibranchial one remains cartilaginous, a distinct pyramidal cartilage, positioned medially on the ventral region of the gill basket, sitting on top of the dorsal articular surface of the urohyal (Figure 9A,C). Basibranchial two and three are rod‐shaped bones, still connected by remnants of the *copula communis* cartilage (Figure 9C). Basibranchial two is located between hypobranchials one and two. Basibranchial three is approximately 1.5 times longer than basibranchial two and is positioned between hypobranchials two and three. Basibranchial four remains cartilaginous ( = posterior copula) and is somewhat rectangular in *Eviota sparsa* (Figure 9D), but diamond‐shaped in *E. cometa* (Figure 9E). The anterolateral edges of basibranchial four contact the posteromedial edges of hypobranchial three and posterolateral edges articulate with the medial edge of ceratobranchials four.

**Figure 10:**
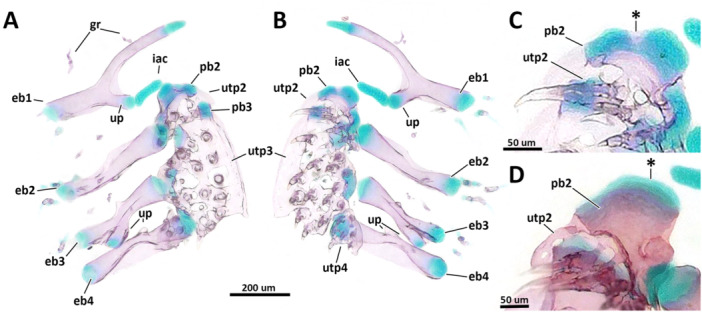
Dorsal gill arches. (A–C) *Eviota cometa* (MCZ 162965, 13 mm SL). (D) *Eviota sparsa* (ANSP 148485, 16.1 mm SL). (A, B) Dorsal (A) and ventral (B) views of dorsal gill arches of *Eviota cometa* (MCZ 162965, 13 mm SL). (C) Higher magnification of the ventral view of pharyngobranchial two of *Eviota cometa*. Asterisk indicates an anterior margin with two separate cartilaginous caps. (D) Higher magnification of the ventral view of pharyngobranchial two of *Eviota sparsa* (ANSP 148485, 16.1 mm SL). Asterisk indicates an anterior margin with a continuous cartilaginous cap. Anterior to top. eb, epibranchial; gr, gill raker; iac, interarcual cartilage; pb, pharyngobranchial; up, uncinated process; utp, upper tooth plate.

**Pharyngobranchial**. The pharyngobranchial series comprises only pharyngobranchials two and three. Pharyngobranchial two trapezoidal‐shaped, with a cartilaginous anterior edge that contacts the interarcual cartilage (Figure 10A,B). Whereas the cartilaginous anterior margin is continuous in *Eviota sparsa* (Figure 10D), E *. cometa* has two cartilaginous caps on the anterior edge, with the anterolateral cap serving for articulation with the interarcual cartilage (Figure 10C). The lateral edge of pharyngobranchial two has another cartilaginous cap for articulation with epibranchial two (Figure 10B). Pharyngobranchial three is large, approximately three times longer and 1.5 times wider than pharyngobranchial two. The anterior margin of pharyngobranchial three contacts pharyngobranchial two through a cartilaginous cap (Figure 10B).

The lateral margin of pharyngobranchial three is concave anteriorly. In the middle of the lateral margin sits a head of cartilage for articulation with epibranchial three (Figure 10A,B). On the posterolateral edge of pharyngobranchial three, there is another cap of cartilage for articulation with epibranchial four. The medial margin of pharyngobranchial three is convex (Figure 10A,B). Upper tooth patches two and three are fused to pharyngobranchials two and three. Upper tooth patch two has one to two horizontal tooth rows, and upper tooth patch three has five to six horizontal tooth rows, with three to four teeth on each row. A distinct, triangular upper tooth plate four is present ventral to the medial edge of epibranchial four, bearing three horizontal tooth rows (Figure 10B).

**Epibranchial**. Epibranchials one to four are rod‐shaped bones, transversely positioned in the dorsolateral region of the gill basket (Figure 10A,B). Epibranchial one is the longest, twice the length of other epibranchials. Its lateral region is as thick as the other epibranchials, but its anterior region is half the width of the posterior, projecting slightly ventromedially, and extending medially to the same level as the pharyngobranchials. In the middle of the dorsal margin of epibranchial one, a uncinate process is present, dorsomedially oriented, with a cartilaginous cap at its edge that articulates with the interarcual cartilage (Figure 10A,B). The interarcual cartilage is a small cartilaginous rod, approximately 1.5 times longer than the uncinate process of epibranchial one, medially contacting the anterolateral edge of pharyngobranchial two. Epibranchial two has two small, triangular processes, one on the anterior margin, the other on the posterior (Figure 10A). Epibranchial three has a small triangular process in the middle of the anterior margin, but a robust, triangular uncinate process in the posterior margin, bearing a cartilaginous cap in its posterodorsal edge that contacts epibranchial four (Figure 10A). Epibranchial four of *Eviota cometa* show a distinct uncinate process in its anterodorsal surface (Figure 10A,B). In *Eviota sparsa*, this process is incipient.

### 3.6 Vertebral Column and Intermuscular Bones

The total number of vertebrae varies from 25 to 27. Species of *Eviota* with unbranched pectoral fin rays were all observed having 25 vertebrae, with 10 abdominal ( = precaudal of Birdsong 1975) and 15 caudal vertebrae (14 preural and one ural; Tables 2, 3, 4, 5). The only exception is *E. atriventris* for having the highest counts of abdominal vertebrae (12) in all examined specimens, and 13 caudal vertebrae counts, the lowest in all examined specimens.

**Table 2:** Morphological and meristic characters of species of the branched clade of Eviota.

Species	*E. sparsa*	*E. jewettae*	*E. zonura*	*E. korechika*	*E. taeiae*	*E. melasma*	*E. erdmani*	*E. monostigma*	*E. fallax*	*E. atauroensis*	*E. latifasciata*	*E. occasa*
Number of specimens	n = 10	n = 4	n = 2	n = 1	n = 1	n = 1	n = 1	n = 1	n = 1	n = 1	n = 2	n = 1
Size range (SL, mm)	12.3‐17.0	9.2‐11.5	11.7‐16.5	19.7	13.1	15.3	12.6	19.5	14.9	16.2	14.6‐14.8	10.6
Mesethmoid, posterior portion	ossified	ossified	ossified	ossified	ossified	ossified	ossified	ossified	ossified	ossified	ossified	ossified
Interopercle, anterior edge, contact with retroarticular	direct contact	direct contact	direct contact	direct contact	direct contact	direct contact	direct contact	direct contact	direct contact	direct contact	direct contact	direct contact
Interopercle, posterior edge, shape	wrench‐shaped	wrench‐shaped	wrench‐shaped	wrench‐shaped	wrench‐shaped	wrench‐shaped	wrench‐shaped	wrench‐shaped	wrench‐shaped	wrench‐shaped	wrench‐shaped	wrench‐shaped
Pectoral‐fin rays	branched	branched	branched	branched	branched	branched	branched	branched	branched	branched	branched	branched
Abdominal vertebrae without innear haemal arch	9	9	9	9	9	9	9	9	9	9	9	9
Abdominal vertebrae with innear haemal arch	1	1	1	1	1	1	1	1	1	1	1	1
**Total abdominal vertebrae**	**10**	**10**	**10**	**10**	**10**	**10**	**10**	**10**	**10**	**10**	**10**	**10**
Preural caudal vertebrae	15	15	15	15	15	15	15	15	15	15	15	15
Ural vertebra	1	1	1	1	1	1	1	1	1	1	1	1
**Total caudal vertebrae**	**16**	**16**	**16**	**16**	**16**	**16**	**16**	**16**	**16**	**16**	**16**	**16**
**Total vertebrae**	**26**	**26**	**26**	**26**	**26**	**26**	**26**	**26**	**26**	**26**	**26**	**26**
2nd dorsal‐fin insertion formula	8 ‐ 1111111210 – 8 ‐ 1111121110 – 8 ‐ 1111112110 – 8 ‐ 111112110 8 ‐ 111111120	8 ‐ 111111111 – 8 ‐ 111111210 – 8 ‐ 111112110	8 ‐ 1111111210	8 ‐ 1111111210	8 ‐ 111112120	8 ‐ 1111112110	8 ‐ 111111120	8 ‐ 11111210	8 ‐ 1111112110	8 ‐ 1111111120	8 ‐ 111111120	8 ‐ 111111110
Anal‐fin insertion formula	10 ‐ 2111210 – 10 ‐ 2111120	10 ‐ 2111110 – 10 ‐ 2111120 – 10 ‐ 2111210	10 ‐ 2111120	10 ‐ 2111210	10 ‐ 2111210	10 ‐ 2111210	10 ‐ 2111120	10 ‐ 2111210	10 ‐ 2111120	10 ‐ 2111120	10 ‐ 2111120	10 ‐ 2111120

Species	*E. pseudaprica*	*E. saipanensis*	*E. fasciola*	*E. cf. hinanoe*	*E. cf. pinnochioi* (TS1)	*Eviota sp*. (OA1)	*Eviota sp*. (SU2)	*E. afelei*	*E. smaragdus*	*E. punctulata*	*E. teresae*	*E. distigma*
Number of specimens	n = 1	n = 2	n = 1	n = 1	n = 1	n = 1	n = 1	n = 2	n = 2	n = 2	n = 1	n = 1
Size range (SL, mm)	13.7	15.4‐17.5	11.0	11.8	11.7	10.6	9.9	10.8‐12.8	15.9‐20.5	16.5‐18.4	15.9	13.2
Mesethmoid, posterior portion	ossified	ossified	ossified	ossified	ossified	ossified	ossified	ossified	ossified	ossified	ossified	ossified
Interopercle, anterior edge, contact with retroarticular	direct contact	direct contact	direct contact	direct contact	direct contact	direct contact	direct contact	direct contact	direct contact	direct contact	direct contact	direct contact
Interopercle, posterior edge, shape	wrench‐shaped	wrench‐shaped	wrench‐shaped	wrench‐shaped	wrench‐shaped	wrench‐shaped	wrench‐shaped	wrench‐shaped	wrench‐shaped	wrench‐shaped	wrench‐shaped	wrench‐shaped
Pectoral‐fin rays	branched	branched	branched	branched	branched	branched	branched	branched	branched	branched	branched	branched
Abdominal vertebrae without innear haemal arch	9	9	9	9	9	9	9	8‐9	8	8	8	8
Abdominal vertebrae with innear haemal arch	1	1	1	1	1	1	1	1‐2	2	2	2	2
**Total abdominal vertebrae**	**10**	**10**	**10**	**10**	**10**	**10**	**10**	**10**	**10**	**10**	**10**	**10**
Preural caudal vertebrae	15	15	15	15	15	15	15	15	15	15	15	15
Ural vertebra	1	1	1	1	1	1	1	1	1	1	1	1
**Total caudal vertebrae**	**16**	**16**	**16**	**16**	**16**	**16**	**16**	**16**	**16**	**16**	**16**	**16**
**Total vertebrae**	**26**	**26**	**26**	**26**	**26**	**26**	**26**	**26**	**26**	**26**	**26**	**26**
2nd dorsal‐fin insertion formula	8 ‐ 111111210	8 ‐ 1111111210	8 ‐ 1111111120	8 ‐ 1111111210	8 ‐ 111111210	8 ‐ 11111210	8 ‐ 111111210	8 ‐ 1111111210	8 ‐ 1111111120	8 ‐ 1111112110 ‐ 8 ‐ 1111111210	8 ‐ 1111112110	8 ‐ 1111111210
Anal‐fin insertion formula	10 ‐ 2111120	10 ‐ 2111120	10 ‐21111210	10 ‐ 2111120	10 ‐ 2111120	10 ‐ 2111210	10 ‐ 2111120	10 ‐ 2111120	10 ‐ 21111110	10 ‐ 2111120	10 ‐ 2111110	10 ‐ 2111120

**Table 3:** Morphological and meristic characters of Eviota unbranched clade.

Species	*E. atriventris*	*E. bifasciata*	*E. infulata*	*E. lachdeberei*	*E. nigriventris*	*E. prasites*	*E. pellucida*	*E. sebreei*
Number of specimens	n = 2	n = 2	n = 1	n = 1	n = 1	n = 1	n = 2	n = 1
Size range (SL, mm)	14.7‐15.3	14‐14.5	10.7	12.2	12.4	17.8	15.3‐17.6	12.7
Mesethmoid, posterior portion	cartilaginous	cartilaginous	cartilaginous	cartilaginous	cartilaginous	cartilaginous	cartilaginous	cartilaginous
Interopercle, anterior edge, contact with retroarticular	direct contact	direct contact	direct contact	direct contact	direct contact	direct contact	direct contact	direct contact
Interopercle, posterior edge, shape	shallow notch	shallow notch	shallow notch	shallow notch	shallow notch	shallow notch	shallow notch	shallow notch
Pectoral‐fin rays	unbranched	unbranched	unbranched	unbranched	unbranched	unbranched	unbranched	unbranched
Abdominal vertebrae without innear haemal arch	11	9	9	9	9	9	9	9
Abdominal vertebrae with innear haemal arch	1	1	1	1	1	1	1	1
**Total abdominal vertebrae**	**12**	**10**	**10**	**10**	**10**	**10**	**10**	**10**
Preural caudal vertebrae	12	14	14	14	14	14	14	14
Ural vertebra	1	1	1	1	1	1	1	1
**Total caudal vertebrae**	**13**	**15**	**15**	**15**	**15**	**15**	**15**	**15**
**Total vertebrae**	**25**	**25**	**25**	**25**	**25**	**25**	**25**	**25**
2nd dorsal‐fin insertion formula	8 ‐ 111112110	8 ‐ 1111121110	8 ‐ 111111210	8 ‐ 11111211	poorly	8 ‐ 111112110	8 ‐ 111111210	8 ‐ 1111121110
Anal‐fin insertion formula	10 ‐ 211120	9 ‐ 1111210	10 ‐ 2111110	10 ‐ 211120	ossified	10 ‐ 211120	10 ‐ 211110	10 ‐ 2111120

Species	*E. cometa*	*E*. cf. *nigrispinna*	*E. rubriceps*	*E. shimadai*	*E. sigillata*	*E. storthynx*	*E. zebrina*
Number of specimens	n = 2	n = 1	n = 1	n = 1	n = 1	n = 1	n = 1
Size range (SL, mm)	13‐14.1	10.9	11.9	9.75	10.7	13.3	14.18
Mesethmoid, posterior portion	ossified	ossified	ossified	ossified	ossified	ossified	ossified
Interopercle, anterior edge, contact with retroarticular	direct contact	direct contact	direct contact	direct contact	direct contact	direct contact	direct contact
Interopercle, posterior edge, shape	shallow notch	shallow notch	shallow notch	shallow notch	shallow notch	shallow notch	shallow notch
Pectoral‐fin rays	unbranched	unbranched	unbranched	unbranched	unbranched	unbranched	unbranched
Abdominal vertebrae without innear haemal arch	8	8	8	8	8	8	8
Abdominal vertebrae with innear haemal arch	2	2	2	2	2	2	2
**Total abdominal vertebrae**	**10**	**10**	**10**	**10**	**10**	**10**	**10**
Preural caudal vertebrae	14	14	14	14	14	14	14
Ural vertebra	1	1	1	1	1	1	1
**Total caudal vertebrae**	**15**	**15**	**15**	**15**	**15**	**15**	**15**
**Total vertebrae**	**25**	**25**	**25**	**25**	**25**	**25**	**25**
2nd dorsal‐fin insertion formula	8 ‐ 111111210 ‐ 8 ‐ 11111120	8 ‐ 111111210	8 ‐ 111112110	8 ‐ 111112110	8 ‐ 111112110	8 ‐ 111112110	8 ‐ 1111112110
Anal‐fin insertion formula	10 ‐ 211120	10 ‐ 2111110	10 ‐ 211120	10 ‐ 211110	10 ‐ 2112110	10 ‐ 211110	10 ‐ 2111210

**Table 4:** Morphological and meristic characters of species of Sueviota

Species (genus *Sueviota*)	*S. lachneri*	*S. larsonae*	*S. aprica*	*S. tubicola*	*S. atrinasa*
Number of specimens	n = 5	n = 2	n = 2	n = 2	n = 2
Size range (SL, mm)	10.9‐19.1	13.2‐15.7	10.1‐11.6	12‐15.6	19‐19.5
Mesethmoid, posterior portion	ossified	ossified	ossified	ossified	ossified
Interopercle, anterior edge, contact with retroarticular	direct contact	direct contact	direct contact	direct contact	direct contact
Interopercle, posterior edge, shape	wrench‐shaped	wrench‐shaped	wrench‐shaped	wrench‐shaped	wrench‐shaped
Pectoral‐fin rays	branched	branched	branched	branched	branched
Abdominal vertebrae without innear haemal arch	9	9	9	9	9
Abdominal vertebrae with innear haemal arch	1	1	1	1	1
**Total abdominal vertebrae**	**10**	**10**	**10**	**10**	**10**
Preural caudal vertebrae	15	15	15	15	16
Ural vertebra	1	1	1	1	1
**Total caudal vertebrae**	**16**	**16**	**16**	**16**	**17**
**Total vertebrae**	**26**	**26**	**26**	**26**	**27**
2nd dorsal‐fin insertion formula	8 ‐ 1111112110 ‐ 8 ‐ 111111120	8 ‐ 11111111110 ‐ 8 ‐ 1111111120	8 ‐ 1111111120 ‐ 8 ‐ 1111112110	8 ‐ 1111111210	8 ‐ 1111112110 ‐ 8 ‐ 1111111120
Anal‐fin insertion formula	10 ‐ 2111120	10 ‐ 21111210 ‐ 10 ‐ 2111120	10 ‐ 21111210 ‐ 10 ‐ 21112110	10 ‐ 2111120	10 ‐ 2111120 ‐ 10 ‐ 21111110

**Table 5:** Morphological and meristic characters of Paragobiodon, Gobiodon, Pleurosicya, and Bryaninops

Species	*Paragobidon modestus*	*Paragobiodon echinocephalus*	*Gobiodon rivulatus*	*Gobiodon quiquestrigatus*	*Gobiodon* sp.	*Pleurosicya micheli*	*Bryaninops amplus*
Number of specimens	n = 1	n = 1	n = 1	n = 1	n = 1	n = 1	n = 1
Size range (SL, mm)	19.5	19.8	22.4	31.3	22.0	14.9	14.9
Mesethmoid, posterior portion	ossified	ossified	ossified	ossified	ossified	ossified	cartilaginous
Interopercle, anterior edge, contact with retroarticular	ligament mediated	ligament mediated	ligament mediated	ligament mediated	ligament mediated	ligament mediated	ligament mediated
Interopercle, posterior edge, shape	shallow notch	shallow notch	straight	straight	straight	shallow notch	shallow notch
Pectoral‐fin rays	branched	branched	branched	branched	branched	branched	branched
Abdominal vertebrae without innear haemal arch	7	7	7	7	7	9	9
Abdominal vertebrae with innear haemal arch	3	3	3	3	3	1	1
**Total abdominal vertebrae**	**10**	**10**	**10**	**10**	**10**	**10**	**10**
Preural caudal vertebrae	15	15	15	N/A	15	15	15
Ural vertebra	1	1	1	N/A	1	1	1
**Total caudal vertebrae**	**16**	**16**	**16**	**N/A**	**16**	**16**	**16**
**Total vertebrae**	**26**	**26**	**26**	**N/A**	**26**	**26**	**26**
2nd dorsal‐fin insertion formula	8 ‐ 11111111110	8 ‐ 11111111110	8 ‐ 11111111210	N/A	8 ‐ 11111111210	8 ‐ 1111111110	8 ‐ 111111110
Anal‐fin insertion formula	9 ‐ 1111111110	10 ‐ 21111120	10 ‐ 2111120	N/A	10 ‐ 2111120	10 ‐ 21111110	10 ‐ 21111110

**Figure 11:**
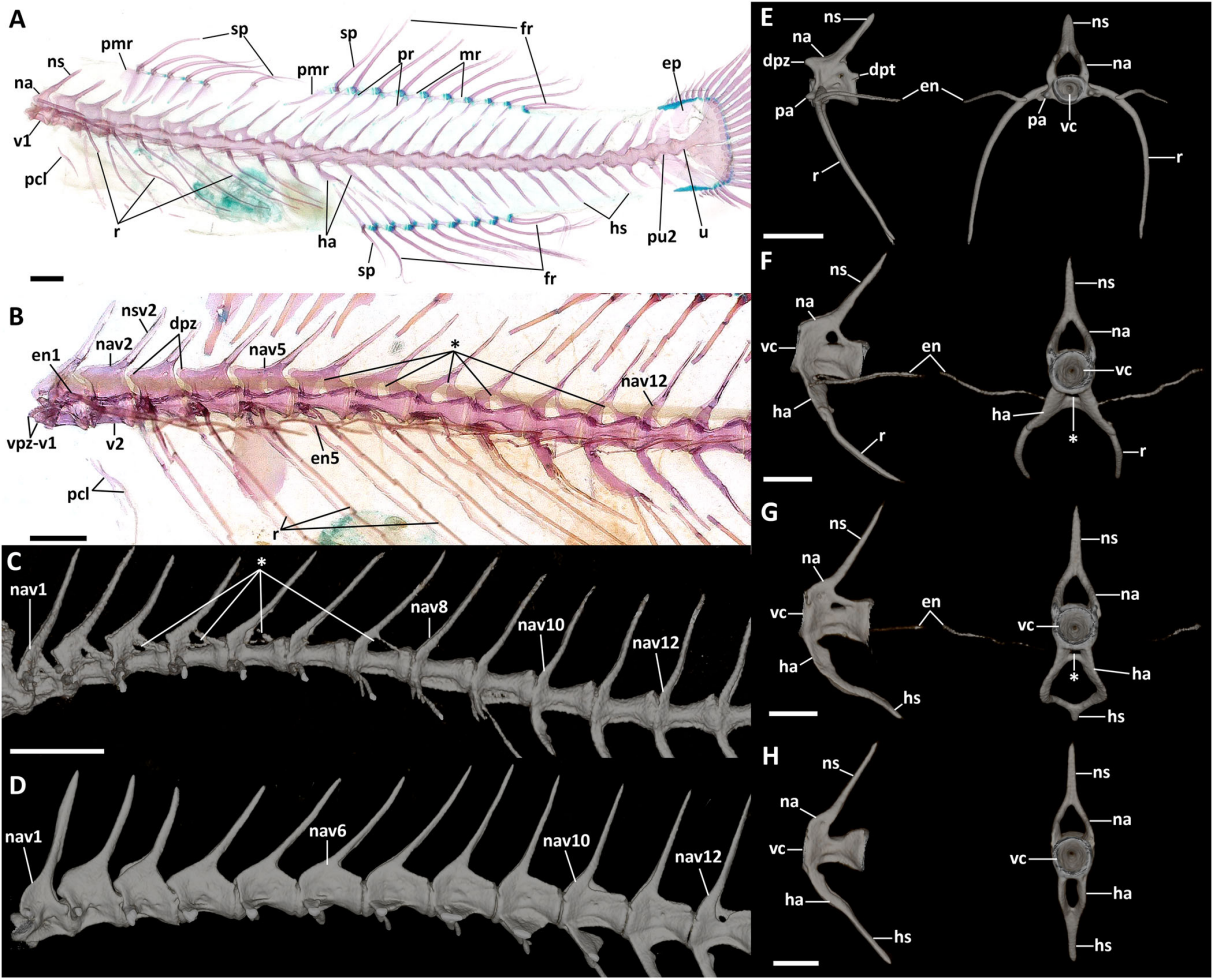
Vertebral column, intermuscular bones, and unpaired fins of *Eviota* and *Sueviota*. (A) Complete vertebral column of *Sueviota lachneri* (ROM 730CS, 16.6 mm SL). (B) Anterior vertebrae of *Eviota sparsa* (ANSP 148485, 16.1 mm SL). Note the pair of circular ventral pre‐zygapophyses on the anteroventral edge of the first vertebrae for articulation with the occipital condyles of the neurocranium. Asterisk indicates the fenestra on the lateral wall of the abdominal neural arches gradually enlarging posteriorly. (C) Anterior vertebrae of *Eviota sigillata* (CAS 248550, 10.7 mm SL). Pterygiophores, ribs, and intermuscular bones removed digitally. Asterisk indicates the fenestra on the lateral wall of the abdominal neural arches gradually enlarging posteriorly resulting in neural arches being restricted to anterior portion of the vertebral centrum from the eighth to the 20th vertebra. (D) Anterior vertebrae of *Eviota prasites* (UWFC uncatalogued EPR3, 17.8 mm SL). Pterygiophores, ribs, and intermuscular bones removed digitally. Note that the lateral walls of the neural arches from v1–v11 do not bear any openings. (E–H) individual vertebrae of *Eviota sparsa* (ANSP 151998, 17.11 mm SL, paratype). (E) Lateral (left) and anterior (right) views of the third vertebra. (F) Lateral (left) and anterior (right) views of the tenth vertebra. Asterisk indicates the enclosed parapophysis‐like haemal arch. (G) Lateral (left) and anterior (right) views of the eleventh vertebra. Asterisk indicates the enclosed base of the haemal arch for passage of blood vessels with the remaining parts of the arch enclosing ventrally with the haemal spine. (H) Lateral (left) and anterior (right) views of the twelfth vertebra. Scale bar, 500 µm. dpz, dorsal pre‐zygapophysis; dpt, dorsal post‐zygapophysis; en, epineural; ep, epural; fr, fin ray; ha, haemal arch; hs, haemal spine; mr, middle radial; na, neural arch; ns, neural spine; pcl, ventral postcleithrum; pmr, proximal‐middle radial; pr, proximal radial; pu, pre‐ural centrum; r, rib; sp, fin‐spine; u, urostyle; v, vertebral centrum; vpz, ventral pre‐zygapophysis.

Species of *Eviota* with branched pectoral fins, *Sueviota lachneri*, *Sueviota larsonae*, *Sueviota aprica*, and *Sueviota tubicola* have 26 vertebrae (Figure 11A), 10 abdominal and 16 caudal (15 preural and one ural) vertebrae. *Sueviota atrinasa* is the only examined species with 27 vertebrae. Similar to the other species of *Sueviota*, *S. atrinasa* has 10 abdominal vertebrae. The difference is in the caudal counts, having 17 caudal vertebrae (16 preural and one ural centrum).

**Vertebral centrum**. Vertebral centra are spool‐shaped laterally, with a small fenestra centrally for passage of the remnants of the constricted notochord.

**Neural arch**. Neural arches are convex, posteromedially projected, and have rectangular lateral walls (Figure 11). Depth of neural arches are approximately 1.5 times that of the corresponding centrum. Neural arches from vertebrae one to five have prominent dorsal pre‐zygapophyses (Figure 11A–E). Neural arches from vertebrae 12 to 23 are restricted to the anterior half of the dorsal surface of its respective centrum. Neural arches of preural centra two and three extend across the entire dorsal surface of its centrum. Neural arches of vertebrae 1–11 were observed with three patterns across dwarfgoby diversity regarding the extent of the lateral walls of the neural arches over the centrum, as well the presence and prominence of a fenestra in that region. The first pattern observed has the neural arches from vertebrae 1–11 extending throughout the dorsal surface of the centrum. A small, circular fenestra is observed on the neural arches of fifth centrum (Figure 11B). From vertebrae 6–11, the fenestra gradually increases in size, gradually reducing the posterior portion of the neural arches, with that region restricted to a thin wall on vertebra 11 (Figure 11B,F,G). This condition was observed in all examined species of *Sueviota*; *E. cometa, Eviota rubriceps*, *E. bifasciata*, *E. nigriventris*, *E. zebrina*, *E. storthynx*, *E. seebrei*, *E. nigrispinna* (unbranched pectoral fin rays clade); *E. sparsa*, *E. jewettae*, *E. korechika*, *E. taeiae*, *E. monostigma*, *E. fallax*, *E. punctulata*, *E. albolineata*, *E. occasa, E. pseudaprica, E. teresae*, *E. epiphanes*, *Eviota* cf. *pinocchioi*, and *Eviota* sp. (CAS 248644, CAS 248645) (branched clade).

The second condition observed has the fenestra on the lateral wall of the neural arches present already on the second vertebra (Figure 11C). Similarly, the fenestra increases in size posteriorly, with the posterior portion of the neural arches reduced to a thin bridge on vertebrae four to seven. The neural arches from vertebrae 8–11 are restricted to the anterior half of the dorsal surface of the centrum, similar to the observed in the posterior caudal caudal vertebrae (Figure 11C). This condition is present only in *Eviota sigillata* and *Eviota shimadai*.

The third condition observed has the arches extending throughout the dorsal surface of the vertebral centrum, either lacking a distinct fenestra or having a small fenestra at the base of neural arches of vertebrae 1–11 (Figure 11D). From the fifth vertebra, the depth of the posterior portion of the neural arches gradually reduce, with the depth of the posterior portion of the neural arches of vertebrae 10 and 11 being less than a quarter of the depth of the anterior margin (Figure 11D). From the twelfth vertebra, neural arches are restricted to the anterior portion of the vertebral centrum. This condition is observed in *Eviota atriventris*, *E. lachdeberei*, *E. prasites*, *E. zonura* (unbranched clade), *E. teresae*, *E. latifasciata*, *E. distigma*, *E. melasma*, *E. erdmani*, *E. atauroensis*, *E*. cf. *hinanoe, E. smaragdus*, *E. cf fasciola*, and *E. saipanensis* (branched clade).

Potential intraspecific variation among these conditions was observed only in *Eviota afelei*, wherein one specimen (UW uncat afelei20) had the first condition and the other (MCZ 13182) had the third condition. This variation, however, might indicate the existence of a species complex within specimens identified as *E. afelei*.

**Neural spine**. Neural spines are posterodorsally oriented, abdominal neural spines arranged at an angle of approximately 45 degrees; caudal neural spines positioned at a 60–70‐degree angle (Figure 11). Neural spine of the third preural centrum is the longest in the vertebral column, approximately 1.5 times longer and twice wider than the previous neural spine, having its distal tip contacting the distal caudal radial cartilage.

**Parapophysis and Haemal Arch**. Parapophyses are dorsoventrally flattened, triangular‐shaped, located in the anteroventral region of the centra. Parapophysis of centrum one is robust, dorsally flattened, and projects anteriorly to articulate with the condyles on the exoccipital. Parapophyses from centra two and three are small, less than a third of the length of a centrum, having a small anterior projection that contacts the preceding vertebral centrum (Figure 11E). Parapophyses from centra four to eight gradually increase in size and project ventrolaterally.

The first haemal arch observed in most examined species is similar to a parapophysis, shaped as two triangular projections extending antero‐laterally and supporting ribs in their ventral tips. At the base of the arches, however, there is a horizontal bar connecting each side of the arches, making an enclosed arch (Figure 11F). A single parapophysis‐like haemal arch is present on the tenth vertebra in *Sueviota lachneri*, *S. larsonae*, *S. aprica*, *S. atranasa* and *Sueviota*; *E. sparsa*, *E. jewettae, E. zonura*, *E. korechika, E. taeiae, E. melasma, E. erdmani, E. monostigma, E. fallax, E. atauroensis, E. saipanensis, E. latifasciata, E. occasa, E. pseudaprica, E*. cf. *fasciola*, *E. pinocchioi*, and *Eviota* sp. (CAS 248644, CAS 248645) (*Eviota* branched‐clade); *E. nigriventris*, *E. prasites*, *E. pellucida*, *E. seebrei*, *E. zebrina*, and *E. sigillata* (*Eviota* unbranched‐clade). *Eviota distigma* also has a single parapophysis‐like haemal arch, but it is located on vertebra 12. A second parapophysis‐like haemal arch is present on the ninth vertebra of *E. punctulata, E. teresae, E. smaragdus, E. distigma* (*Eviota* branched‐clade); *E. shimadai, E. atriventris, E. storthynx, E. lachdeberei, E. infulata, E. cf. nigrispinna*, and *E. rubriceps* (*Eviota* unbranched‐clade).

The haemal arches of eleventh vertebra are fused medially with its haemal spine (Figure 11B,G). The haemal spine of the eleventh project posteroventrally past the dorsal tip of anal‐fin pterygiophores. In all examined species, the haemal arches of the eleventh vertebra enclose the posterior wall of the abdominal cavity (The only exception is *Eviota bifasciata*, which occurs on the twelfth vertebra). This haemal arch has a horizontal bar at the base of the arches, forming a distinct cavity potentially for the passage of blood vessels (Figure 11G), whereas the remaining parts of the arch are used for attachment of hypaxial muscles. Haemal arches posterior to vertebra 11 are simple and narrow, located in the anterior third of the ventral surface of the caudal centrum (Figure 11A,H). The last three caudal vertebrae, however, have haemal arches located in the posterior third of the ventral surface of the centrum.

**Haemal Spine**. The anteriormost haemal spine is on the eleventh vertebra and is the smallest, about one‐third the depth of the first haemal arch (Figure 11G). Posterior haemal spines gradually increase in size (Figure 11A). Haemal spine of the third preural centrum is slightly longer and thicker than preceding spines, having its ventral edge contacting the ventral distal caudal cartilage (neural and haemal arches and spines on preural centrum two are described under caudal skeleton).

**Ribs**. Ribs are present from abdominal vertebra 3–10, dorsomedially sitting on the posterodorsal surface of the parapophysis or haemal arches, extending posteroventrally to surround the lateral surface of the abdominal cavity. Ribs have similar lengths, except for rib articulating with the parapophysis of centrum ten, which is one‐half the length of that on the preceding vertebra (Figure 11).

**Intermuscular bones**. A single series of intermuscular bones, the epineural series, is present. Ten to fourteen epineural bones are present throughout the abdominal and the anterior region of the caudal region. Epineurals are rod‐shaped bones, posterolaterally oriented, and approximately half the length of ribs. Epineural one and epineural two have their anteromedial tip contacting the neural arches of vertebral centra one and two, respectively. All other epineurals have their anteromedial tips sitting on the ventral region of the parapophysis, close to where parapophyses articulate with ribs (Figure 11B,E–G).

### 3.7 Dorsal Fins

Species of *Eviota* and *Sueviota* have six spines in their first dorsal fin and a second dorsal fin containing one spine and seven to ten soft fin rays, with the last ray often branched to the base (Lachner and Karnella 1978, 1980; Jewett and Lachner 1983; Winterbottom and Hoese 1988; Greenfield and Winterbottom 2016; Tornabene et al. 2021).

**Figure 12:**
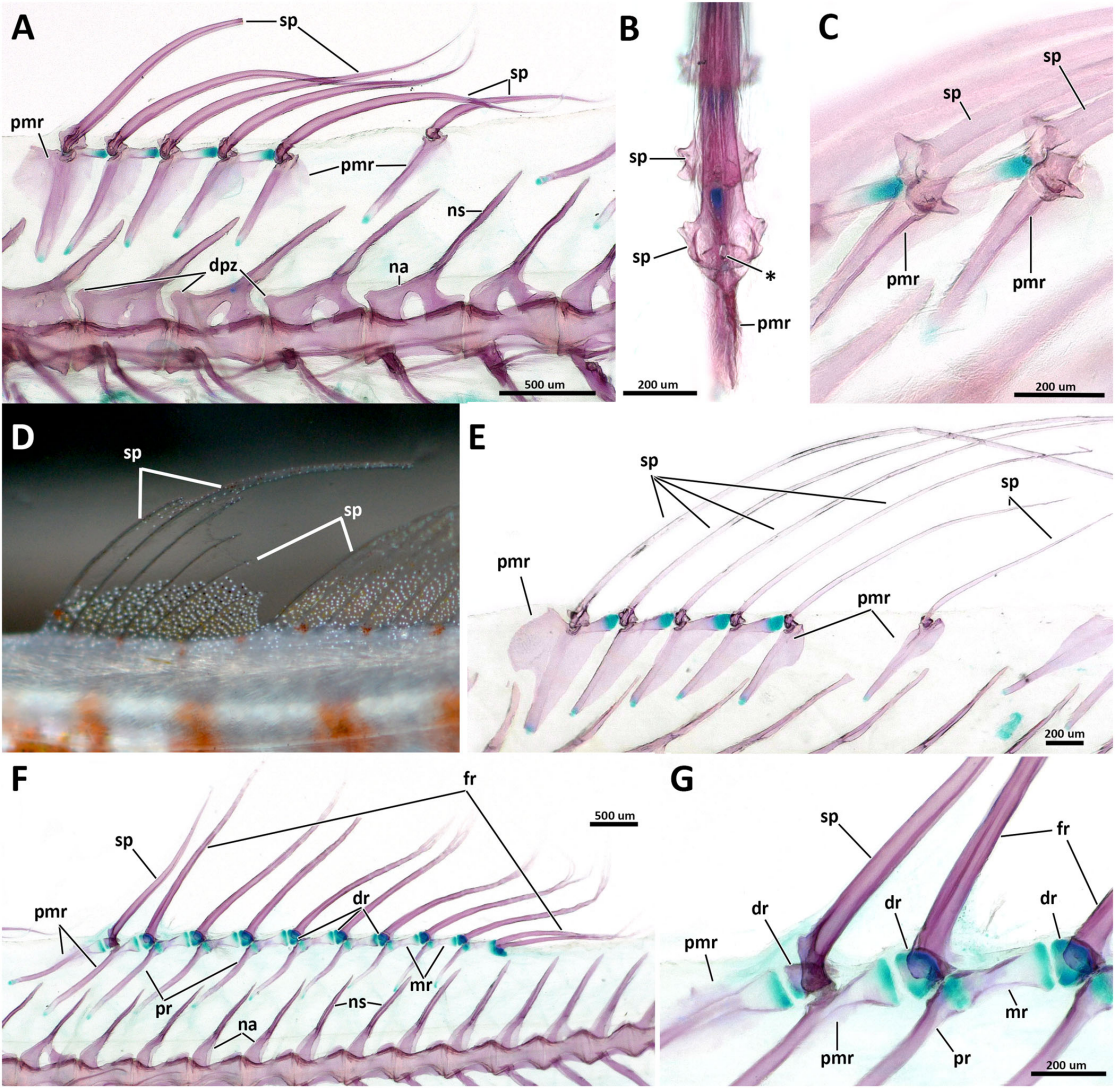
Dorsal fins of *Eviota* and *Sueviota*. (A) First (spinous) dorsal fin of *Sueviota lachneri* (ROM 730CS, 16.6 mm SL) in lateral view. (B, C) Morphological details of the articulation between fin spines and pterygiophores in *Sueviota lachneri* (ROM 730CS, 16.6 mm SL). Dorsal view of first dorsal fin spine (B) and dorso‐lateral view of fourth and fifth sin spines (C). Asterisk indicates the opening on the base of the spine for articulation with the proximal‐middle radial. (D) Spinous (first) dorsal fin of *Eviota shimadai* (MCZ 175066). Note the single filamentous spine. (E) Spinous dorsal fin of *Eviota cometa* (MCZ 162965, 13 mm SL) with four filamentous spines. (F) Second dorsal fin (soft) of *Sueviota lachneri* (ROM 730CS, 16.6 mm SL). (G) High magnification of the anterior pterygiophores of the second dorsal fin highlighting the ossified distal radial. dpz, dorsal pre‐zygapophysis; dr, distal radial; fr, fin ray; mr, middle radial; pmr, proximal‐middle radial; pr, proximal radial; na, neural arch; ns, neural spine; sp, fin‐spine.

**First dorsal‐fin pterygiophores**. The five anterior pterygiophores of the first dorsal fin are close to each other, having their distal edges contacting the subsequent spine (Figure 11A and 12A,E). The sixth pterygiophore does not contact the preceding proximal‐middle radial bone, although its spines are still connected through the fin membrane. The first two anterior pterygiophores insert in the third interneural space (between neural spines of vertebra three and four) and pterygiophores three and four insert in the fourth interneural space. The fifth pterygiophore inserts on the fifth interneural space and the sixth pterygiophore on the sixth interneural space. No elements are present on the seventh interneural space (formula 3‐22110). The first proximal‐middle radial is the largest, with a large, convex, anteriorly projecting margin that extends from the base of the spine to the ventral region of the proximal‐middle radial. The posterior margin also projects posteriorly. Proximal‐middle radials two to six are triangular, having only a posteriorly projected posterior margin. The anterior margin is straight, without projections, having the spine sitting on its anterodorsal edge. Separate and distinct distal radials were not observed.

**Dorsal‐fin spines**. Dorsal spines are unsegmented, single, bilaterally fused bones (Figure 12A,E). The bases have two pairs of dorsolaterally oriented processes, one pair on the edges of the anterior margin of the spine, the other pair on the edges of the posterior margin (Figure 11B). The first and the sixth dorsal fin spines are supernumerary elements. The anterior region of the base of the first spine is pierced for articulation with the proximal‐middle radial bone (Figure 12B). The posterior region of the base of the spines expands posteriorly onto the next proximal‐middle radial, similar to spinous distal radials of other acanthomorphs (Figure 12B,C; see further in Baldwin and Johnson 1993; Hilton 2011), although no sutures were observed. Determination of the homology of this posterior extension of the dorsal spines requires further investigation. In *Eviota* and *Sueviota*, spines sit directly into proximal‐middle radial bones, without distal radials.

The length of spines across *Eviota* and *Sueviota* is highly variable, from being relatively short, with the anterior spines extending posteriorly only to the interdorsal space, to very long and filamentous, with a spine extending posteriorly to the insertion of the second dorsal fin (Figure 12A,D,E); see also Lachner and Karnella 1978, 1980; Jewett and Lachner 1983; Winterbottom and Hoese 1988; Gill and Jewett 2004; Greenfield and Winterbottom 2016). The presence of long, filamentous spines also varies intraspecifically. In *E. japonica*, for example, filamentous spines occur in both males and females, whereas in other species, such as *E. hoesei* and *Sueviota atrinasa*, filamentous spines may occur only in males. *S. lachneri*, conversely, have both males and females with short spines.

**Second dorsal‐fin pterygiophores**. The second dorsal fin has its first pterygiophore supporting a fin spine and inserts in the eighth interneural space (Figure 12A). The first pterygiophore (proximal‐middle radial) is thinner than the proximal‐middle radial bones of the first dorsal fin. Ventrally, the first proximal‐middle radial of the second dorsal fin is elongated, rod‐shaped, with dorsal edge width twice the width of its ventral edge. The dorsal region of the first proximal‐middle radial of the second dorsal fin has narrow convex projections on both anterior and posterior margins (Figure 12F). A distinct, ossified distal radial bone is present, articulating with the base of the spine (Figure 12G).

The second pterygiophore of the second dorsal fin supports fin rays, but is still formed by a single proximal‐middle radial, inserting on the ninth interneural space. Its ventral part is elongate, rod‐like in shape, with its dorsal edge projecting and widening posteriorly into a hockey‐stick shaped bone. The anterodorsal margin of the second pterygiophore of the second dorsal fin also supports the fin spine of the second dorsal fin (Figure 12G), a condition observed across the diversity of gobies (Birdsong et al. 1988). All other pterygiophores of the second dorsal fin have separated proximal and middle radial bones. Second dorsal‐fin pterygiophores three, four, and five insert on interneural spaces ten, eleven, and twelve. Insertion of the remaining pterygiophores usually has one pair of pterygiophores inserting into a single interneural space (exceptions only in one specimen of *Eviota jewettae* [ANSP 150918] and another of *Sueviota larsonae* [ROM 41409]). Which interneural space has a pair of pterygiophores varies intraspecifically and across the diversity of dwarfgobies (Table 2, 3, 4, 5). The last pterygiophore of the second dorsal fin supports two soft rays.

The proximal radials are elongate bones, with a slightly widened dorsal edges, similar in length to the first pterygiophore of the second dorsal fin. The middle radials are spool‐shaped, longitudinally oriented, their length one‐third of the length of the proximal radial. Distal radials are present in all pterygiophores of the second dorsal fin except for the posteriormost, where fin rays articulate directly with the middle radial. The shape of distal radials varies from circular to trapezoidal. Composition of distal radials is also variable: in *Eviota sparsa*, all distal radials of the second dorsal fin supporting fin rays are cartilaginous. In *Sueviota lachneri*, distal radials from pterygiophores two to five have their middle with an ossified core (Figure 12F,G). In *Eviota cometa* and *E. latifasciata*, all distal radials of the second dorsal fin have an ossified core (extent of these observations are limited to cleared‐and‐stained specimens; not visible in micro CT‐scans).

### 3.8 Anal Fin

**Figure 13:**
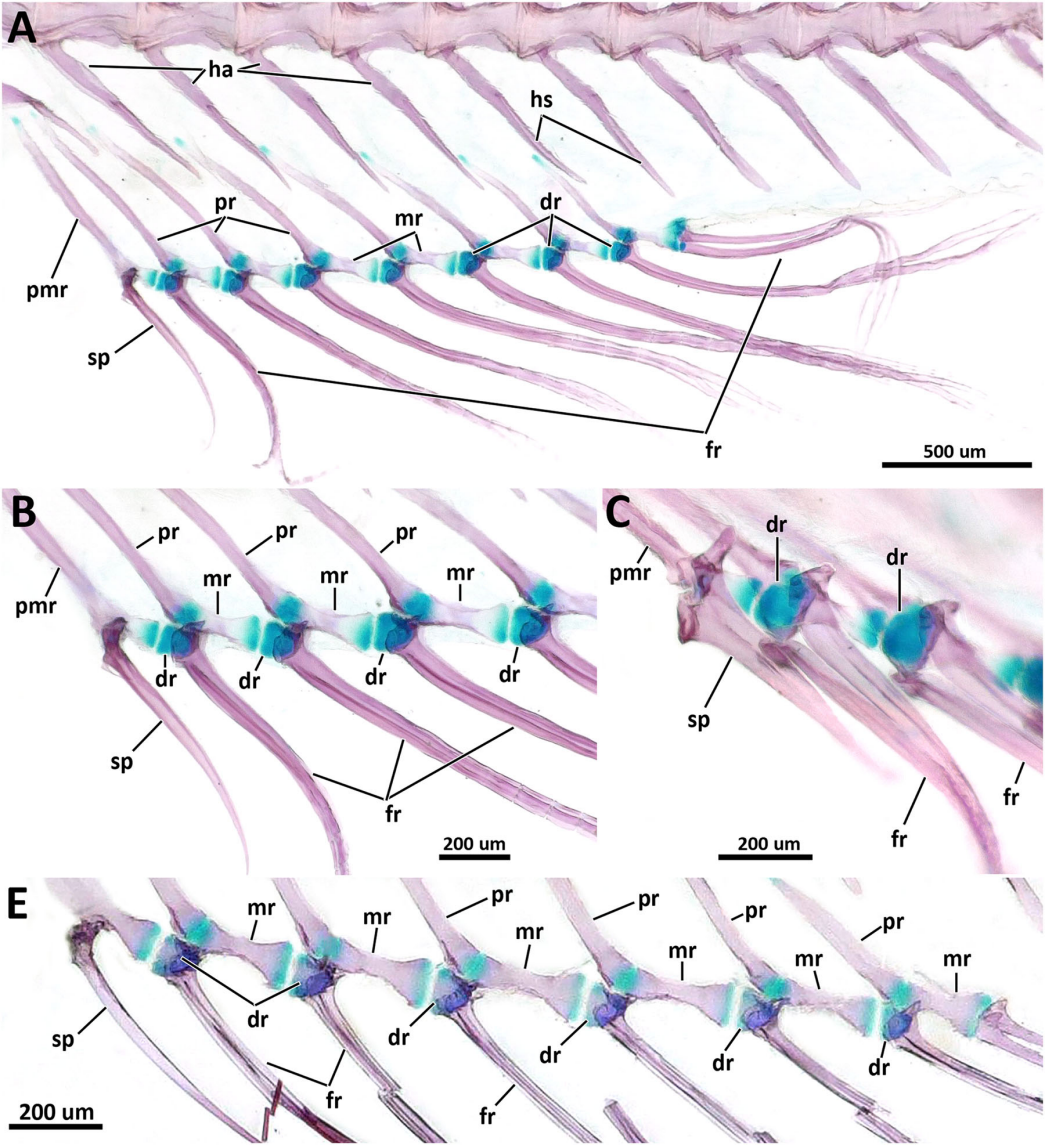
Anal fin of *Eviota* and *Sueviota*, represented by *Sueviota lachneri* (ROM 730CS, 16.6 mm SL; A–C) and *Eviota cometa* (MCZ 162965, 13 mm SL; D). Overall arrangement of the anal fin of *Sueviota lachneri* (A) and high magnification of the articulation between the spine and the proximal‐middle radial in lateral (B) and ventro‐lateral (C) views. (D) Close‐up image of *Eviota cometa* highlighting the partial ossification of distal radials. dr, distal radial; fr, fin ray; ha, haemal arch; hs, haemal spine; mr, middle radial; pmr, proximal‐middle radial; pr, proximal radial; sp, fin‐spine.

The anal fin of *Eviota* and *Sueviota* has one spine and seven to nine soft fin rays, with the last ray often branched to the base (Lachner and Karnella 1978, 1980; Jewett and Lachner 1983; Winterbottom and Hoese 1988; Greenfield and Winterbottom 2016; Tornabene et al. 2021). Seven to nine pterygiophores are present (Figure 13).

**Anal‐fin pterygiophores.** The first pterygiophore is a single elongated proximal‐middle radial, postero‐ventrally oriented, with its ventral edge projecting posteriorly, similar to a hockey‐stick shape. The anteroventral edge of the first proximal‐middle radial of the anal fin has a convex projection for articulation with the anal‐fin spine (Figure 13A,B). All other pterygiophores are posteroventrally oriented and have distinct proximal and middle radial bones (Figure 13). The first and the second anal‐fin pterygiophores insert between the tenth and eleventh haemal arches (interhaemal space 10; Figure 10A). Third, fourth, and fifth anal‐fin pterygiophores insert on interhaemal space 11, 12 and 13, respectively. The remaining pterygiophores will insert into the corresponding interhaemal space in a 1:1 or 2:1 pterygiophore/interhaemal space arrangement, depending on the species (see Table 2, 3, 4, 5). The last pterygiophore of the anal fin supports two soft rays (Figure 13A,E).

Anal‐fin proximal radials are rod‐shaped, with the ventral edge slightly wider than the dorsal edge. Anal middle radials are spool‐shaped. Distal radial cartilages are semi‐circular to trapezoidal, each radial supporting a soft anal‐fin ray (Figure 13C). *Eviota cometa*, like in its the dorsal fin, has distal radials of the anal with an ossified core in the middle of the element supporting anal‐fin rays one to five (Figure 13D). In *Eviota zebrina*, distal radials supporting anal‐fin rays one to six have an ossified core as well.

**Anal‐fin spines**. Anal‐fin spines are unsegmented, single, bilaterally fused bones (Figure 13). The anal‐fin spine is a supernumerary element, articulating with the anteroventral edge of the proximal‐middle bone that supports the first soft fin ray (Figure 13C). The length of the anal‐fin spine is relatively short, approximately two‐thirds of the length of the first soft fin ray (Figure 13A). The anterior margin of the spine has a shallow groove extending along the entire length (probably where the halves of the spine fused in early stages of development; Figure 13C). The anterior margin of base of the spine is convex centrally, forming a groove that articulates with the proximal‐middle radial. The anterior margin has two triangular processes on its anterior edges. The posterior margin projects posterolaterally, forming a conical process on the posterolateral edges.

### 3.9 Pectoral Girdle and Fins

**Figure 14:**
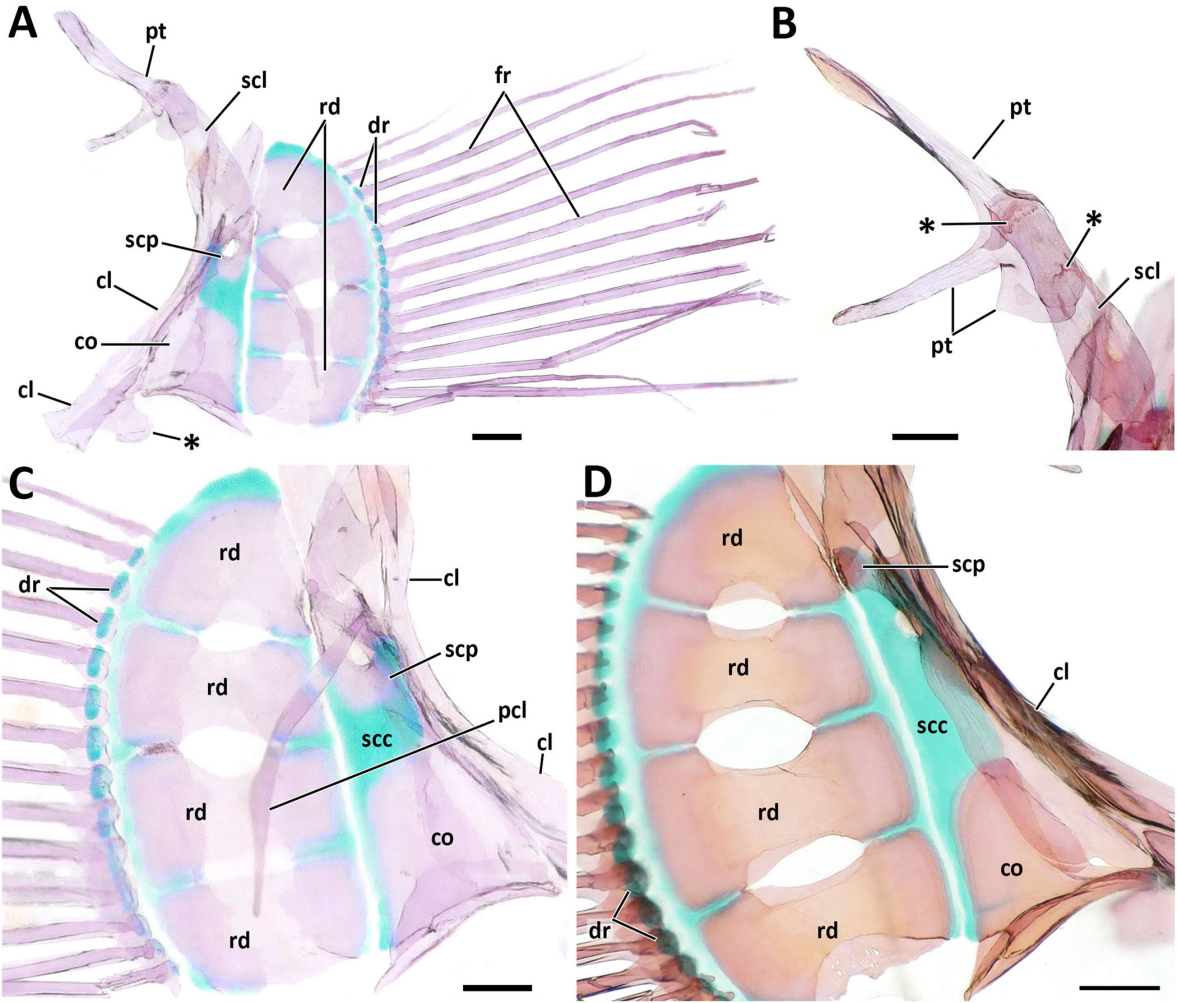
Morphology and variation of the pectoral fin. (A, C) *Eviota cometa* (MCZ 162965, 13 mm SL;). (B, D) *Eviota sparsa* (ANSP 148485, 16.1 mm SL). (A) Lateral view of the pectoral fin in *Eviota cometa* (MCZ 162965, 13 mm SL). Asterisk indicates the ventral process that articulates with the pelvic girdle. (B) Higher magnification of the dorsal region of the pectoral fin. Asterisks indicate the articulation between the posttemporal and supracleithrum. (C) Medial view of the pectoral fin of *Eviota cometa* (MCZ 162965, 13 mm SL). Note the scapula extending ventrally past the scapular foramina. (D) Medial view of the pectoral fin of *Eviota sparsa* (ANSP 148485, 16.1 mm SL). Note the scapula restricted to the dorsal edge of the scapulocoracoid cartilage, articulating exclusively with the dorsalmost basal radial. Scale bar, 200 µm. cl, cleithrum; co, coracoid, dr, distal radials; fr, fin rays; pcl, ventral postcleithrum; pt, posttemporal; rd, pectoral basal radial; scc, scapulocoracoid cartilage; scl, supracleithrum; scp, scapula.

**Posttemporal**. Posttemporal bone is the dorsalmost bone of the pectoral girdle and has a flattened, rectangular body with two anteriorly directed and vertically oriented processes (limbs; Figure 14A,B). The dorsal limb of posttemporal is the longest and projects anterodorsally. The anterior region of the dorsal limb expands laterally where it sits on the epioccipital. The ventral limb is slightly smaller than the dorsal limb, and projects anteroventrally, contacting the ventral region of the intercalar.

**Supracleithrum**. The supracleithrum is flattened, rectangular, and anterodorsally oriented (Figure 14A). The dorsal edge of the lateral surface of the supracleithrum contacts sagittally the medial surface of the ventral edge of the posttemporal at two points, one at the level of the base of the dorsal limb of the posttemporal, and another close to the ventral edge of the posttemporal (Figure 14B; for further details, see Holcroft and Wiley 2015). The medial margin of the ventral edge of the supracleithrum contacts the lateral margin of the dorsal region of the cleithrum.

**Cleithrum**. The cleithrum is robust, comma‐shaped, and posterodorsally oriented and is the largest bone of the pectoral girdle. The dorsal edge of the cleithrum is acute and dorsally oriented (Figure 14A). A wide posterodorsally oriented process arises from the dorsal region of the cleithrum; the width of its base is approximately twice that of the dorsal edge of the cleithrum. The shape of the posterior edge of the posterodorsal process of the cleithrum is variable. The posterior edge of the posterodorsal process of the cleithrum can be angular, forming an acute angle in some species (e.g., *Eviota sparsa*), or blunt, forming a rectangular margin, as observed in *E. cometa* (Figure 14A). The ventral edge of the cleithrum is straight. The right and left cleithra contact each other in the ventral midline. A small cartilage is present in the posterior margin of the region of contact of the ventral edges of the cleithrum. The ventromedial region of the cleithrum has a semicircular process to which the pelvic basipterygium is attached (Figure 14A).

**Ventral postcleithrum**. *Eviota* and *Sueviota* lack a dorsal postcleithrum, such as observed in other Gobiidae, Thalasseleotrididae, Oxudercidae and some xenisthmids (Goatley and Tornabene 2022). The ventral postcleithrum is a slender rod‐shaped bone, located medially to the cleithrum. *Eviota cometa* is the only species observed with the dorsal edge of the postcleithrum articulating with the medial surface of the cleithrum at the level of anterodorsal edge of the scapula (Figure 14C). In other examined species of *Eviota* and *Sueviota*, the postcleithrum does not contact the pectoral girdle; instead, this bone lays across the muscle fibers of the hypaxial muscles of the abdominal cavity (Figure 10A,B).

**Scapula**. Ossification of the scapula occurs in two conditions across *Eviota* and *Sueviota*. The scapula can be ellipsoidal and relatively large, almost replacing the entire dorsal half of the scapulocoracoid cartilage, encircling the scapular fenestra (e.g., *Eviota cometa*, *E. punctulata*; Figure 14A,C), or the scapula is short, restricted to the dorsal edge of the scapulocoracoid cartilage, with the scapular fenestra being entirely surrounded by cartilage (e.g., *E. sparsa, E. zebrina*; Figure 14D).

**Coracoid**. The coracoid is a trapezoidal and relatively large bone, its depth approximately 1.5 times that of the ventralmost pectoral radial. The anteroventral edge of the coracoid projects anteriorly, forming a rectangular process that contacts the cleithrum. On the posteroventral edge of the coracoid, a broad, triangular process projects posteriorly (Figure 14).

**Pectoral radial basal**. Four pectoral radial basals are present in *Eviota* and *Sueviota* (Figure 14). The dorsalmost basal is shaped as a broad circular sector. The two radial basals positioned centrally are spool‐shaped, whereas the ventralmost basal radial is rectangular. Pectoral distal radials are circular cartilages supporting pectoral‐fin rays. Fourteen to seventeen pectoral‐fin rays are present in *Eviota* (see also Lachner and Karnella 1980; Jewett and Lachner 1983; Greenfield and Winterbottom 2016; Greenfield 2021). Pectoral‐fin rays in species of *Eviota* can be branched or unbranched (Lachner and Karnella 1980; Jewett and Lachner 1983; Tornabene, Ahmadia, et al. 2013; Tornabene, Chen, and Pezold 2013; Tornabene et al. 2021). *Sueviota bryozophila* and *S. pyrios* have unbranched pectoral‐fin rays (Allen et al. 2016; Greenfield 2017), whereas *Sueviota aprica*, *S. atrinasa*, *S. lachneri*, *S. larsonae*, and *S. tubicola* have branched pectoral‐fin rays (Winterbottom and Hoese 1988; Allen and Erdmann 2017). *Sueviota minersorum* was described having only unbranched pectoral‐fin rays (Greenfield et al. 2019), however, photographs of its original description show the presence of at least one branched pectoral‐fin ray (Figures 1, 2 of Greenfield et al. 2019).

### 3.10 Pelvic Girdle and Fins

**Figure 15:**
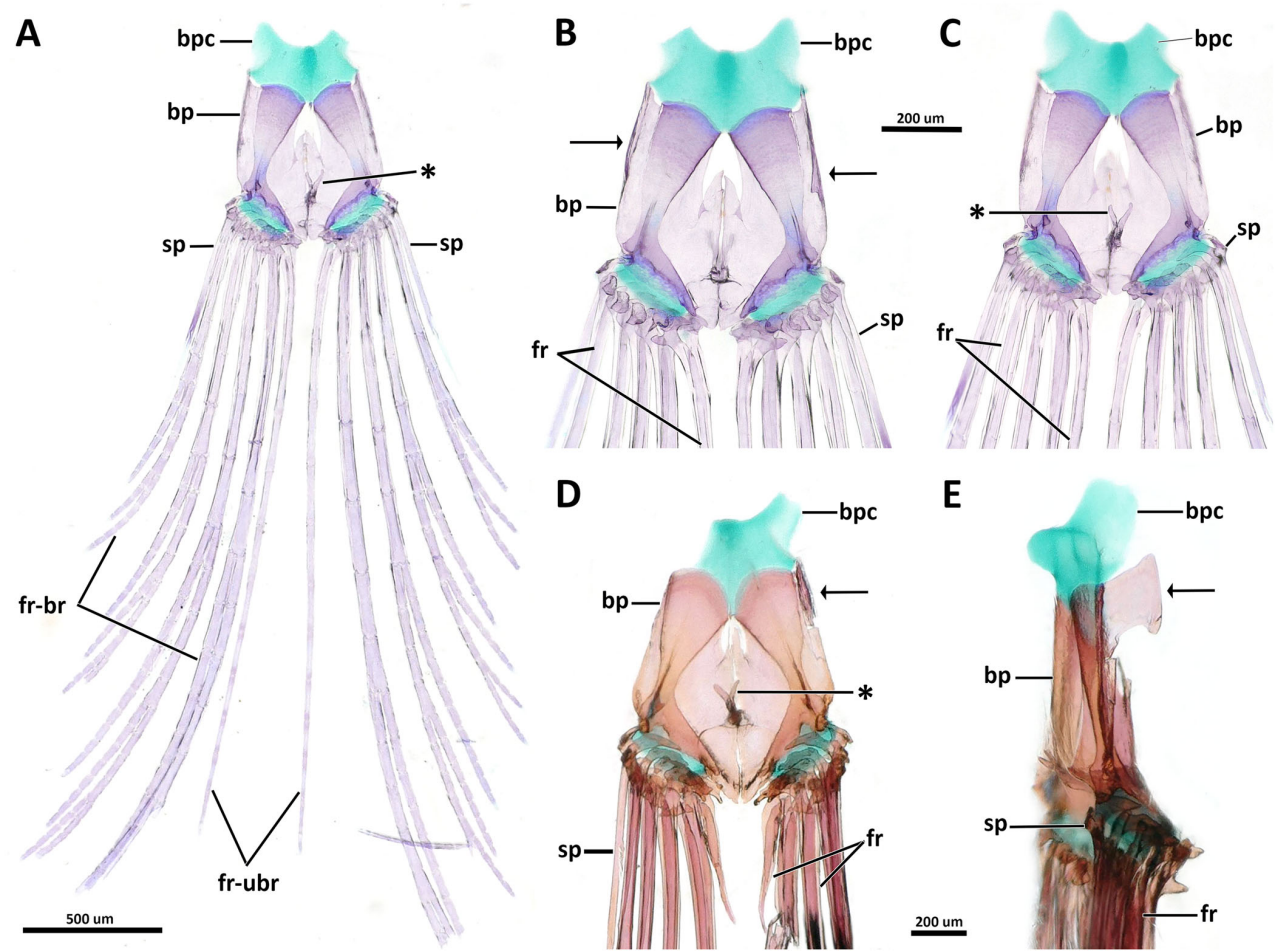
Pelvic fin morphology. (A–C) *Eviota sparsa* (ANSP 148481, 17.7 mm SL; A–C). (D, E) *Eviota zebrina* (ANSP 138917, 14.9 mm SL). (A–C) Overall shape of the pelvic girdle and fins of *Eviota sparsa* (ANSP 148481) in ventral view (A) and high magnification of the pelvic girdle in dorsal (B) and ventral view (C). Asterisk indicates the thin ventral process in the medial margins of the basipterygium. Black arrows indicate the process in lateral margin of the basipterygium. (D, E) High magnification of the pelvic girdle of *Eviota zebrina* (ANSP 138917, 14.9 mm SL) in ventral (D) and lateral (E) views. Note the reduced medialmost fin ray (5th), less than 10% of the fourth pelvic‐fin ray. Asterisk indicates the thin ventral process in the medial margins of the basipterygium. Black arrows indicate the process on lateral margin of the basipterygium. bp, basipterygium; bpc, basipterigial cartilage; br, branched; fr, fin ray; sp, spine; ubr, unbranched.

**Basipterygium**. Left and right basipterygium are rectangular, with cartilaginous anterior edges fused to each other medially (Figure 15). The medial margins of the left and right basipterygium are closely contacting, forming a distinct suture, but are not fused (Figure 15B–D). The lateral margin of the basipterygium is straight and projects dorsally, forming a narrow laminar projection (Figure 15E). The medial margin of the basipterygium has a deep triangular indentation in its anterior region and a thin process that projects ventrally in its posterior region. The anterior edge of the basipterygium bears a well‐developed cartilage with two anterior projections. These cartilaginous projections anchor the pelvic girdle to the ventro‐medial process of the cleithrum (Figure 15B–E). The posterior margin of the basipterygium is straight and has a cartilaginous site for articulating with pelvic‐fin rays and spine.

**Pelvic‐fin rays and spine**. The pelvic fin of *Eviota* and *Sueviota* has one spine and four or five fin rays. The spine is small, about one‐fourth of the length of the longest fin ray (usually the fourth; Figure 15A). Fin rays one to four are always branched in *Eviota* and most *Sueviota* (Figure 15), with variable branching patterns among species (see Greenfield and Winterbottom 2016). In *Sueviota*, the fifth fin ray is branched and its length ranges between 60% and 80% of the length of the fourth fin ray (see Winterbottom and Hoese 1988). *Sueviota bryozophila* is unique by having unbranched pelvic‐fin rays one to four, fin ray five remains branched as in other species of *Sueviota* (Allen and Erdman 2017). Branching of the fifth pelvic‐fin ray in *Sueviota aethon* is variable (Nunes Peinemann et al. 2024). The presence and length of the fifth fin ray is highly variable across *Eviota*: it ranges from being rudimentary (Figure 15D) or completely absent (e.g. *E. occasa*) to being 70% the length of the fourth pelvic ray, or longer (e.g. *E. sparsa*; Figure 15A; see further in Lachner and Karnella 1980; Jewett and Lachner 1983; Winterbottom and Hoese 1988; Greenfield and Winterbottom 2016; Greenfield 2021). In *Eviota*, when the fifth fin ray is present, it is always unbranched (except for *Eviota sparsa* which rarely displays branched fifth pelvic‐fin rays; see Jewett and Lachner 1983).

### 3.11 Caudal Fin

**Figure 16:**
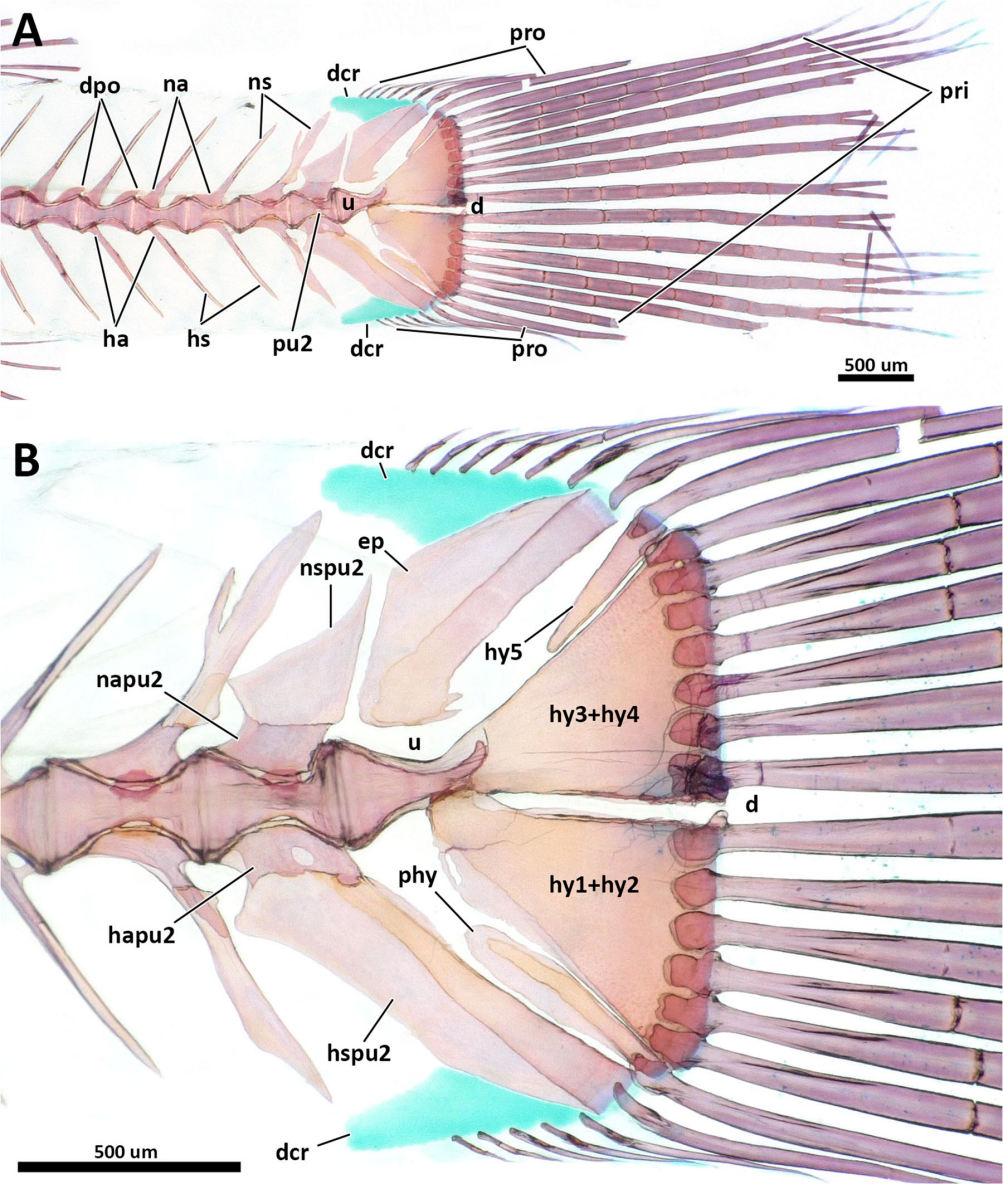
Caudal‐fin skeleton of *Eviota* and *Sueviota*, represented by *Eviota zebrina* (ANSP 138917, 14.9 mm SL). (A) Preural and ural skeletal elements, including caudal‐fin rays. (B) High magnification highlighting the ural elements. d, diastema; dcr, distal caudal radial cartilage; dpo, dorsal postzygapophysis; ep, epural; ha, haemal arch; hapu2, haemal arch of the second preural centrum; hs, haemal spine; hspu2, haemal spine of the second preural centrum; hy, hypural; na, neural arch; napu2, neural arch of the second preural centrum; nspu2, neural spine of the second preural centrum; phy, parhypural; pri, principal caudal‐fin rays; pro, procurrent caudal‐fin rays; pu2, second preural centrum; u, urostyle.

A total of 24 to 28 caudal fin rays are present across species of *Eviota* and *Sueviota*. The number of principal caudal fin rays varies from 14 to 17 (both inter‐ and intraspecifically). On the prepared specimens examined, the fused hypural one and two support six principal caudal fin rays, whereas the fused hypural three and four support seven principal fin rays (Figure 15A). Hypural five, epural bone, parhypural, and the haemal spine of the second preural centrum support a single caudal fin ray (variable whether from the principal or procurrent series). Remaining procurrent fin rays are supported by both dorsal and ventral distal caudal radial cartilages (Figure 16).

**Urostyle**. The urostyle (i.e., compound terminal centrum; see Schultze and Arratia 2013) of *Eviota* and *Sueviota* is spool‐shaped, with a deeply concave dorsal margin. Ventrally, the urostyle is fused with the fused hypurals one and two. The posterior edge of the urostyle is fused with hypurals three and four (Figure 16B).

**Hypurals and parhypural**. Three hypural bones are found in the caudal fin of *Eviota* and *Sueviota*. The ventralmost hypural is interpreted as a fused bone formed by hypurals one and two. Hypural one and two form a large, triangular, laterally compressed bone that occupies about a quarter of the urostylar region. The dorsal edge of hypural one and two is fused to the posteroventral margin of the urostyle. Dorsal to hypural one and two is another hypural bone that is interpreted as the fusion of hypurals three and four. Hypural three and four are fused with the posterior portion of the urostyle, triangular, laterally compressed, and as large as hypural one and two. The space between hypural one and two and hypural three and four delimits the diastema. Hypural five is an elongate, small bone located in the dorsoposterior edge of hypural three and four (Figure 16B). On the posteroventral margin of hypural one and two, an elongate, rectangular bone is present, which is interpreted here as the remains of the parhypural (Winterbottom 2003).

**Epural**. A single epural bone is present, a large trapezoidal shaped bone, as long as hypural three and four. A cartilaginous cap is present on the posterodorsal edge of the epural bone, supporting one procurrent fin ray (Figure 16).

**Distal caudal radial cartilages**. Two large, trapezoidal distal caudal radial cartilages are present. A dorsal distal caudal cartilage sits on the anterodorsal margin of the epural bone, supporting five to six procurrent caudal‐fin rays. The ventral distal caudal cartilage is also trapezoidal, and its posterodorsal margin contacts the anteroventral margin of the haemal spine of the second preural centrum. The ventral distal caudal cartilage supports four to six procurrent caudal fin rays (Figure 16).

**Second preural vertebra**. The second preural centrum is spool‐shaped. The neural arches of the second preural centrum are wide, approximately twice the length of the neural arch of the preceding vertebra. The neural spine of the second preural centrum, however, is short, approximately half the length of the neural spine in the preceding vertebra. The haemal arches of the second preural centrum are three times longer than the haemal arches of the preceding vertebra. Similarly, the haemal spine is large, rectangular, 1.5 longer and three times wider than the haemal spine of the preceding vertebra. At the base of the haemal spine, there is a triangular projection on its anterior margin (Figure 16).

## 4 Discussion

The skeleton of dwarfgobies *Eviota* and *Sueviota* described here are similar to the descriptions for *Eviota epiphanes* by Gosline (1955) and previous gobiid descriptions of *Istigobius, Microgobius*, *Afurcagobius*, *Trimma*, and *Kellogella* (Birdsong 1975; Murdy 1985; Gill 1993; Winterbottom 2003; Tornabene et al. 2018, respectively). Similar to *Trimma* (Winterbottom 2003) and *Kellogella* (Tornabene et al. 2018), the miniaturization of *Eviota* and *Sueviota* occurs due to a reduction in size and not due to skeletal truncations, as observed in *Paedocypris* and other paedomorphic species (Britz and Conway 2009). The number of bones and the extent of their ossification is similar to that observed in larger Gobiidae, and many other percomorphs.

*Eviota* and *Sueviota* share the character states proposed by Gill and Mooi (2012) as synapomorphies of Gobiidae: five branchiostegal rays with a single ray in the anterior region of the anterior ceratohyal; the presence of ventromedial process on ceratobranchial five; and the base of dorsal hemitrich of pelvic‐fin rays with a medial blade, an anteriorly directed process, and a laterally directed process for insertion of pelvic fin muscles and ligaments. *Eviota* and *Sueviota* also possess the T‐shaped palatine present in other species of Gobiidae, Oxudercidae, and the genus *Tempestichthys* (Thalasseleotrididae; Goatley and Tornabene 2022).

### 4.1 Phylogenetically Informative Characters Among “Coral Gobies” (*sensu* Thacker and Roje 2011)

*Eviota* and *Sueviota* are part of the “Coral Gobies” group of Thacker and Roje (2011), which superficially includes the genera *Bryaninops*, *Gobiodon, Kellogella, Larsonella, Lobulogobius, Lubricogobius, Luposicya, Minisicya, Paragobiodon, Phyllogobius*, and *Pleurosicya* (Thacker and Roje 2011; see also Tornabene et al. 2018). Most phylogenetic hypotheses of interrelationships for the species available within this clade show some combination of *Gobidon, Paragobiodon*, *Pleurosicya*, *Bryaninops* and *Eviota* forming a monophyletic clade group (Herler et al. 2009; Thacker and Roje 2011; Agorreta et al. 2013; Tornabene, Chen, and Pezold 2013; McCraney et al. 2020). The phylogenetic hypothesis of McCraney et al. (2020) included sequence data for one mitochondrial gene and one nuclear gene for species of *Eviota*, all of which came from previous studies by Tornabene, Ahmadia, et al. (2013), Tornabene, Chen, and Pezold (2013), and Tornabene et al. (2015, 2016). These studies all recovered the branched and unbranched clades as monophyletic. In contrast to trees by Tornabene, Ahmadia, et al. (2013) and Tornabene et al. (2015), which had limited gobiid outgroups, the phylogenetic tree recovered by McCraney et al. (2020) places both clades of *Eviota* in a polytomy with the group formed by *Gobiodon*, *Paragobiodon*, *Pleurosicya*, and *Bryaninops*, potentially casting doubts on the monophyly of *Eviota*.

**Figure 17:**
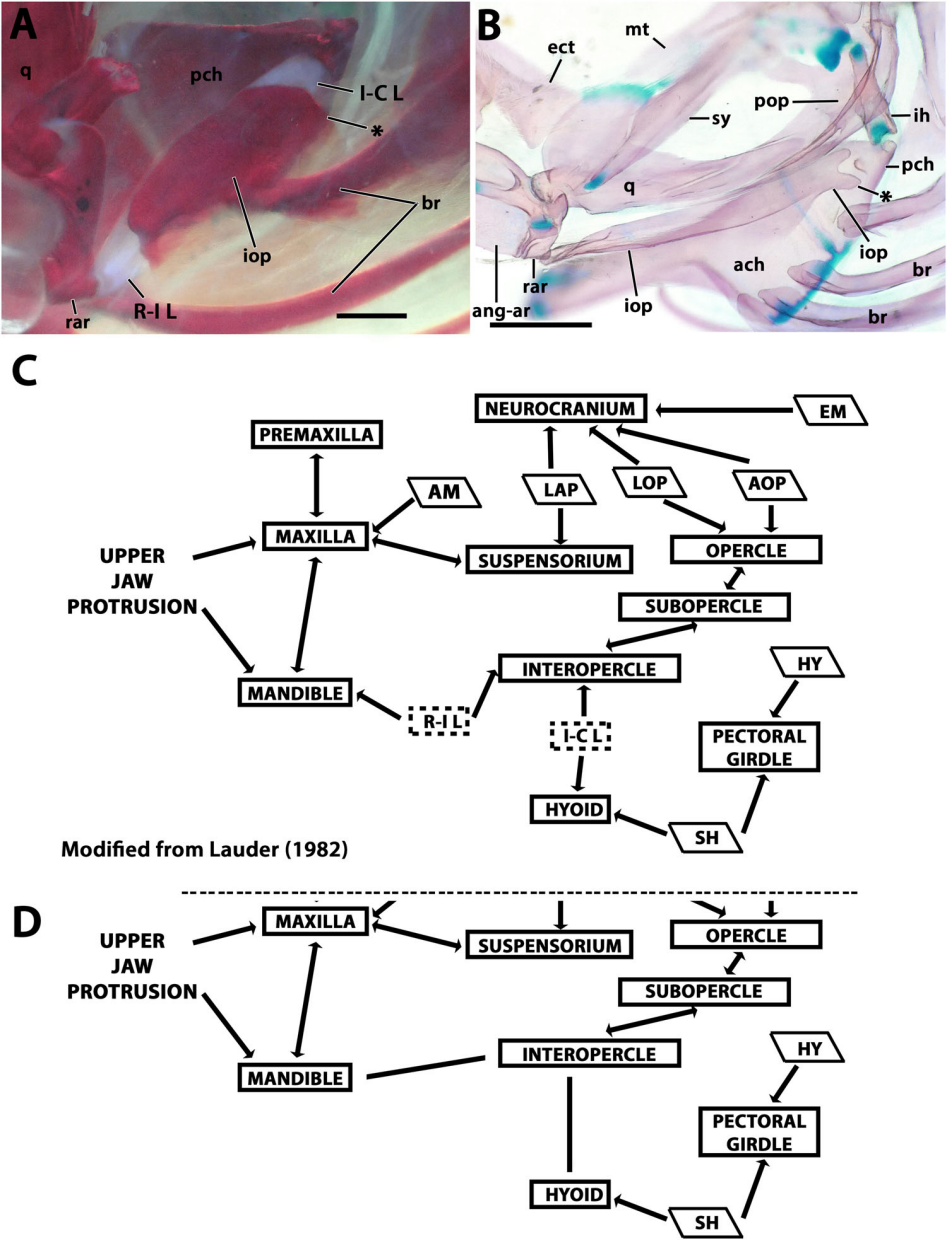
(A) Interopercular articulation mediated by ligaments observed in non‐coral reef gobies and teleosts in general, represented by *Benthophilus stellatus* (MCZ 46454). Note the presence of both retroarticular‐interopercle and interopercle‐ceratohyal ligaments. Asterisk indicates the straight posterior margin of the interopercle. The posteroventral process of the quadrate was removed to expose the interopercle. (B) Interopercle directly contacting both retroarticular and posterior ceratohyal bones, without ligaments, the condition observed in all species of *Eviota* and *Sueviota*, represented by *Sueviota lachneri* (ROM 730CS). Asterisk indicates the wrench‐shaped posterior end of the interopercle, exclusive to *Sueviota* and species of *Eviota* with branched pectoral‐fin rays. (C, D) Structural network of biomechanical pathways for jaw protrusion in the head of a generalized percomorph (C; modified from Lauder 1982) and *Eviota* and *Sueviota* (D). Dashed line in (D) indicates that the network above the maxilla, suspensorium, and opercle is the same in *Eviota* and *Sueviota* as represented for the generalized percomorph. Solid rectangles represent bony elements. Dashed rectangles represent ligaments. Parallelograms indicate muscles. Single‐headed arrows point from the muscle to its bony insertion. Double‐headed arrows indicate interaction between bones mediated by ligaments. Non‐headed bars indicate the direct contact among retroarticular, interopercle, and posterior ceratohyal (labeled simply as hyoid). Scale bar, 500 µm. ach, anterior ceratohyal; ang‐ar, anguloarticular; br, branchiostegal rays; ect, ectopterygoid; ih, interhyal; iop, interopercle; mt, metapterygoid; pch, posterior ceratohyal; pop, preopercle; q, quadrate; rar, retroarticular; sy, sympletic. Network abbreviations: AM, *adductor mandibulae* muscle complex; AOP, *adductor operculi* muscle; EM, epaxial muscles; HY, hypaxial muscles; LAP, *levator arcus palatini* muscle; LOP, *levator operculi* muscle; I‐C L, interopercle‐ceratohyal ligament; R‐I L, retroarticular‐interopercle ligament; SH, sternohyoideus.

**Figure 18:**
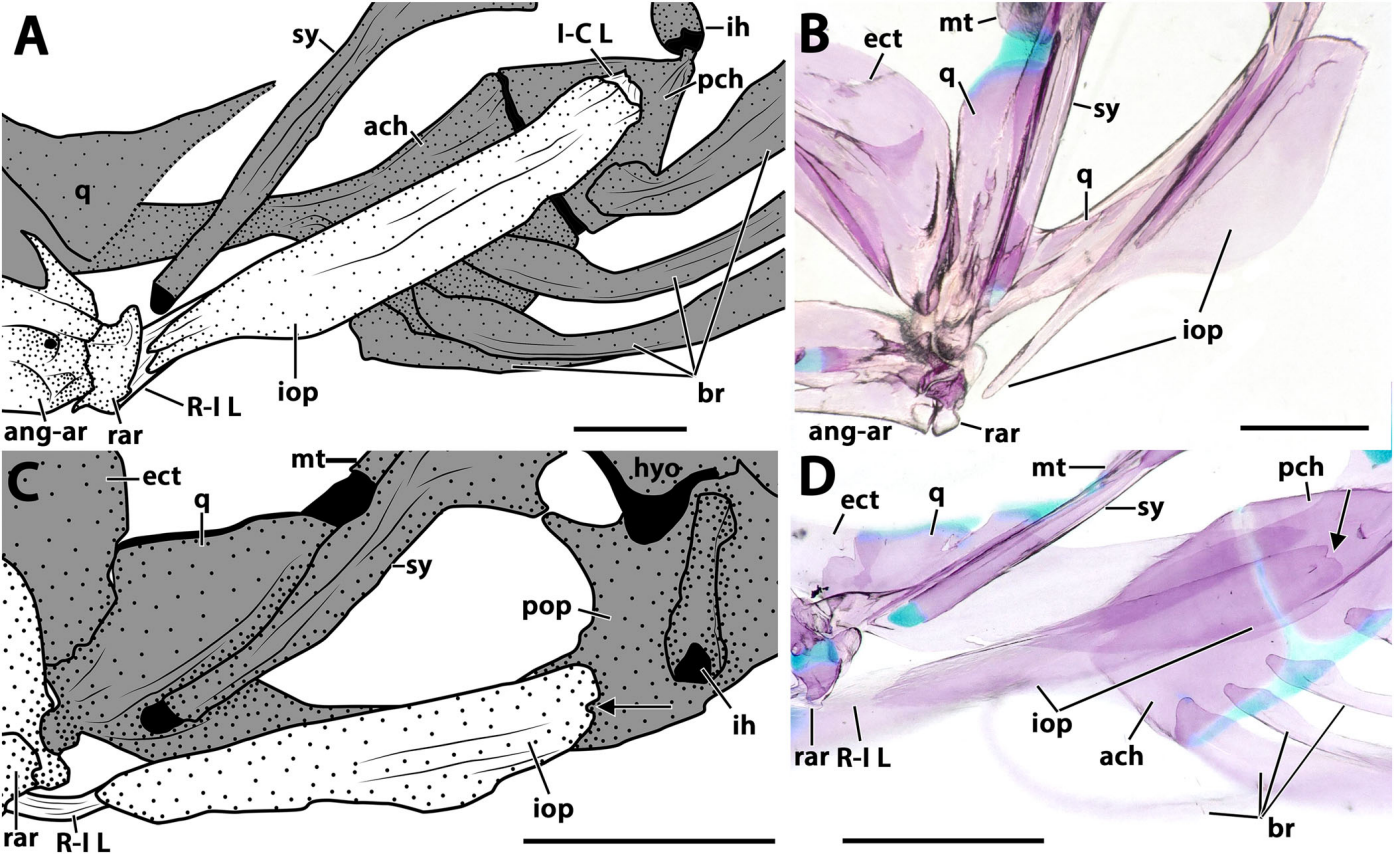
Comparative morphology of the interopercle. (A) *Microgobius signatus* (MCZ 30602). Dashed line indicates the location where the posteroventral process of the quadrate was removed. (B) *Gobiodon rivulatus* (MCZ 63137). (C) *Pleurosicya micheli* (MCZ 162627). (D) *Bryaninops amplus* (MCZ 84525). Black arrows indicate the notch on the posterior margin for articulation with the posterior ceratohyal. Scale bar, 500 µm. ach, anterior ceratohyal; ang‐ar, anguloarticular; br, branchiostegal rays; ect, ectopterygoid; I‐C L, interopercle‐ceratohyal ligament; ih, interhyal; iop, interopercle; mt, metapterygoid; pch, posterior ceratohyal; pop, preopercle; q, quadrate; rar, retroarticular; R‐I L, retroarticular‐interopercle ligament; sy, sympletic.

The monophyly and relationships among the genera included in Thacker and Roje's (2011) “Coral Gobies” and Agorreta et al.'s (2013) “Gobiodon‐lineage” have been inferred only by genetic data, and only for a small subset of the potential genera involved. Aside from data on *Eviota* and *Sueviota* presented here, morphological descriptions are available only for *Gobiodon, Paragobiodon*, and *Kellogella* (Gosline 1955; Harold and Winterbottom 1999; Tornabene et al. 2018). Complete descriptions of the skeleton of these genera are forthcoming, but this study already reports some characters that are potentially phylogenetically informative. Another significant morphological feature in the interopercle is the presence or absence of the posteroventral concavity that forms the notch. In *Gobiodon*, the ventroposterior edge of the interopercle has a continuous, straight margin. This is the condition observed in the other examined representatives of Gobiidae (Birdsong 1975; Murdy 1985; Winterbottom 2003; this study) and can be interpreted as the plesiomorphic state (Figures 17A and 18A,B). In *Bryaninops*, *Pleurosicya*, *Paragobiodon*, *Eviota*, and *Sueviota*, the posteroventral margin of the interopercle is concave, forming a notch for contacting with the posterior ceratohyal (Figure 18C,D). If we disregard any previous phylogenetic hypothesis to optimize the evolutionary history of this variation, the presence of a concavity in the posteroventral edge of the interopercle could support *Bryaninops*, *Pleurosicya*, *Paragobiodon, Eviota*, and *Sueviota* forming a monophyletic group. Conversely, mapping the states of this character in McCraney et al. (2020) hypothesis, the concavity in the posteroventral edge of the interopercle would be recovered as a synapomorphy for a clade containing all of these genera (*Gobiodon, Bryaninops*, *Pleurosicya*, *Paragobiodon, Eviota*, and *Sueviota*), with a secondary reversion in *Gobiodon*.

**Figure 19:**
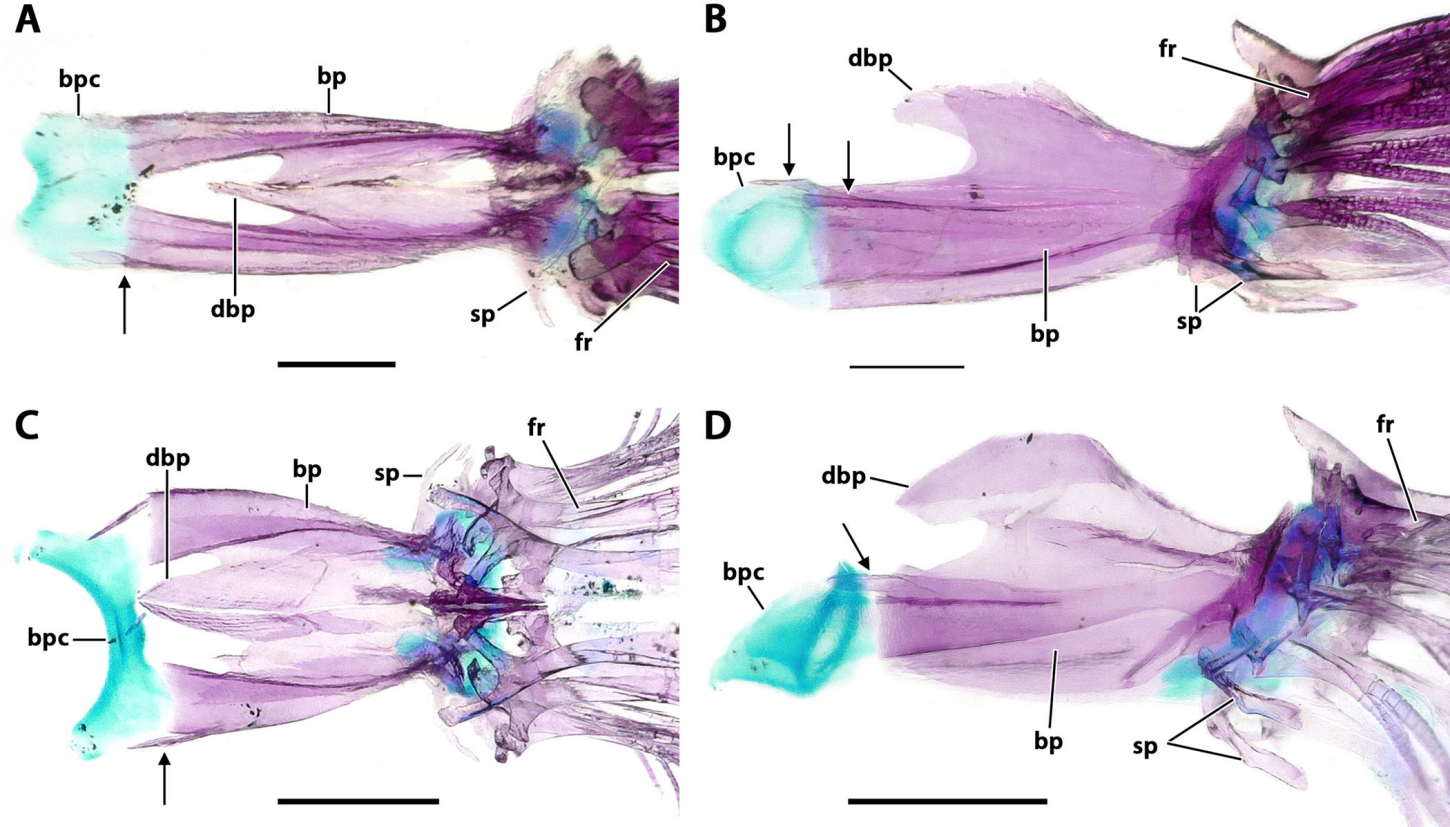
Pelvic girdle of other coral‐reef gobies, represented by *Gobiodon rivulatus* (A, B; MCZ 63137) and *Bryaninops amplus* (C, D; MCZ 84525) in dorsal (A, C) and lateral (B, D) views. Note the enlarged wavelike dorsal pelvic process formed by the medial margins of the the basipterygium bones. Black arrows indicate the process on the lateral margin of the basipterygium for articulation with the pectoral girdle. Scale bar, 500 µm. bp, basipterygium; bpc, basipterygial cartilage; dbp, dorsal process of the basipterygium; fr, fin rays; sp, fin spine.

Another phylogenetically informative character among “Coral Gobies” is the shape of the pelvic girdle. The basipterygia of the pelvic fin of *Gobiodon*, *Paragobiodon*, *Pleurosicya*, and *Bryaninops* have their medial margins projecting anterodorsally, forming a large, wavelike dorsal pelvic process for muscle attachment (Figure 19), similar to that described by Winterbottom (2003) for *Trimma*. In *Eviota* and *Sueviota*, the medial margins of the basipterygia lack any dorsal projections, forming a simple, laminar bone (Figure 15E), similar to the accounts of *Microgobius* and *Istigobius* (Birdsong 1975; Murdy 1985). Assuming the current phylogenetic hypothesis for coral‐reef gobies from McCraney et al. (2020), the presence of these large dorsal pelvic processes could be a putative synapomorphy grouping *Gobiodon*, *Paragobiodon*, *Pleurosicya*, and *Bryaninops*.

### 4.2 Potential Synapomorphy of *Eviota* and *Sueviota*

*Eviota* and *Sueviota* are unique for having the anterior edge of the interopercle contacting the retroarticular directly without the retroarticular‐interopercular ligament (Figures 5, 7, and 17). The anterior edge of the interopercle of *Gobiodon*, *Paragobiodon*, *Pleurosicya*, and *Bryaninops*, as well the remaining examined representatives of Gobiidae (and Teleostei; Gosline 1955; Birdsong 1975; Lauder 1982; Murdy 1985; Winterbottom 2003; Ferry and Hernandez 2011; Vaz and Hilton 2023) do not contact the retroarticular directly; instead the connection between these bones is made through the retroarticular‐interopercular ligament (Figures 17A and 18). This unique modification could represent a potential synapomorphy supporting a monophyletic *Eviota* (both branched and unbranched clades) and *Sueviota*.

Winterbottom and Hoese (1988) illustrated the oral jaws, suspensorium, and opercular bones of *Sueviota* bearing a retroarticular‐interopercular ligament connecting the lower jaw to the interopercle bone, despite not labelling this ligament in their illustration. Reexamination of Winterbottom and Hoese's (1988) cleared‐and‐stained specimens (Figure 17B) confirmed that the retroarticular‐interopercular ligament is absent in *Sueviota* and the anterior edge of the interopercle contacts the retroarticular directly.

### 4.3 Potential Synapomorphy Grouping the Branched Clade of *Eviota* and *Sueviota*

In all examined specimens of *Eviota* and *Sueviota*, the posteroventral margin of the posterior edge of the interopercle is always concave and forms a notch (Figure 7). In species of *Eviota* with unbranched pectoral‐fin rays, the posteroventral concavity is shallow (Figure 7A,B), similar to that described by Winterbottom and Hoese (1988). Across species of *Eviota* with branched pectoral‐fin rays, as well all examined species of *Sueviota*, the posteroventral margin of the posterior edge of the interopercle has a deep concavity and an additional posteroventral process, forming a wrench‐shaped edge (Figure 7C–F). Winterbottom and Hoese (1988) described the additional posteroventral process of the interopercle as a lobe, however, they detailed its presence only in the brached clade of *Eviota* and that *Sueviota* would have only a notch in the posterior margin of the interopercle. Their illustration of the interopercle of *Sueviota lachneri* (Figure 2 of Winterbottom and Hoese 1988), however, depicts a wrench‐shaped posterior edge of the interopercle. Our examined sample, which includes the material of *Sueviota* examined by Winterbottom and Hoese (1988), confirmed that all species of the branched clade of *Eviota* (Figure 7C–F) and *Sueviota* (Figure 17B) have the distinct posteroventral process in the posterior margin of the interopercle that forms the wrench‐shaped condition.

All other representatives of Gobiidae examined (Birdsong 1975; Murdy 1985; Winterbottom 2003; this study), similar to the species of *Eviota* with unbranched pectoral‐fin rays, lack the posteroventral process on the interopercle (Figure 7A,B). Therefore, the presence of the posteroventral process, resulting in a wrench‐shaped interopercle (Figures 7C–F and 17B), can be interpreted as a potential synapomorphy grouping the species with branched pectoral‐fin rays of *Eviota* and *Sueviota*. This inference, however, suggests that *Sueviota* is nested within *Eviota* instead of being a separate genus. The validity of *Sueviota*, however, requires further investigation, incorporating broader taxonomic sample and genetic data.

### 4.4 Potential Synapomorphy Within Groups of the Unbranched Clade of *Eviota*

Across the diversity of *Eviota* and *Sueviota*, the mesethmoid is present in two conditions. One condition is a complex and large mesethmoid, with a transversal, laminar drop‐shaped anterior portion (located between the lateral ethmoids) and a trapezoidal, longitudinally arranged posterior portion that extends posteriorly across the anterior region of the interorbital region (Figure 3A–E). This condition was observed in all examined species of *Sueviota* and most of the examined species of *Eviota*, occurring both in species with branched (e.g., *E. sparsa*, *E. atauroensis*) and unbranched pectoral‐fin rays (e.g., *E. zebrina*, *E. cometa*). This condition is present in *Gobiodon*, *Paragobiodon*, *Pleurosicya*, *Kellogella*, as well as in other Gobiidae species outside Agorreta et al.'s (2013) “Gobiodon‐lineage” (Birdsong 1975; Murdy 1985; Winterbottom 2003; Tornabene et al. 2018; this study). The second condition is the mesethmoid being formed only by its anterior portion, a laminar, transversal, drop‐shaped bone (Figure 3F–M). The posterior portion of the mesethmoid does not ossify, with the posterior corresponding region being formed by the ethmoid cartilage (Figure 3G). This condition is observed only in eight species with unbranched pectoral‐fin rays (i.e. in the “unbranched clade” *sensu* Tornabene, Ahmadia, et al. 2013; Tornabene, Chen, and Pezold 2013): *E. prasites*, *E. pellucida*, *E. bifasciata*, *E. atriventris*, *E. lachdeberei*, *E. nigriventris*, *E. infulata*, and *E. seebrei*. A similar condition was also observed only in *Bryaninops* and *Afurcagobius* (Gill 1993).

Optimizing the distribution of these morphological conditions according to Tornabene et al. (2016) and McCraney et al. (2020), the complex mesethmoid with anterior and posterior portions seems like the plesiomorphic condition for dwarfgobies, as this condition was present widely across the diversity of Gobiidae. The loss of the ossification of the posterior portion of the mesethmoid, therefore, could be a potential synapomorphy grouping *E. prasites*, *E. pellucida E. bifasciata*, *E. atriventris*, *E. lachdeberei*, *E. nigriventris*, *E. infulata*, and *E. seebrei*; with a homoplastic occurrence in *Bryaninops*. Indeed, most of these species of *Eviota* form a well‐supported monophyletic group within the unbranched‐clade, based on molecular phylogenies by Tornabene et al. (2015, 2016; position of *E. infulata* is unresolved). Species that have the mesethmoid with both anterior and posterior portions (*Eviota sigillata, E. storthynx, E. zebrina, E. shimadai*) were recovered in separate clade than those species that lack the posterior portion of the mesethmoid (*E. lachdeberei, E. nigriventris, E. bifasciata, E. atriventris, E. infulata, E. prasites, E. pellucida, E. seebrei*; Tornabene et al. 2015, 2016). It is possible that the distribution of the reduced mesethmoid might be larger than that observed in our sample.

### 4.5 Comments on Previous Morphological Support for the Branched and Unbranched Clades

Tornabene, Ahmadia, et al.'s (2013) phylogenetic analyses recovered two monophyletic groups within *Eviota* with a clear morphological distinction: one clade was formed by species that have at least one branched pectoral‐fin ray and another by species that possess only unbranched pectoral fin rays, coining the term branched and unbranched clades, followed throughout this manuscript. Although Tornabene, Ahmadia, et al.'s (2013) ancestral reconstruction could not directly establish the ancestral state of this character for *Eviota*, given the wide distribution of branched pectoral fin rays in the “Gobiodon‐lineage” outgroups (*sensu* Agorreta et al. 2013), they inferred that the plesiomorphic condition for *Eviota* was branched pectoral fin rays. This present osteological study found a similar distribution of this character, further suggesting that unbranched pectoral‐fin rays are a supporting synapomorphy for the unbranched clade of *Eviota*.

Lachner and Karnella (1980) and Jewett and Lachner (1983) showed that species of *Eviota* with unbranched pectoral‐fin rays usually have 25 vertebrae, whereas species with branched pectoral‐fin rays have 26 vertebrae. Winterbottom and Hoese (1988) confirmed the distribution of these morphological features in *Eviota* and further suggested the existence of two groups, with *Sueviota* potentially being part of the group of species with branched pectoral fin rays and 26 vertebrae. Similar to the optimization of pectoral‐fin branching, the total number of vertebrae has a similar distribution. Besides species of *Sueviota* and the branched clade of *Eviota*, the examined outgroups of the “coral gobies” *Bryaninops*, *Pleurosicya*, *Gobiodon*, and *Paragobiodon* all have 26 vertebrae, suggesting that this condition is likely the plesiomorphic state for dwarfgobies. Therefore, a reduction of one vertebra, resulting in 25 total vertebrae, is a potential synapomorphy supporting the unbranched clade.

Winterbottom and Hoese (1988) suggested that the sphenotic of species from the branched clade (*sensu* Tornabene, Chen, and Pezold 2013) is elongated, posteriorly reaching the supraoccipital, whereas the sphenotic of species of the unbranched clade is not elongated and does not reach the supraoccipital. This variation, however, was not observed in any specimen of *Eviota* or *Sueviota*. All examined specimens have the sphenotic extending posteriorly, with their posteromedial edge contacting the anterolateral margin of the supraoccipital (Figures 2A and 4).

**Figure 20:**
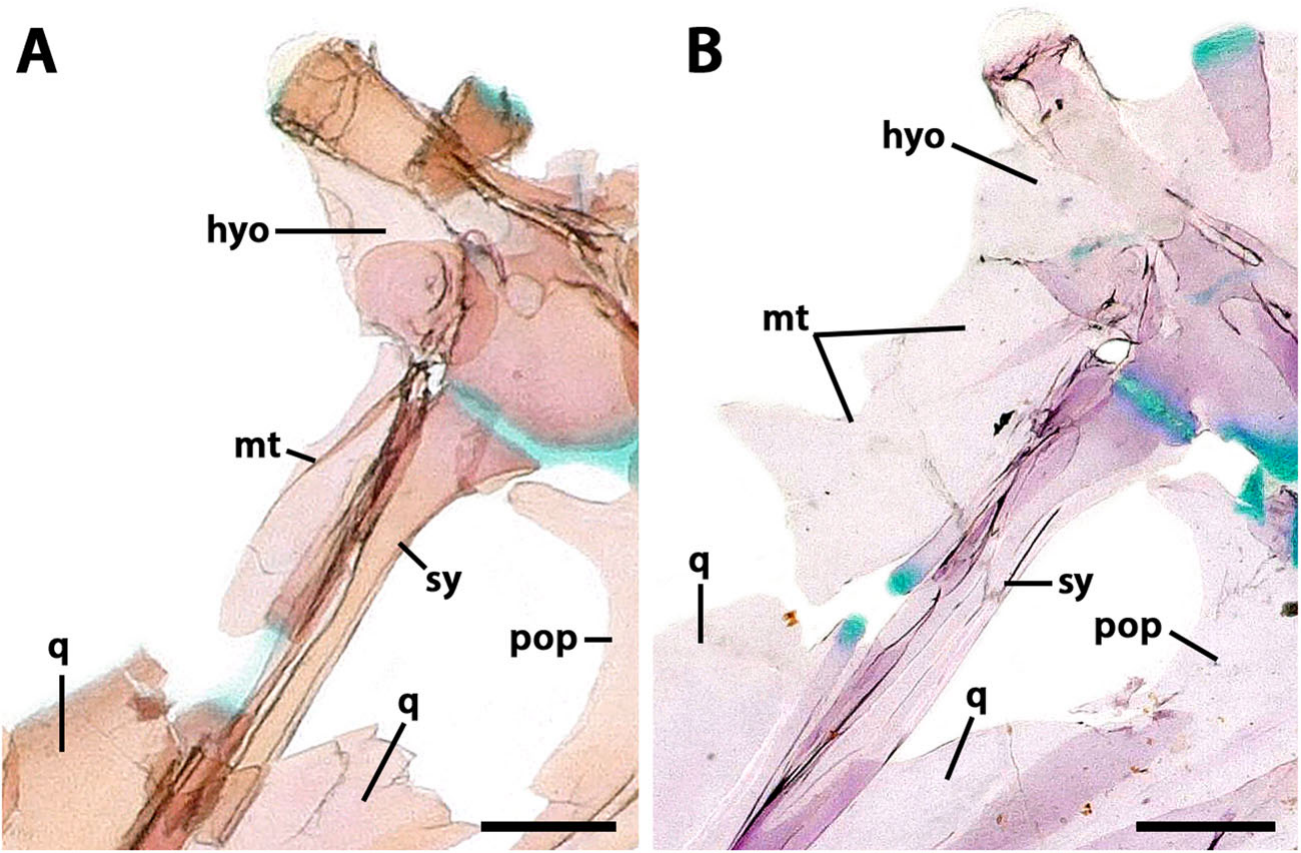
Variation of the metapterygoid in dwarfgobies. (A) *Eviota zebrina* (ANSP 138917, 14.9 mm SL). (B) *Eviota cometa* (MCZ 162965, 13 mm SL). Scale bar, 200 µm. hyo, hyomandibula; mt, metapterygoid; pop, preopercle; q, quadrate; sy, sympletic.

Winterbottom and Hoese (1988) also associated a reduced metapterygoid to species of the unbranched clade of *Eviota* and a well‐developed metapterygoid to *Sueviota* and the branched clade of *Eviota*. The examination of a larger diversity of taxa, however, could not recover such a relationship. For example, within the unbranched clade, the metapterygoid of *E. zebrina* is narrow and elongated, whereas the metapterygoid of *E. cometa* is relatively wider (Figure 20) than observed in *E. sparsa* (Figure 5C) and *E.* cf. *distigma* (Figure 6E). Similar variations were observed across the diversity of both clades (Figure 6). Winterbottom and Hoese (1988) also proposed that species of the unbranched clade have unmodified pelvic‐fin rays, whereas species of the branched clade have pelvic‐fin rays with numerous short lateral branches. The branching of pelvic‐fin rays across species of *Eviota* and *Sueviota* is indeed highly variable, but our data set did not find exclusive branch patterns in either branched or unbranched clades (see further details in Greenfield and Winterbottom 2016).

### 4.6 Reduction of Ligaments Associated to the Interopercle: Unknown Functional Implications

The loss of the retroarticular‐interopercle and interopercle‐ceratohyal ligaments in *Eviota* and *Sueviota* are particularly intriguing, as these ligaments are directly related to jaw mechanics and integration among jaws, hyoid, and opercular bones. Lauder (1982) demonstrates that these ligaments pull in different directions during the processes of buccal pumping and jaw protrusion (Figure 17C). With a shift from ligament‐mediated contact to direct bone‐to‐bone contact, we infer that the interopercle would be then pushed by the lower jaw or the posterior ceratohyal, instead of being pulled, although the system of links and leverages remain very similar (Figure 17D). A complete assessment of the implications resulting from the loss of both retroarticular‐interopercle and interopercle‐ceratohyal ligaments, however, is still necessary.

It is unclear whether the shift from ligament‐mediated to bone‐to‐bone contact in the interopercle is related to the miniaturization of *Eviota* and *Sueviota*. The loss of the retroarticular‐interopercle ligament seems to be a modification of these two genera unrelated to their size (Figure 17B), as other miniature “Coral Gobies” genera such as *Pleurosicya, Bryaninops*, *Paragobiodon* (Figure 18), as well the miniaturized *Trimma* (Winterbottom 2003), have the retroarticular‐interopercle ligament, similar to the other examined representatives of Gobiidae, and in fact most Teleostei (Figure 17A; see also Birdsong 1975; Murdy 1985; Lauder 1982; Ferry and Hernandez 2011). The loss of the interopercle‐ceratohyal ligament, conversely, has a broader distribution being absent in *Eviota, Sueviota*, and all other genera of “Coral Gobies” examined and it could potentially be related to the adaptation to smaller sizes of “Coral Gobies”. Additional supportive data for this statement, however, is needed. The miniaturized *Trimma*, however, as well the other examined cleared‐and‐stained specimens of Gobiidae, *Bathygobius soporator*, *Benthophilus stellatus*, *Chaenogobius gulosus*, *Gobiomorus dormitor, Microgobius signatus*, and in published descriptions of *Istigobius* (Birdsong 1975; Murdy 1985; Winterbottom 2003) have the posterior edge of the interopercle connected to the posterior ceratohyal by a ligament, the interopercle‐ceratohyal ligament.

## 5 Conclusions

The skeleton of *Eviota* and *Sueviota* is a rich source of phylogenetic information, providing phylogenetic insights at various taxonomic levels.

The unossified posterior portion of the mesethmoid is unique among *E. lachdeberei, E. nigriventris, E. bifasciata, E. atriventris, E. infulata, E. prasites, E. pellucida*, and *E. seebrei* and could be a potential synapomorphy grouping these eight species. Among the examined genera of the Gobiodon‐lineage, only *Bryaninops* has a similar condition and this occurrence is inferred to be homoplastic.

The reduction of one caudal vertebra resulting in 25 total vertebrae is present only in species of the unbranched clade of *Eviota* and is interpreted as a potential additional synapomorphy of the unbranched clade of *Eviota*.

The direct contact between the retroarticular and the anterior edge of the interopercle without the retroarticular‐interopercle ligament is present in all species of *Eviota* and *Sueviota*, being interpreted as a potential synapomorphy grouping these two genera.

All species of *Eviota* and *Sueviota*, as well *Bryaninops*, *Pleurosicya*, and *Paragobiodon*, have a concave, notched posterior edge of the interopercle. In species of the branched clade of *Eviota* and species of *Sueviota*, the concavity is deep and there is an additional posteroventral process, forming a wrench‐like posterior edge of the interopercle. This unique shape of the interopercle is inferred to be a potential synapomorphy grouping *Sueviota* with representatives of the branched clade of *Eviota*.

## Author Contributions

**Diego F. B. Vaz:** conceptualization, investigation, funding acquisition, writing – original draft, methodology, validation, visualization, writing – review and editing, formal analysis, data curation, resources. **Christopher H. R. Goatley:** writing – original draft, validation, writing – review and editing, resources. **Luke Tornabene:** writing – original draft, funding acquisition, validation, writing – review and editing, resources.

## Conflicts of Interest

The authors declare no conflicts of interest.

### Peer Review

The peer review history for this article is available at https://www.webofscience.com/api/gateway/wos/peer-review/10.1002/jmor.70039.

## Data Availability

The data supporting the results of this manuscript are available through direct (and reasonable) request to the corresponding author. CT‐scans used in this project are available on morphosource (https://www.morphosource.org/).

## Appendix 1 Comparative specimens of Gobiomorpharia examined

*Acanthogobius flavimanus*, MCZ 32473, CT‐scan.

*Acentrogobius cyanomus*, MCZ 33222, CT‐scan.

*Acentrogobius ferox*, MCZ 13149, CT‐scan.

*Amblyeleotris* sp., MCZ 46936, CT‐scan.

*Amblygobius phaelaena*, UWFC 156618, cleared‐and‐stained.

*Amblygobius sphynx*, MCZ 36706, CT‐scan.

*Amblychaeturichthys hexanemus*, MCZ 29017.

*Aphia minuta*, MCZ 13203, CT‐scan.

*Asterropteryx semipunctatus*, MCZ 36710, CT‐scan.

*Awaous aeneofuscus*, MCZ 41664, CT‐scan.

*Barbulifer ceuthoecuss*, MCZ 45890, CT‐scan.

*Bathygobius antilliensis*, MCZ 62340, CT‐scan.

*Bathygobius soporator*, MCZ 44636, cleared‐and‐stained.

*Benthophilus stellatus*, MCZ 46454, 2 specimens, cleared‐and‐stained.

*Boleophthalmus pectinirostris*, MCZ 13311, CT‐scan.

*Brachyamblyopus urolepis*, MCZ 33241, CT‐scan.

*Brachygobius doriae*, MCZ 56041, CT‐scan.

*Butis butis*, MCZ 56039, CT‐scan.

*Caffrogobius* sp., MCZ13280, CT‐scan.

*Chaenogobius gulosus*, MCZ 29045, cleared‐and‐stained.

*Cryptocentroides arabicus*, MCZ 90665, CT‐scan.

*Cryptocentrus cristatus*, MCZ 38515, CT‐scan.

*Ctenogobius* sp., MCZ 46482, CT‐scan.

*Ctenotrypauchen microcephalus*, MCZ 58305, CT‐scan.

*Deltenosteus quadrimaculatus*, MCZ 62871, CT‐scan.

*Dormitator latifrons*, MCZ 51517, CT‐scan.

*Elacatinus puncticulatus*, MCZ 173382, CT‐scan.

*Eleotrica cableae*, MCZ 29603, CT‐scan.

*Eleotris amblyopsis*, MCZ 32926, CT‐scan.

*Eleotris* sp., MCZ 33214, CT‐scan.

*Exyrias puntang*, MCZ 46985, CT‐scan; UWFC 156629, cleared‐and‐stained.

*Gobiomorphus basalis*, MCZ 46271, CT‐scan.

*Gobiosoma bosc*, VIMS 40920, cleared‐and‐stained.

*Gnatholepis* sp., MCZ 46945 (CT‐scan), MCZ 159213 (CT‐scan), MCZ 164901 (CT‐scan).

*Gnatholepis cauerensis*, MCZ 46947, CT‐scan.

*Gnatholepis thompsoni*, MCZ 47648, CT‐scan.

*Gymneleotris seminudus*, MCZ 44759, cleared‐and‐stained.

*Microgobius signatus*, MCZ 30602, cleared‐and‐stained.

*Mogurnda adspersa*, MCZ 33105, CT‐scan.

*Psammogobius biocellatus*, UWFC 156936, cleared‐and‐stained.

*Priolepis* BMNH 2024.12.13.4‐6, CT‐scan.

## References

[jmor70039-bib-0001] Agorreta, A. , D. San Mauro , U. Schliewen , et al. 2013. “Molecular Phylogenetics of Gobioidei and Phylogenetic Placement of European Gobies.” Molecular Phylogenetics and Evolution 69, no. 3: 619–633. 23911892 10.1016/j.ympev.2013.07.017

[jmor70039-bib-0002] Allen, G. R. , and M. V. Erdmann . 2017. “ *Sueviota tubicola*, a New Species of Coral‐Rreef Goby (Teleostei: Gobiidae) From Papua New Guinea.” Journal of the Ocean Science Foundation 25: 1–7.

[jmor70039-bib-0003] Allen, G. R. , M. V. Erdmann , and N. K. D. Cahyani . 2016. “ *Sueviota bryozophila*, a New Species of Coral‐Reef Goby From Indonesia (Teleostei: Gobiidae).” Journal of the Ocean Science Foundation 20: 76–82.

[jmor70039-bib-0004] Arratia, G. 2008. “Actinopterygian Postcranial Skeleton With Special Reference to the Diversity of Fin Ray Elements, and the Problem of Identifying Homologies.” In Mesozoic Fishes 4 – Homology and Phylogeny, edited by G. Arratia , H. P. Schultze , and M. V. H. Wilson , 49–101. Munich: Dr Friedrich Pfeil.

[jmor70039-bib-0005] Arratia, G. , and H.‐P. Schultze . 1991. “Palatoquadrate and Its Ossifications: Development and Homology Within Osteichthyans.” Journal of Morphology 208: 1–81. 29865508 10.1002/jmor.1052080102

[jmor70039-bib-0058] Baldwin, C. C. , and G. D. Johnson . 1993. “Phylogeny of the Epinephelinae.” Bulletin of Marine Science 52, no. 1: 240–283.

[jmor70039-bib-0006] Birdsong, R. S. 1975. “The Osteology of *Microgobius signatus* Poey (Pisces: Gobiidae), With Comments on Other Gobiid Fishes.” Bulletin of the Florida Museum of Natural History 19: 135–187.

[jmor70039-bib-0007] Birdsong, R. S. , E. O. Murdy , and F. L. Pezold . 1988. “A Study of the Vertebral Column and Median Fin Osteology in Gobioid Fishes With Comments on Gobioid Relationships.” Bulletin of Marine Science 42, no. 2: 174–214.

[jmor70039-bib-0008] Britz, R. , and K. W. Conway . 2009. “Osteology of *Paedocypris*, a Miniature and Highly Developmentally Truncated Fish (Teleostei: Ostariophysi: Cyprinidae).” Journal of Morphology 270: 389–412. 19107939 10.1002/jmor.10698

[jmor70039-bib-0057] Datovo, A. , and R. P. Vari . 2013. “The Jaw Adductor Muscle Complex in Teleostean Fishes: Evolution, Homologies and Revised Nomenclature (Osteichthyes: Actinopterygii).” PLoS One 8, no. 4: e60846. 10.1371/journal.pone.0060846. 23565279 PMC3614958

[jmor70039-bib-0009] Depczynski, M. , and D. R. Bellwood . 2005. “Shortest Recorded Vertebrate Lifespan Found in a Coral Reef Fish.” Current Biology 15, no. 8: R288–R289. 15854891 10.1016/j.cub.2005.04.016

[jmor70039-bib-0010] Depczynski, M. , and D. R. Bellwood . 2006. “Extremes, Plasticity, and Invariance in Vertebrate Life History Traits: Insights From Coral Reef Fishes.” Ecology 87, no. 12: 3119–3127. 17249236 10.1890/0012-9658(2006)87[3119:epaiiv]2.0.co;2

[jmor70039-bib-0011] Dingerkus, G. , and L. D. Uhler . 1977. “Enzyme Clearing of Alcian Blue Stained Whole Small Vertebrates for Demonstration of Cartilage.” Stain Technology 52: 229–232. 71769 10.3109/10520297709116780

[jmor70039-bib-0012] Ferry, L. A. , and L. P. Hernandez . 2011. “Bony Fish Cranial Muscles.” In Encyclopedia of Fish Physiology: From Genome to Environment, Volume 1, edited by Farrel , A. P. Cech , J. J. Jr , J. G. Richards , and E. D. Stevens , 463–470. London: Academic Press.

[jmor70039-bib-0013] Fink, W. L. , and S. H. Weitzman . 1974. “The So‐Called Cheirodontin Fishes of Central America With Descriptions of Two New Species (Pisces: Characidae).” Smithsonian Contributions to Zoology 172: 1–46.

[jmor70039-bib-0014] Fujiwara, K. , T. Suzuki , and H. Motomura . 2020. “Two New Dwarfgobies (Gobiidae) From Southern Japan: *Eviota amamiko* and *Eviota perspicilla* .” Ichthyological Research 67: 139–154.

[jmor70039-bib-0015] Gill, A. C. , and R. D. Mooi . 2012. “Thalasseleotrididae, New Family of Marine Gobioid Fishes From New Zealand and Temperate Australia, With a Revised Definition of Its Sister Taxon, the Gobiidae (Teleostei: Acanthomorpha).” Zootaxa 3266: 41–52.

[jmor70039-bib-0016] Gill, H. S. 1993. “Description of a New Genus of Goby From Southern Australia, Including Osteological Comparisons With Related Genera.” Records of the Western Australian Museum 16: 175–210.

[jmor70039-bib-0055] Gill, A. C. , and S. L. Jewett . 2004. “ *Eviota hoesei* and *E. readerae*, New Species of Fish From the Southwest Pacific With Comments on the Identity of E. corneliae Fricke (Perciformes Gobiidae).” Records of the Australian Museum 56, no. 2: 235–240.

[jmor70039-bib-0017] Goatley, C. H. R. , and L. Tornabene . 2022. “ *Tempestichthys bettyae*, a New Genus and Species of Ocean Sleeper (Gobiiformes, Thalasseleotrididae) From the Central Coral Sea.” Systematics and Biodiversity 20, no. 1: 1–15.36970113

[jmor70039-bib-0018] Gosline, W. 1955. “The Osteology and Relationships of Certain Gobioid Fishes, With Particular Reference to the Genera *Kraemeria* and *Microdesmus* .” Pacific Science IX: 158–170.

[jmor70039-bib-0019] Greenfield, D. W. 2017. “An Overview of the Dwarfgobies, the Second Most Speciose Coral‐Reef Fish Genus (Teleostei: Gobiidae: *Eviota*).” Journal of the Ocean Science Foundation 29: 32–54.

[jmor70039-bib-0054] Greenfield, D. W. 2021. Addendum to the 2016 Key to the Dwarfgobies (Teleostei: Gobiidae: Eviota). Zenodo. 10.5281/ZENODO.4458248.

[jmor70039-bib-0020] Greenfield, D. W. , M. V. Erdmann , and A. Teitelbaum . 2024. “ *Eviota bacata*, a New Dwarfgoby (Teleostei: Gobiidae) From New Caledonia.” Journal of the Ocean Science Foundation 41: 14–21.

[jmor70039-bib-0053] Greenfield, D. W. , M. V. Erdmann , and I. V. Utama . 2019. Sueviota minersorum, A New Species of Sponge‐Dwelling Goby (Teleostei: Gobiidae) From Misool, Raja Ampat Islands, Indonesia. Zenodo. 10.5281/ZENODO.3516352.

[jmor70039-bib-0021] Greenfield, D. W. , and S. L. Jewett . 2014a. “ *Eviota minuta*, a New Dwarfgoby From the Philippine Islands.” Journal of the Ocean Science Foundation 12: 12–17.

[jmor70039-bib-0022] Greenfield, D. W. , and S. L. Jewett . 2014b. “Two New Dwarfgobies From the Western Pacific (Teleostei: Gobiidae: *Eviota*).” Copeia 2014, no. 1: 56–62.

[jmor70039-bib-0023] Greenfield, D. W. , L. Tornabene , M. Gómez‐Buckley , and M. V. Erdmann . 2018. “ *Eviota maculosa*, a New Dwarfgoby From the Western Pacific Ocean (Teleostei: Gobiidae).” Journal of the Ocean Science Foundation 31: 18–31.

[jmor70039-bib-0052] Greenfield, D. W. , and R. Winterbottom . 2016. “A Key to the Dwarfgoby Species (Teleostei: Gobiidae: *Eviota*) Described Between 1871 and 2016.” Journal of the Ocean Science Foundation 24: 35–90.

[jmor70039-bib-0024] Harold, A. S. , and R. Winterbottom . 1999. “ *Gobiodon brochus*: A New Species of Gobiid Fish (Teleostei: Gobioidei) From the Western South Pacific, With a Description of Its Unique Jaw Morphology.” Copeia 1999, no. 1: 49–57.

[jmor70039-bib-0025] Herler, J. , S. Koblmüller , and C. Sturmbauer . 2009. “Phylogenetic Relationships of Coralassociated Gobies (Teleostei, Gobiidae) From the Red Sea Based on Mitochondrial DNA Data.” Marine Biology 156: 725–739.

[jmor70039-bib-0026] Hilton, E. J. 2011. “Bony Fish Skeleton.” In Encyclopedia of Fish Physiology: From Genome to Environment, Volume 1, edited by Farrel , A. P. Cech , J. J. Jr. , J. G. Richards , and E. D. Stevens , 434–448. London: Academic Press.

[jmor70039-bib-0056] Holcroft, N. I. , and E. O. Wiley . 2015. “Variation in the Posttemporal‐Supracleithrum Articulation in Euteleosts.” Copeia 103, no. 4: 751–770. 10.1643/cg-14-099.

[jmor70039-bib-0027] Jenkins, O. P. 1903. “Report on Collections of Fishes Made in the Hawaiian Islands, With Descriptions of New Species.” Bulletin of the U.S. Fish Commission 22: 415–511.

[jmor70039-bib-0028] Jewett, S. L. , and E. A. Lachner . 1983. “Seven New Species of the Indo‐Pacific Genus *Eviota* (Pisces: Gobiidae).” Proceedings of the Biological Society of Washington 96, no. 4: 780–806.

[jmor70039-bib-0029] Lachner, E. A. , and S. J. Karnella . 1978. “Fishes of the Genus *Eviota* of the Red Sea With Descriptions of Three New Species (Teleostei: Gobiidae).” Smithsonian Contributions to Zoology 286: 1–23.

[jmor70039-bib-0030] Lachner, E. A. , and S. J. Karnella . 1980. “Fishes of the Indo‐Pacific Genus *Eviota* With Descriptions of Eight New Species (Teleostei: Gobiidae).” Smithsonian Contributions to Zoology 315: 1–127.

[jmor70039-bib-0031] Lauder, G. V. 1982. “Patterns of Evolution in the Feeding Mechanism of Actinopterygian Fishes.” American Zoologist 22: 275–285.

[jmor70039-bib-0032] McCraney, W. T. , C. E. Thacker , and M. E. Alfaro . 2020. “Supermatrix Phylogeny Resolves Goby Lineages and Reveals Unstable Root of Gobiaria.” Molecular Phylogenetics and Evolution 151: 106862. 32473335 10.1016/j.ympev.2020.106862

[jmor70039-bib-0033] Murdy, E. O. 1985. “Osteology of *Istigobius ornatus* .” Bulletin of Marine Science 36, no. 1: 124–138.

[jmor70039-bib-0034] Nunes Peinemann, V. , L. Pombo‐Ayora , L. Tornabene , and M. L. Berumen . 2024. “The Grumpy Dwarfgoby, a New Species of *Sueviota* (Teleostei, Gobiidae) From the Red Sea.” ZooKeys 1212: 17–28. 39309170 10.3897/zookeys.1212.121135 PMC11413506

[jmor70039-bib-0035] Reichenbacher, B. , T. Přikryl , A. F. Cerwenka , P. Keith , C. Gierl , and M. Dohrmann . 2020. “Freshwater Gobies 30 Million Years Ago: New Insights Into Character Evolution and Phylogenetic Relationships of †Pirskeniidae (Gobioidei, Teleostei).” PLoS One 15, no. 8: e0237366. 32834000 10.1371/journal.pone.0237366 PMC7446829

[jmor70039-bib-0036] Sabaj, M. H. 2020. “Codes for Natural History Collections in Ichthyology and Herpetology.” Copeia 108, no. 3: 593–669.

[jmor70039-bib-0037] Schultze, H.‐P. , and G. Arratia . 2013. “The Caudal Skeleton of Basal Teleosts, Its Conventions, and Some of Its Major Evolutionary Novelties in a Temporal Dimension.” In Mesozoic fishes 5 –global diversity and evolution, edited by G. Arratia , H. P. Schultze , and M. V. H. Wilson , 187–246. München: Dr Friedrich Pfeil.

[jmor70039-bib-0038] Taylor, W. R. , and C. C. Van Dyke . 1985. “Revised Procedures for Staining and Clearing Small Fishes and Other Vertebrates for Bone and Cartilage Study.” Cybium 9: 107–120.

[jmor70039-bib-0039] Thacker, C. E. , and D. M. Roje . 2011. “Phylogeny of Gobiidae and Identification of Gobiid Lineages.” Systematics and Biodiversity 9, no. 4: 329–347.

[jmor70039-bib-0040] Tornabene, L. , G. N. Ahmadia , M. L. Berumen , D. J. Smith , J. Jompa , and F. Pezold . 2013. “Evolution of Microhabitat Association and Morphology in a Diverse Group of Cryptobenthic Coral Reef Fishes (Teleostei: Gobiidae: Eviota).” Molecular Phylogenetics and Evolution 66, no. 1: 391–400. 23099149 10.1016/j.ympev.2012.10.014

[jmor70039-bib-0041] Tornabene, L. , Y. Chen , and F. Pezold . 2013. “Gobies Are Deeply Divided: Phylogenetic Evidence From Nuclear DNA (Teleostei: Gobioidei: Gobiidae).” Systematics and Biodiversity 11, no. 3: 345–361.

[jmor70039-bib-0042] Tornabene, L. , B. Deis , and M. V. Erdmann . 2018. “Evaluating the Phylogenetic Position of the Goby Genus *Kelloggella* (Teleostei: Gobiidae), With Notes on Osteology of the Genus and Description of a New Species From Niue in the South Central Pacific Ocean.” Zoological Journal of the Linnean Society 183: 143–162.

[jmor70039-bib-0051] Tornabene, L. , D. W. Greenfield , and M. V. Erdmann . 2021. “A Review of the *Eviota zebrina* Complex, With Descriptions of Four New Species (Teleostei, Gobiidae).” ZooKeys 1057: 149–184. 10.3897/zookeys.1057.66675. 34552371 PMC8417026

[jmor70039-bib-0043] Tornabene, L. , S. Valdez , M. Erdmann , and F. Pezold . 2015. “Support for a “Center of Origin” in the Coral Triangle: Cryptic Diversity, Recent Speciation, and Local Endemism in a Diverse Lineage of Reef Fishes (Gobiidae: *Eviota*).” Molecular Phylogenetics and Evolution 82: 200–210. 25300452 10.1016/j.ympev.2014.09.012

[jmor70039-bib-0044] Tornabene, L. , S. Valdez , M. V. Erdmann , and F. L. Pezold . 2016. “Multi‐Locus Sequence Data Reveal a New Species of Coral Reef Goby (Teleostei: Gobiidae: *Eviota*), and Evidence of Pliocene Vicariance Across the Coral Triangle.” Journal of Fish Biology 88, no. 5: 1811–1834. 10.1111/jfb.12947. 27021219

[jmor70039-bib-0045] Vaz, D. F. B. , and E. J. Hilton . 2023. “Skeletal Ontogeny of the Plainfin Midshipman, *Porichthys notatus* (Percomorphacea: Batrachoidiformes).” Journal of Anatomy 242: 447–494. 36524549 10.1111/joa.13794 PMC9919480

[jmor70039-bib-0046] Vaz, D. F. B. , C. M. Martinez , S. T. Friedman , and P. P. Rizzato . 2022. “Morphological descriptions and taxonomy.” In Methods for Fish Biology, 2nd edition, edited by S. Midway , C. Hasler , and P. Chakrabarty , 153–206. Bethesda: American Fisheries Society.

[jmor70039-bib-0047] Winterbottom, R. 2003. “A New Species of the Gobiid Fish Trimma From the Western Pacific and Northern Indian Ocean Coral Reefs, With a Description of Its Osteology.” Zootaxa 218: 1–24.

[jmor70039-bib-0048] Winterbottom, R. , and D. W. Greenfield . 2021. “ *Eviota pseudaprica*, a New Dwarfgoby From the Western Pacific Ocean (Teleostei: Gobiidae).” Journal of the Ocean Science Foundation 35: 30–40.

[jmor70039-bib-0049] Winterbottom, R. , and D. F. Hoese . 1988. “A New Genus and Four New Species of Fishes From the Indo‐West Pacific (Pisces; Perciformes; Gobiidae), With Comments on Relationships.” Royal Ontario Museum, Life Sciences Occasional Paper 37: 1–17.

[jmor70039-bib-0050] Yun, S. M. , J. M. Park , and K. H. Han . 2020. “Osteological Development of the Larvae and Juvenile of *Luciogobius Grandis* (Pisces: Gobiidae).” Development & Reproduction 24, no. 2: 125–133. 32734129 10.12717/DR.2020.24.2.125 PMC7375978

